# Target Identification with Live-Cell Photoaffinity Labeling and Mechanism of Action Elucidation of ARN23765, a Highly Potent CFTR Corrector

**DOI:** 10.1021/acs.jmedchem.4c02654

**Published:** 2025-02-10

**Authors:** Elisa Romeo, Francesco Saccoliti, Riccardo Ocello, Angela Andonaia, Caterina Allegretta, Cristina Pastorino, Nicoletta Pedemonte, Federico Falchi, Onofrio Laselva, Tiziano Bandiera, Fabio Bertozzi

**Affiliations:** 1 Structural Biophysics Facility, Istituto Italiano di Tecnologia (IIT), Genova 16163, Italy; 2 D3-PharmaChemistry, Istituto Italiano di Tecnologia (IIT), Genova 16163, Italy; 3 Department of Pharmacy and Biotechnology, University of Bologna, Bologna 40126, Italy; 4 Computational and Chemical Biology, Istituto Italiano di Tecnologia (IIT), Genova 16163, Italy; 5 Department of Clinical and Experimental Medicine, University of Foggia, Foggia 71122, Italy; 6 U.O.C. Genetica Medica, Istituto Giannina Gaslini (IGG), Genova 16147, Italy

## Abstract

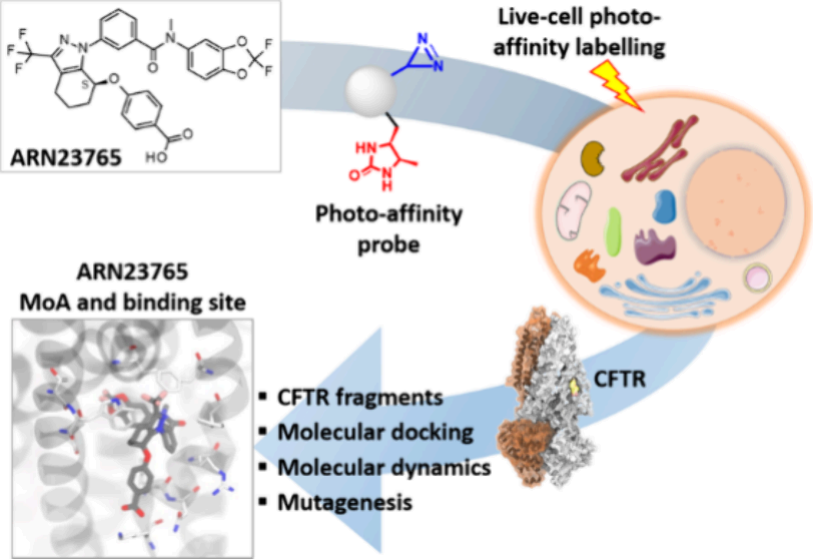

Molecular-targeted therapies for the treatment of cystic fibrosis (CF) rely on small-molecule modulators that rescue the activity of the defective CF transmembrane conductance regulator (CFTR) anion channel. **ARN23765** is a small molecule with subnanomolar potency in rescuing the function of mutant CFTR in bronchial epithelial cells from CF patients carrying the F508del-CFTR mutation. Considering the multifaceted interactions of CFTR with the plasma membrane and the complexity of the protein network within the cellular compartments, here we report the investigation of **ARN23765**’s molecular mechanism in live cells. We used the photoaffinity labeling (PAL) approach to demonstrate the interaction of **ARN23765**-derived probes with CFTR in cells. We showed that **ARN23765** contributes to F508del-CFTR rescue by stabilizing the membrane-spanning domain-1 and interacting with CFTR at the same site as other type I CFTR correctors. Our study characterizes **ARN23765**’s mode of action and highlights the potential of studying the interactions between CFTR and its correctors in live cells.

## Introduction

Cystic fibrosis (CF) is a multisystemic genetic disease, which primarily affects the respiratory and digestive systems. The progressive loss of lung function is the major cause of morbidity and mortality in people with CF (pwCF).^1^ CF is caused by mutations of the CF transmembrane conductance regulator (*CFTR*) anion channel gene, which impair the synthesis or function of the encoded CFTR protein. CFTR belongs to the ATP-binding cassette transporter (ABC) family of proteins. It is composed of two membrane-spanning domains (MSD1 and 2), two nucleotide-binding domains (NBD1 and 2) and a regulatory (R) domain. CFTR domains are connected by a series of intracellular loops (ICLs). Of these, the key ICL1 and ICL4 interact with NBD1, whereas ICL2 and ICL3 interact with NBD2.^2,3^ CFTR displays a unique ATP-gated anion channel activity, regulated by PKA- and PKC-dependent phosphorylation at multiple consensus sites in the R domain.^3,4^ PKA-dependent phosphorylation of CFTR promotes the dissociation of the R domain from inhibitory interactions, enabling ATP-mediated dimerization of NBD1 with NBD2 and thus allowing the opening of the channel.^3^ CFTR’s primary function is to transport chloride and bicarbonate anions across the apical membrane in epithelial cells.^5^ More than 2000 CFTR mutations have been reported so far, however to date only 1,085 have been definitively linked to CF (https://www.cftr2.org/; accessed on January 15th, 2025). Around 80% of pwCF carry at least one CFTR allele with the deletion of phenylalanine at position 508 (F508del).^6^ F508del-CFTR produces an incomplete glycosylated protein with a misfolding defect that is then retained in the endoplasmic reticulum (ER) where it is targeted for ubiquitin-dependent proteasomal degradation.^7^ Although F508del mutation is located in the NBD1, it does not only impair NBD1 folding. It has a wide-range impact on CFTR assembly, perturbing domain–domain interactions, thus impairing the stability of the entire protein.^3^ When F508del-CFTR is allowed to traffic to the plasma membrane, it displays a defective gating of the channel, with a longer time spent in the close state, and a decreased stability on the plasma membrane.^8,9^

**Figure 1:**
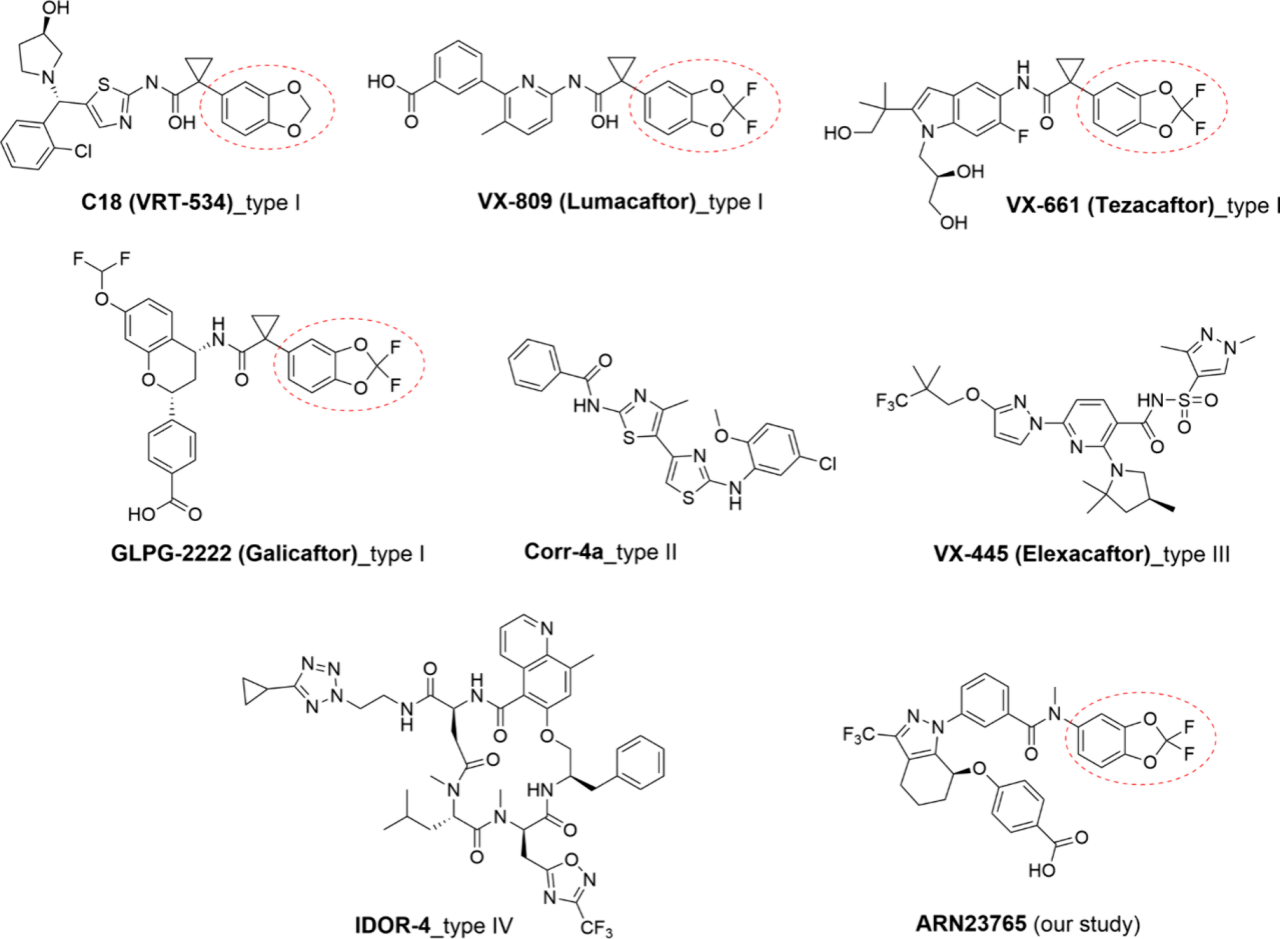
Known small-molecule CFTR correctors and their putative mode of action (benzodioxole and *gem*-difluorobenzodioxole moieties are highlighted with red dashed curves).

Over the past decade, regulatory agencies have approved a few small molecule-based therapies, referred to as CFTR modulators.^10^ Of these, correctors are compounds that increase the stability of mutant proteins during folding and assembly. By rescuing CFTR misfolding, processing, and trafficking, they increase channel density at the cell plasma membrane.^11^ CFTR correctors can work as pharmacological chaperones or as proteostasis regulators. Pharmacological chaperones influence mutant CFTR by stabilizing specific CFTR domains and/or by improving interactions between CFTR domains.^12^ In contrast, proteostasis regulators act on the protein synthesis/degradation machinery and thus beneficially affect CFTR processing.^13^ Correctors acting as pharmacological chaperones are classified based on their mode of action. Indeed, correctors may target the early folding of mutant CFTR, or they may display complementary mechanisms of action.^14^ Type I correctors suppress conformational defects within the interfaces between NBD1 and MSD1/MSD2; type II correctors target defects in NBD2; and type III correctors address NBD1 defects caused by F508del.^12,15^ Of the correctors approved for use in humans, VX-809 (lumacaftor) and VX-661 (tezacaftor) show a type I mechanism of action, while VX-445 (elexacaftor) is reported as a type III corrector (Figure 1).^16^ Recently it has been suggested that VX-445 does not specifically stabilize the NBD1 domain, but rather acts on domain interfaces, suggesting a different mechanism having an indirect, positive effect on NBD1 stability.^17^ Cryo-electron microscopy studies have helped elucidate the mechanism of action of these correctors and their interactions with purified CFTR.^18,19^ To date, there are no clinically approved type II correctors, although the type II corr-4a (Figure 1) was the first corrector demonstrated to exert activity in primary airway cells.^20^ A fourth type of corrector was proposed, IDOR-4 (Figure 1), targeting the “lasso” domain at the N-terminus of the CFTR protein, likely promoting cotranslational assembly of the lasso domain, MSD1, and MSD2.^21^

Recently, Pedemonte et al. reported the discovery of **ARN23765** (Figure 1), a preclinical stage compound with remarkable subnanomolar potency in rescuing CFTR function in bronchial epithelial cells from F508del-CFTR CF individuals.^22^ Preliminary data supported the hypothesis that **ARN23765** activity is compatible with that of type I correctors. Indeed, it shows additive effects in mutant CFTR rescue with type II and III correctors, but not with other type I correctors.^22^ Nevertheless, an extensive biological characterization of **ARN23765** has not yet been reported. The compound may reasonably be hypothesized to act by directly binding to F508del-CFTR and/or by modulating the activity of other proteins involved in the cell quality control system or the protein trafficking machinery. Photoaffinity labeling (PAL) has emerged as a cutting-edge chemical biology strategy to identify biological targets and/or interrogate the mechanism of action of small molecules, even in native cellular systems.^23^ PAL relies on the design and synthesis of photoaffinity probes (PAPs) that feature key reactive groups in their structure, while retaining a reasonable binding affinity and selectivity toward the putative target protein(s). The parent compound is chemically modified to install two functional groups: a photoactive moiety, which allows covalent cross-linking to the target protein(s) when irradiated with light of a proper wavelength, and a reporter group, which enables the target protein(s) to be detected and/or isolated.^24^

Here, we use live-cell PAL to demonstrate that **ARN23765** binds directly to CFTR, in situ. Using in silico analyses and in cell studies we describe **ARN23765**’s mode of action and putative binding site, unveiling the amino acids essential for the F508del-CFTR rescue.

## Results

### Synthesis and Biological Activity of ARN23765-Derived Photoaffinity Probes

As the most suitable photoreactive moiety for the design of the photoaffinity probes to be used in live-cell PAL experiments, we favored the diazirine ring because of its small steric hindrance, assuming that it would only marginally affect the probes’ biological activity.^25,26^ Likewise, we chose to insert a terminal triple bond (i.e., an alkyne) as an appropriate functionality for a two-step conjugation via copper-catalyzed azide–alkyne cycloaddition (CuAAC)^27,28^ with a desthio-biotin (DS-biot) as a purification tag. Furthermore, to simplify the PAL experimental strategy, we also synthesized a set of **ARN23765**-derived PAPs bearing a preinstalled DS-biot, which could prevent the application of the CuAAC protocol.

**Scheme 1:**
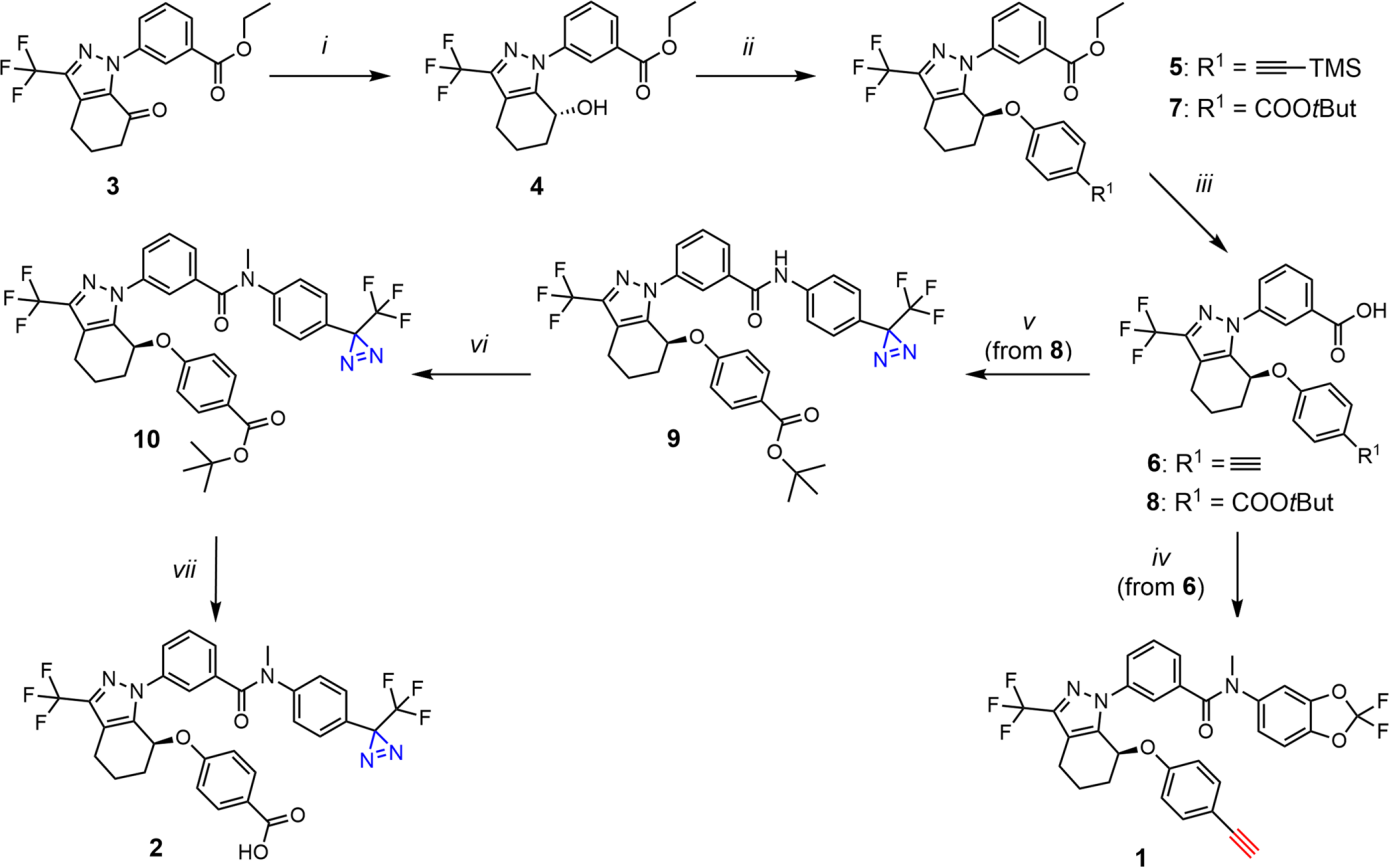
Synthesis of ARN23765’s Close Analogues (1 and 2) *Reagents and conditions: (i)* Formic acid, RuCl(p-cymene)[(*R,R*)-Ts-DPEN], 2-propanol, TEA, r.t., 18 h, 93%; *(ii) tert*-butyl 4-hydroxybenzoate or 4-((trimethylsilyl)ethynyl)phenol,^30^ PMe_3_ (1.0 M in THF), DIAD, THF, 0 °C to r.t., 18 h, 66–76%; *(iii)* for **5**: LiOH (1.1 M in H_2_O), THF/MeOH, r.t., 18 h,, then LiOH (1.1 M in H_2_O), THF, r.t., 18 h, *quant.*; for **7**: LiOH (0.88 M in H_2_O), THF, r.t., 19 h, *quant.* ; *(iv)* 2,2-difluoro-*N*-methylbenzo[*d*][1,3]dioxol-5-amine hydrochloride, T_3_P (50% in AcOEt), DIPEA, AcOEt, 0 °C to r.t., 18 h, 88%; *(v)* (a) trichloroacetonitrile, PPh_3_, DCM, 0 °C to r.t., 1.5 h, (b) 4-[3-(trifluoromethyl)-3*H*-diazirin-3-yl]aniline hydrochloride, TEA, DCM, 0 °C to r.t., 1.5 h, 68%; (*vi*) CH_3_I, Cs_2_CO_3_, DMF, 0 °C to r.t., 18 h, 94%; (*vii*) TFA, DCM, 0 °C to r.t., 4 h, 56%.

We initially investigated the impact of key structural modifications on **ARN23765** activity by devising and synthesizing two close analogues. Analogue **1** was designed featuring a terminal alkyne instead of the carboxylic acid group, while analogue **2** presented a trifluoromethyl-phenyl diazirine motif in place of the *gem*-difluorobenzodioxole moiety. Starting from tetrahydro-1*H*-indazol-yl benzoate (**3**),^29^ as a key-intermediate, the two compounds (**1** and **2**) were obtained after a sequence of conventional synthetic steps (Scheme 1, Figure S1 and the Experimental Section for the detailed synthetic protocols). To note, while the hydrolysis of the ester **7** in alkaline medium (LiOH) furnished the desired carboxylic acid **8**, the reaction of ethyl ester **5** in a THF/MeOH/H_2_O system led to the removal of the trimethylsilyl (TMS) group and methanolysis of the ethyl ester, which upon treatment with aqueous LiOH in THF was converted into the corresponding carboxylic acid **6**.

These analogues were tested in the functional assay on the human bronchial epithelia-derived cell line, CFBE41o-, which stably overexpresses F508del-CFTR and the halide-sensitive yellow fluorescent protein (HS-YFP).^31^ Interestingly, **1** almost completely retained the activity (EC_50_ = 3.3 nM; *E*_max_ = 3.0) of its parent compound (EC_50_ < 3 nM; *E*_max_ = 2.7), whereas **2** showed a markedly reduced potency and efficacy (EC_50_ = 24 nM; *E*_max_ = 1.2), highlighting the importance of the *gem*-difluorodioxole moiety for **ARN23765**-induced CFTR correction (Figure S2).

**Scheme 2:**
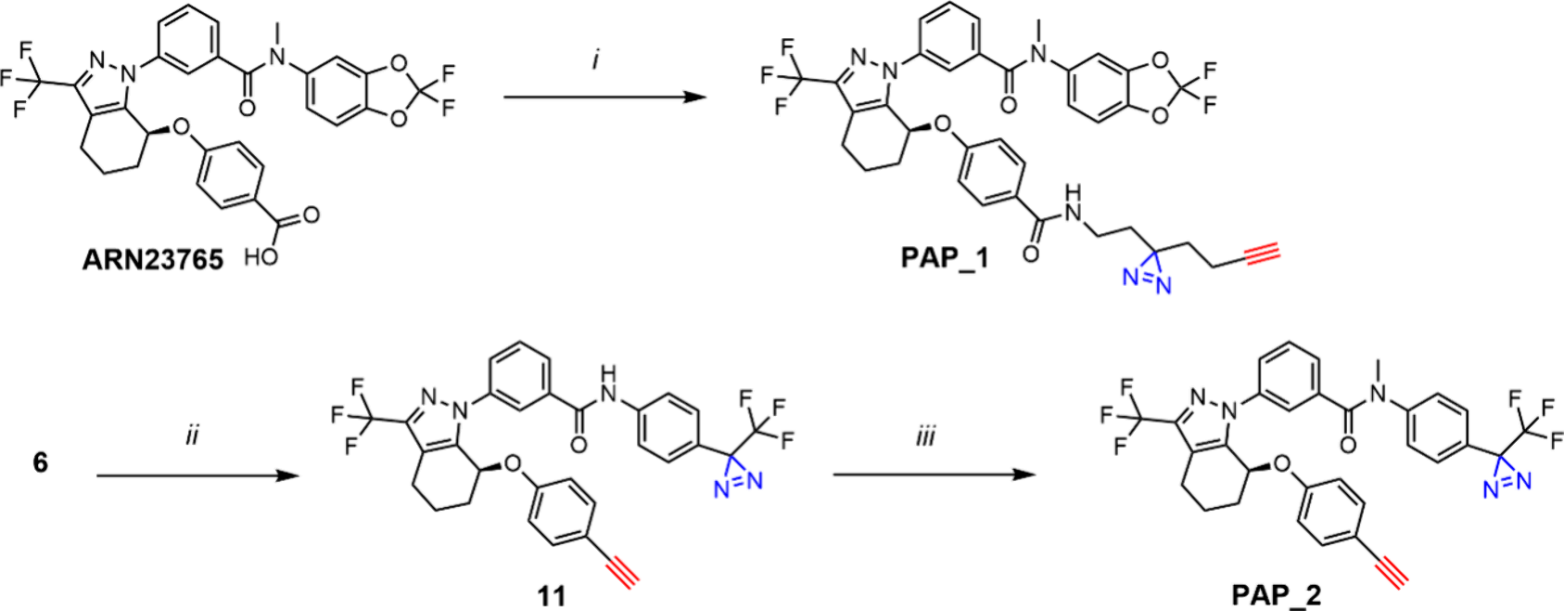
Synthesis of ARN23765-Derived Alkyne-Substituted Probes (PAP_1 and PAP_2) *Reagents and conditions*: (*i*) 2-(3-(But-3-yn-1-yl)-3*H*-diazirin-3-yl)ethan-1-amine,^34^ HATU, DIPEA, DMF, 0 °C to r.t., 18 h, 84%; *(ii)* (a) trichloroacetonitrile, PPh_3_, DCM, 0 °C to r.t., 2 h, (b) 4-[3-(trifluoromethyl)-3*H*-diazirin-3-yl]aniline hydrochloride, TEA, DCM, 0 °C to r.t., 1.5 h, 80%; (*iii*) CH_3_I, Cs_2_CO_3_, DMF, 0 °C to r.t., 2.5 h, 80%.

Based on these findings, we designed and synthesized the enantiomerically pure photoaffinity probe **PAP_1**, which featured a “minimalist” alkyl diazirine-containing linker bearing a terminal alkyne bound to the benzoic moiety of the parent compound via an amide bond.^32^ Despite compound **2** has no significant effect in CFTR rescuing (Figure S2), we also explored **PAP_2**, which was synthesized via a nested strategy^33^ by introducing the diazirine and alkyne handle in different positions of **ARN23765**’s core structure, i.e. in place of the *gem*-difluorodioxole moiety and the carboxylic acid group, respectively. While **PAP_1** was smoothly synthesized from **ARN23765** via a simple amide bond formation, **PAP_2** was afforded following a protocol combining two straightforward synthetic steps which led to analogues **1** and **2** (Scheme 2, Figure S1 and the Experimental Section for the detailed synthetic protocols).

When tested in the HS-YFP functional assay in F508del-CFTR CFBE41o- cells, **PAP_1** showed a comparable potency (EC_50_ < 3 nM; *E*_max_ = 2.8) to **ARN23765**, while **PAP_2** displayed a lower but still detectable activity (EC_50_ = 385 nM; *E*_max_ = 2.1) (Figure S2).

**Scheme 3:**
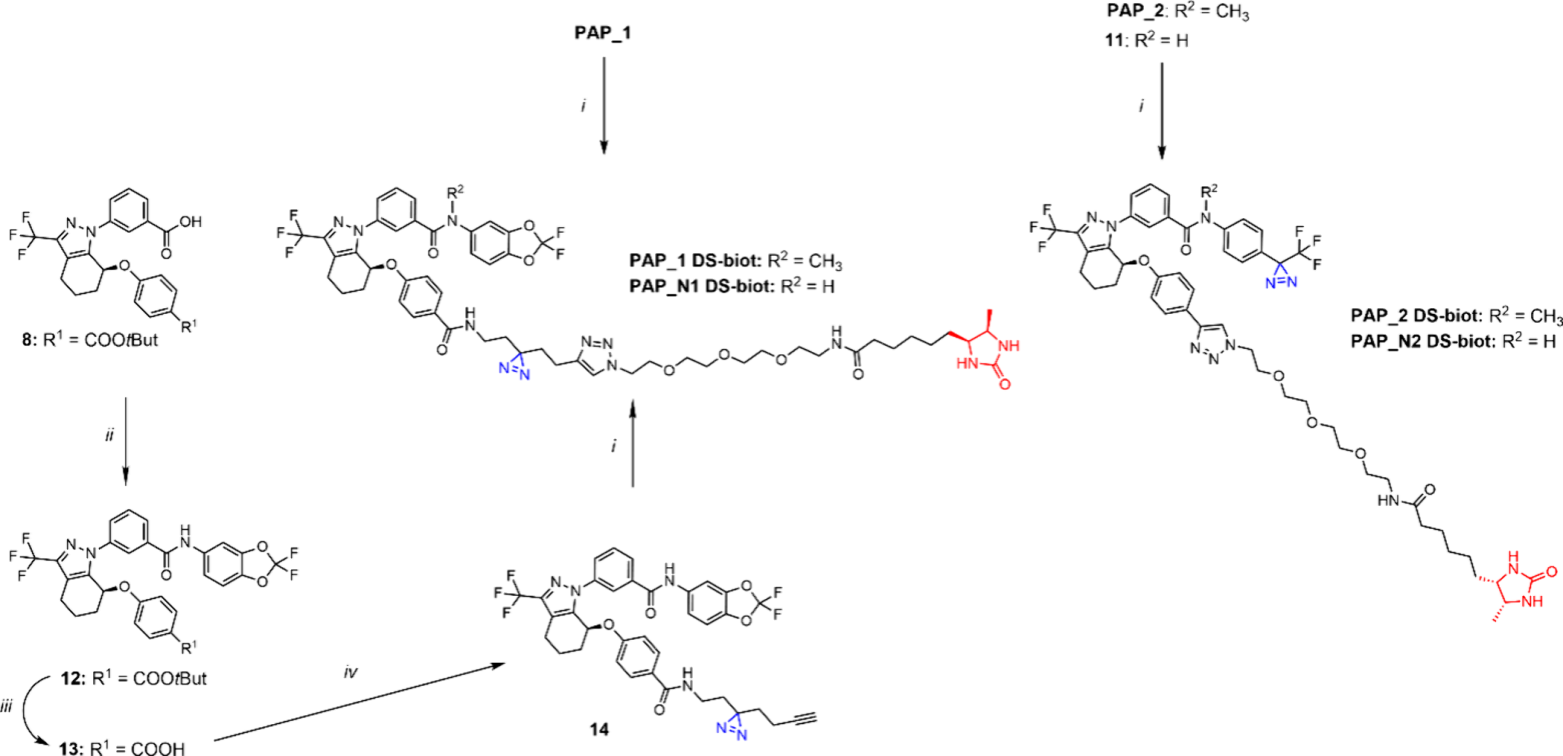
Synthesis of ARN23765-Derived Desthiobiotinylated Probes (PAP_1 DS-biot, PAP_N1 DS-biot, PAP_2 DS-biot, and PAP_N2 DS-biot) *Reagents and conditions*: (*i*) Desthiobiotin-PEG3-azide, CuSO_4_·5H_2_O, sodium ascorbate, THF, H_2_O/*t*-ButOH, r.t., 18 h, 30–60%; (*ii*) 2,2-difluoro-5-aminobenzodioxole, HATU, DIPEA, DMF, 0 °C to r.t., 18 h, 85%; *(iii)* TFA, DCM, 0 °C to r.t., 3 h, *quant*.; (*iv*) 2-(3-(but-3-yn-1-yl)-3*H*-diazirin-3-yl)ethan-1-amine,^34^ HATU, DIPEA, DMF, 0 °C to r.t., 18 h, 56%.

To synthesize **ARN23765**-derived PAPs bearing a preinstalled DS-biot, the terminal alkyne group of **PAP_1** and **PAP_2** was exploited in a CuAAC reaction in the presence of desthiobiotin-PEG3-azide to produce **PAP_1 DS-biot** and **PAP_2 DS-biot**. We also synthesized **PAP_N1 DS-biot** and **PAP_N2 DS-biot**, featuring a secondary amide moiety, known to be detrimental to CFTR correction, as negative controls for **PAP_1-** and **PAP_2 DS-biot**, respectively (Scheme 3, Figure S1 and the Experimental Section for the detailed synthetic protocols). **PAP_1 DS-biot** and **PAP_2 DS-biot** activity (EC_50_ = 122 and 480 nM; *E*_max_ = 2.7 and 1.4, respectively) was in the range observed for the corresponding alkyne probes, whereas **PAP_N1 DS-biot** and **PAP_N2 DS-biot** were devoid of activity, as expected (Figure S2).

### PAL Studies in Live Cells

To perform PAL experiments with the alkyne-substituted probes, CFBE41o- cells overexpressing wild-type (wt)-CFTR were incubated with **PAP_1** or **PAP_2** and exposed to UV irradiation to enable probe/protein cross-linking. After cell lysis, a DS-biot-tag was inserted via CuAAC. PAP-bound proteins were enriched on streptavidin beads (pull-down) and the presence of CFTR was analyzed with Western blot (WB). After several rounds of optimization, we could barely detect a band corresponding to CFTR in **PAP_2**-incubated cells (Figure S3A). We observed that CFTR content in cell lysates was dramatically reduced upon CuAAC implementation, likely hampering its recovery during pull-down experiments (Figure S3B).

**Figure 2:**
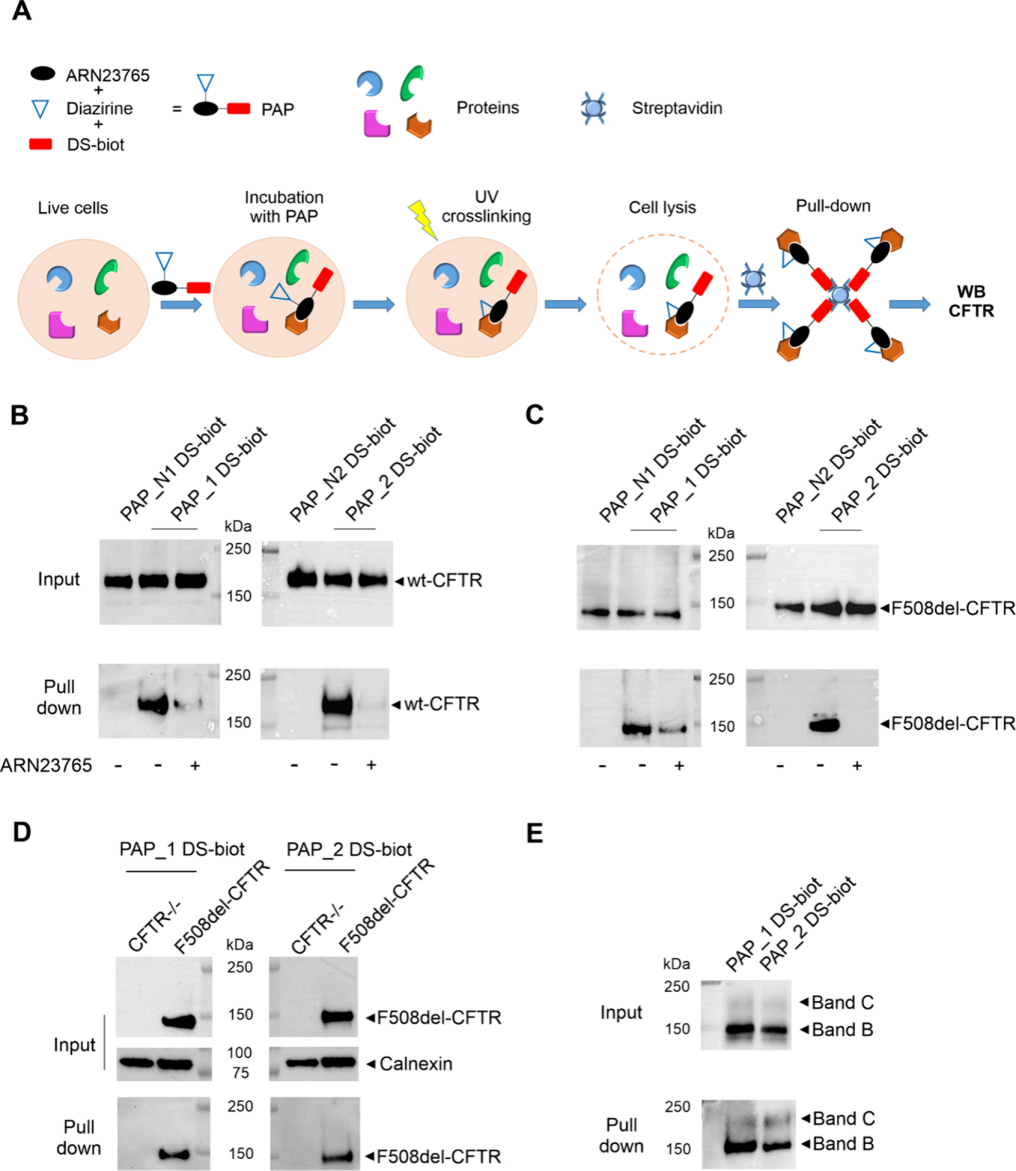
PAL experiments with DS-biotinylated PAPs in wt- and F508del-CFTR CFBE41o- cells. (A) Schematic representation of live-cell photoaffinity labeling (PAL) protocol with biotinylated PAPs. (B–C) Western blot analyses of CFTR expression in streptavidin pulled-down proteins from PAL experiments in wt- (B) or F508del-CFTR- (C) CFBE41o- cells incubated for 2 h with **PAP_1 DS-biot** (1 μM) or **PAP_2 DS-biot** (1 μM). Cells were either preincubated or not with **ARN23765** (25-fold excess). **PAP_N1 DS-biot** and **PAP_N2 DS-biot** were used as negative controls for **PAP_1 DS-biot** and **PAP_2 DS-biot**, respectively. Arrows indicate the band corresponding to wt- or F508del-CFTR. (D) Western blot analysis of CFTR expression in streptavidin pulled-down proteins from PAL experiments in CFTR^–/–^ CFBE41o- cells incubated for 2 h with **PAP_1 DS-biot** (1 μM) or **PAP_2 DS-biot** (1 μM). F508del-CFTR CFBE41o- cells were used in parallel as pull-down positive control. Calnexin was detected as loading control. (E) F508del-CFTR CFBE41o- cells were kept at 27 °C for 24 h to induce F508del-CFTR maturation before PAL experiments with **PAP_1 DS-biot** and **PAP_2 DS-biot**. Arrows indicate immature protein (band B) and fully glycosylated mature protein (band C). In the upper blots of each panel, input lanes represent probe-incubated samples before pull-down. The molecular weight marker is indicated in kilo Dalton (kDa). The images reported in panels B and C are representative of three independent experiments, while experiments in panels D and E were performed in duplicates. Quantifications of the competition of PAPs with an excess of **ARN23765** (panel B and C) are shown in Figure S4.

The probes with a DS-biotin moiety preinstalled in their structure (**PAP_1 DS-biot** and **PAP_2 DS-biot**) allowed a simplified PAL procedure, i.e. avoiding the CuAAC protocol (Figure 2A). The new protocol helped preserving CFTR in the experimental samples. Indeed, both wt- and F508del-CFTR were detected in input lysates from the corresponding overexpressing CFBE41o- cells treated with either **PAP_1 DS-biot** or **PAP_2 DS-biot** (Figure 2B–C, upper blots). Most importantly, we consistently captured the proteins in pull-down experiments (Figure 2B–C, lower blots). In addition, competition experiments, where cells were preincubated with an excess of **ARN23765**, resulted in a markedly reduced amount of the recovered protein (Figure 2B–C and Figure S4). This demonstrates that the binding of these PAPs to CFTR was specific and provides indirect evidence for the binding of **ARN23765** to CFTR in cells.

As expected, CFTR was not pulled down from wt- or F508del-CFTR CFBE41o- cells incubated with **PAP_N1-** or **PAP_N2 DS-biot** (Figure 2B–C). Moreover, we did not detect CFTR in PAL experiments in CFTR^–/–^ CFBE41o- cells incubated with **PAP_1-** or **PAP_2 DS-biot** (Figure 2D). Interestingly, **PAP_1 DS-biot** and **PAP_2 DS-biot** bound and pulled down both F508del-CFTR immature (band B) and mature (band C) protein after low-temperature rescue, overall validating our **ARN23765**-derived probes as a tool for studying CFTR (Figure 2E).

### ARN23765 Interaction with CFTR Domains

**Figure 3:**
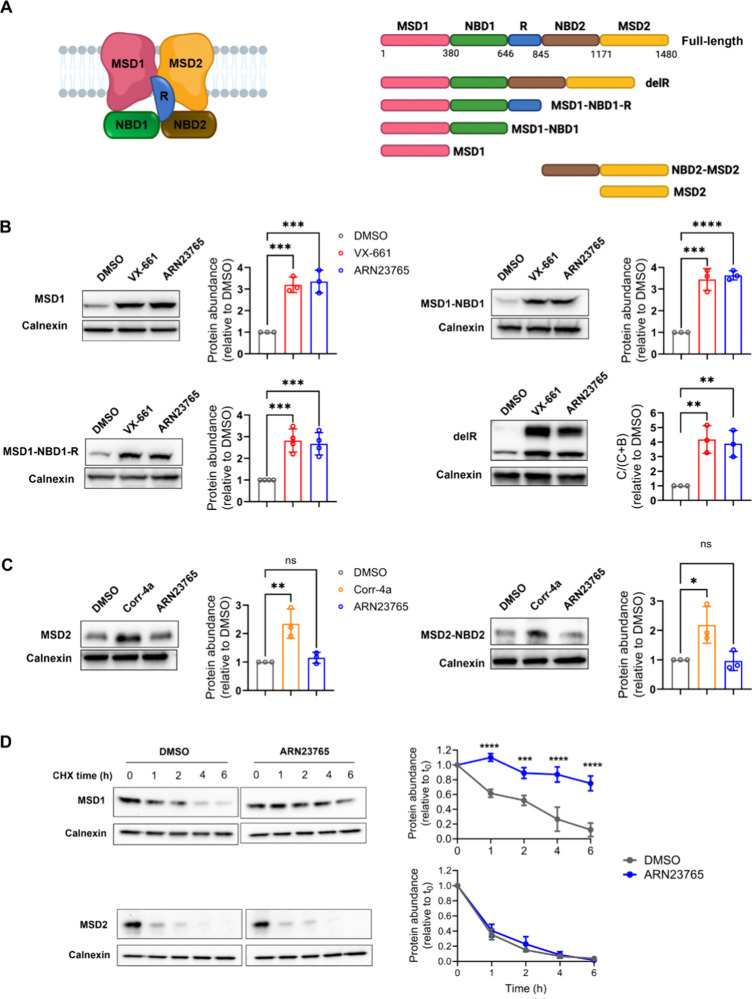
**ARN23765** binds to CFTR-MSD1. (A) Schematic representations of CFTR structure (*left*) and constructs coding for the analyzed CFTR fragments (*right*). (B–C) **ARN23765**’s effect on the abundance of CFTR fragments: HEK293 cells were transfected with constructs coding for CFTR fragments containing MSD1 (B) or MSD2 (C), and incubated for 24 h with **ARN23765** (10 nM). VX-661 (3 μM) and corr-4a (10 μM) were used as positive controls for MSD1- and MSD2-containing fragments, respectively. DMSO (0.2%) was used as negative control. Protein expression was analyzed by WB. On the right of each representative WB, bar graphs report the quantification data (mean ± SD, 3 independent experiments) of CFTR fragment intensity normalized to calnexin and expressed as a ratio of DMSO control. For the delR construct, the protein maturation was quantified using the formula [band-C/band C+B] and expressed as a ratio of DMSO control. One-way ANOVA (Dunnett’s multiple comparisons test, DMSO vs corrector: * *p* < 0.05, ** *p* < 0.01; *** *p* < 0.001, **** *p* < 0.0001). (D) Cycloheximide chase experiments: HEK293 cells were transfected with MSD1 or MSD2 and, after 24 h incubation with **ARN23765** (10 nM) or DMSO (0.2%), protein synthesis was inhibited with the addition of cycloheximide (CHX, 200 μM). *Left*: representative WB analysis of CFTR MSD1 and MSD2 expression at the indicated time points (h). *Right*: quantification (mean ± SD, 3 independent experiments) of the intensity of CFTR domains normalized to calnexin and expressed relative to time 0 (t_0_). Two-way ANOVA (Bonferroni’s multiple comparison test, DMSO vs **ARN23765**: *** *p* < 0.001, **** *p* < 0.0001).

CFTR correctors are expected to stabilize the expression of the protein domains with which they interact, resulting in an increased amount of protein.^35−37^ To determinate the portion of CFTR with which **ARN23765** interacts, constructs coding for CFTR fragments of different lengths (Figure 3A) were transfected in HEK293 cells, and the effect of 24 h incubation with **ARN23765** on the expression of the truncated proteins was analyzed with WB. When cells were incubated with **ARN23765**, there was an increased expression of CFTR fragments containing MSD1 (Figure 3B), but no difference in expression of CFTR fragments containing MSD2 (Figure 3C). In addition, when the R domain was deleted, the protein was still corrected by **ARN23765**, indicating that the R domain was not necessary for binding (Figure 3B). To better study **ARN23765**’s stabilization of MSD1 vs MSD2, we analyzed their expression in transfected HEK-293 cells over 6 h of cycloheximide treatment. Figure 3D shows that **ARN23765** increased MSD1’s half-life, while displaying no effect on MSD2’s stability.

**Figure 4:**
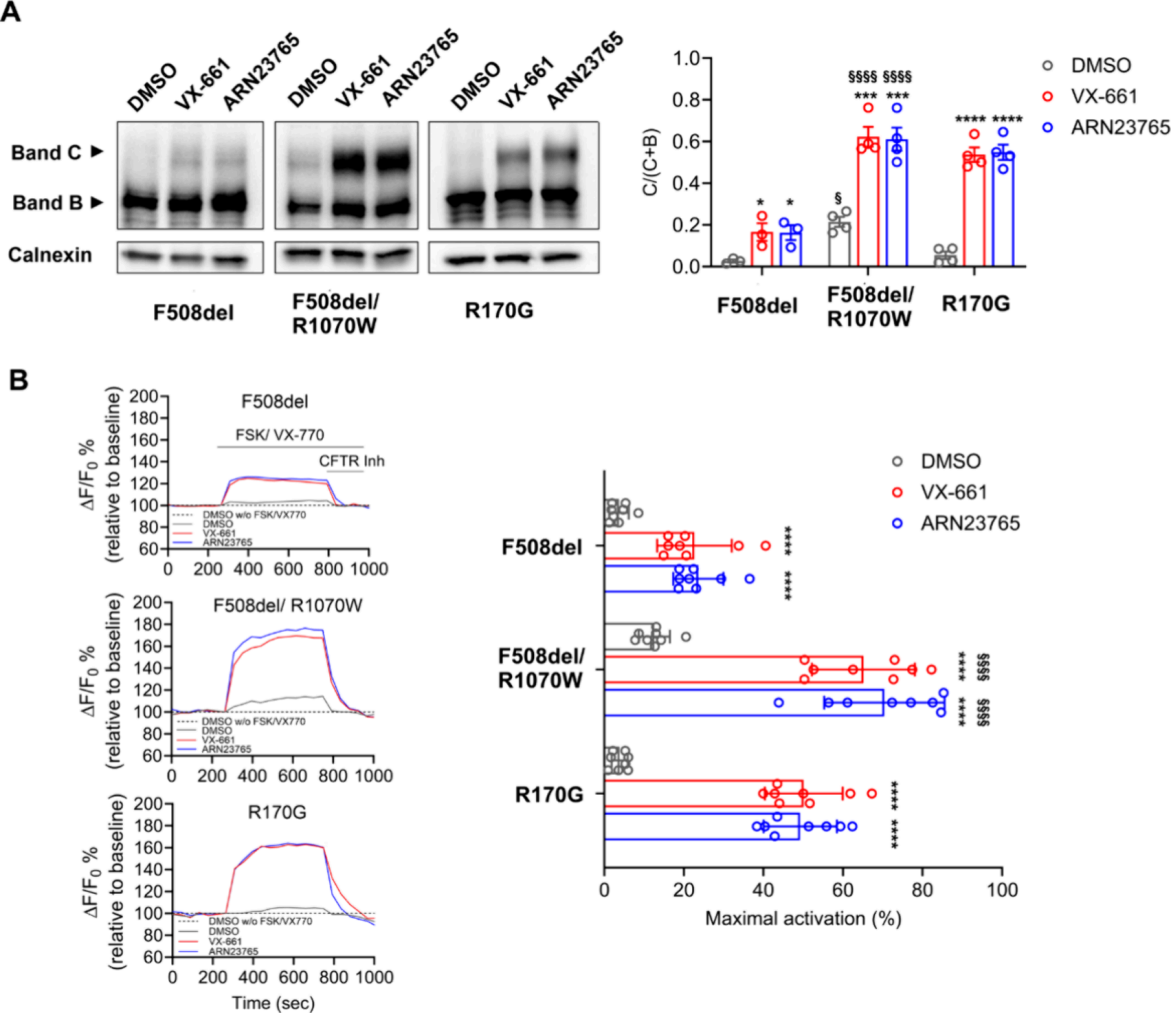
**ARN23765** stabilizes NBD1-ICL4 and NBD1-ICL1 interactions. HEK293 cells were transfected with the constructs coding for F508del-CFTR, F508del/R1070W-CFTR, and R170G-CFTR and incubated for 24 h with **ARN23765** (10 nM) or VX*-*661 (3 μM). DMSO (0.2%) was used as negative control. (A) *Left:* representative WBs with detection of CFTR B- and C-bands. *Right*: quantification (mean ± SD, 3–4 independent experiments) of CFTR band-C abundance, calculated using the conventional equation [band-C/band C+B]. (B) CFTR channel activity was evaluated with fluorometric imaging plate reader functional assay (*FLIPR*).^40^ Plasma membrane depolarization was detected as an increase in fluorescence (ΔF), after which CFTR was inhibited by the addition of inhibitor-172, (10 μM). *Left*: representative fluorescence traces. *Right*: peak change in fluorescence (mean ± SD, 8 replicates), expressed relative to baseline fluorescence (ΔF/F_0_). FSK: forskolin; CFTR Inh: inhibitor-172. Statistical analysis in (A,B): One-way ANOVA (Dunnett’s multiple comparisons test, DMSO vs corrector: * *p* < 0.05, *** *p* < 0.001, **** *p* < 0.0001); Two-way ANOVA (Tukey’s multiple comparison test, F508del-CFTR vs F508del/R1070W-CFTR: ^§^*p* < 0.05, ^§§§§^*p* < 0.0001).

We further investigated the function of **ARN23765** on NBD1-ICL4 and NBD1-ICL1 interfaces of CFTR. The second-site mutation R1070W is known to stabilize the NBD1-ICL4 interface, which is normally disrupted in F508del-CFTR protein.^38^ Incubation of cells with **ARN23765** or VX-661 further enhanced both F508del/R1070W protein maturation (Figure 4A) and channel activity (Figure 4B) in transfected HEK293 cells, similar to what has been described for VX-809.^12^ We then explored **ARN23765**’s effect on the ICL1-NBD1 interface. It has previously been demonstrated that the CF-causing R170G mutation in ICL1 impairs the processing of wt-CFTR.^12,39^ Interestingly, **ARN23765** rescued this mutation, improving maturation (Figure 4A) and activity (Figure 4B) of R170G-CFTR in transfected HEK293 cells. Taken together, these studies indicate that **ARN23765** stabilizes MSD1 and improves the assembly of F508del-CFTR by rescuing the intradomain interfaces.

### Molecular Docking Calculations of ARN23765 Binding to F508del-CFTR

**Figure 5:**
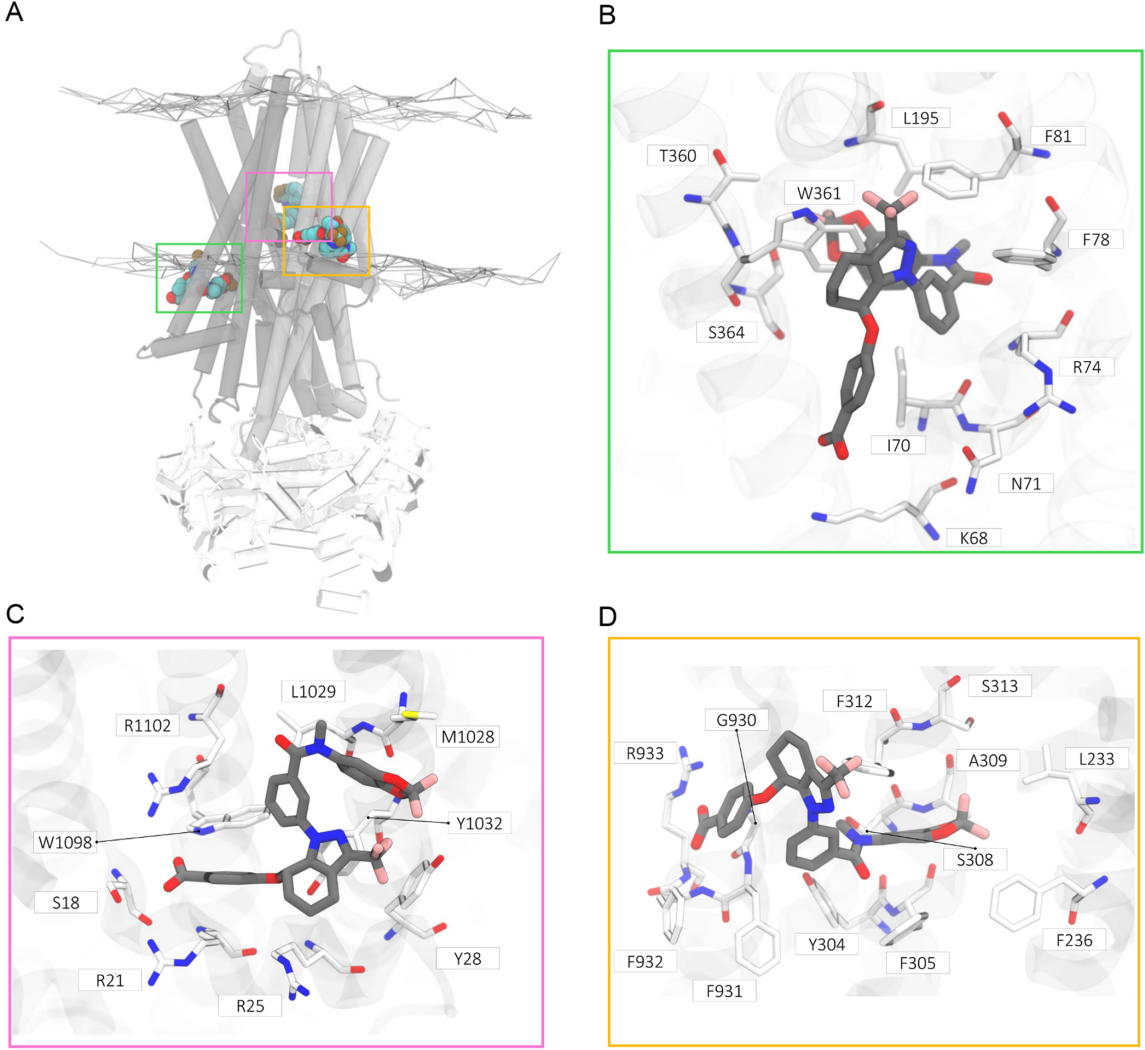
Molecular docking binding mode predictions in the described CFTR binding sites.^18^ A) Cartoon representation of F508del-CFTR embedding **ARN23765** in the analyzed binding sites (type I: green box; type III: pink box; potentiator: orange box). (B-D) Stick representations of calculated binding mode for **ARN23765** in the binding sites of type I (B), type III (C) corrector, and potentiator (D).

To verify whether **ARN23765** interacted preferentially with one or more of the binding sites of approved modulators, molecular modeling studies were conducted on the latest released PDB structures of F508del-CFTR bound to type I (VX-809) and type III (VX-445) correctors, and the potentiator ivacaftor (VX-770) (PDB-ID: 8EIO, 8EIG, 8EIQ, respectively)^18^ (Figure 5A). The best-scoring pose from the docking calculations of **ARN23765** demonstrated similar interactions to the type I corrector binding mode (Table S1 and Figure S5).^19^ The *gem*-difluorobenzodioxole group was wrapped by a few lipophilic residues (I368, L365, S364, W361, T360, L195, F78, F81, A198, I70), perfectly fitting the same hydrophobic pocket. The cyclopropane group of VX-809 is switched out in **ARN23765** for a tertiary methyl-amide moiety, which extends outside the cavity, and together with the phenyl ring acts as a linker to the polar end of the molecule. This latter group interacts with tryptophan in position 361 (W361) through a π–π displaced stacking interaction. Notably, the indole ring appears to play a crucial role in creating a sandwich-like interaction where the aromatic rings of the *gem*-difluorobenzodioxole and the tetrahydro-indazole groups of **ARN23765** embrace the residue side chain, resulting in ligand stabilization. Lastly, the *para*-carboxy-substituted phenyl ring of **ARN23765** extends downward to the cytoplasmic bulk, where the acidic functionality interacts weakly with R74 and with the proximal K68 through salt bridges (Figure 5B, and S5A).

**Figure 6:**
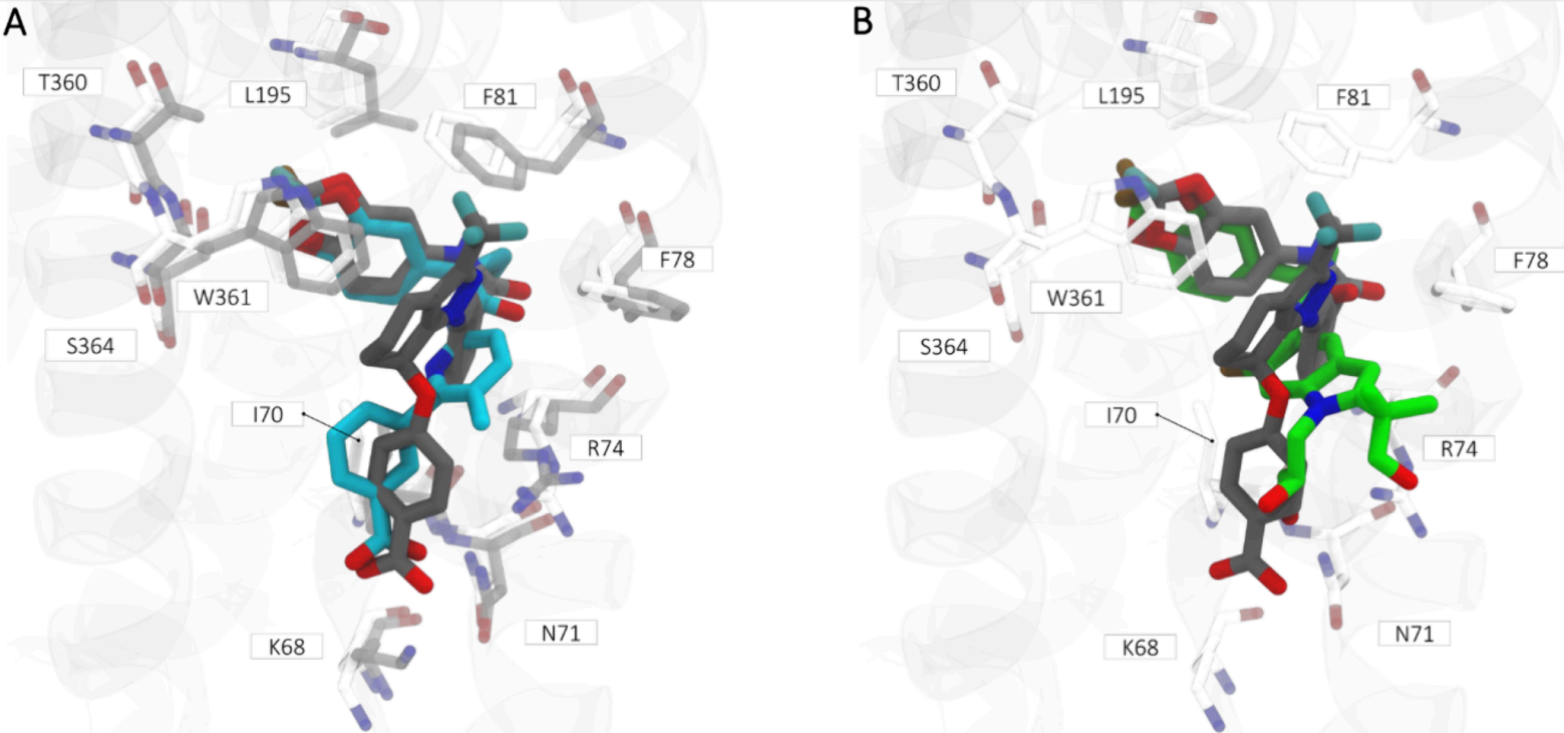
Structural comparison between cryo-EM structures of VX-809 and VX-661^19^ and **ARN23765** docking pose. A) Overlay between **ARN23765** docking pose (black bold sticks) and VX-809 (cyan bold sticks). The pocket conformation of the two structural residues (PDB-ID: 8EIQ and PDB-ID:8EIO) are represented in white and gray sticks, respectively. B) Overlay between **ARN23765** docking pose (black bold sticks) and VX-661 (green bold sticks; PDB-ID: 8EIQ).

The docking mode for **ARN23765** aligned with reported type I correctors (i.e., VX-809/VX-661) cryo-EM structures^19^ shares similar interactions within the cavity’s lipophilic region. The binding site’s most notable differences appears in its polar regions, where lysine (K68) and arginine (R74) residues adopted different conformations. While **ARN23765** maintains with its benzoic acid functionality a key salt bridge interaction with K68, as for VX-809 (Figure 6A), this interaction is absent with the vicinal dihydroxy moiety of VX-661 (Figure 6B). Moreover, unlike **ARN23765**, both correctors VX-809 and VX-661 establish π-cation interaction with the side chain of R74 in the binding pocket. Notably, **ARN23765** compensates for this missing interaction by forming a pivotal double π–π stacking with W361 residue.

To compare scores and binding modes and to validate results, we performed similar computations for **ARN23765** on the type III corrector (VX-445) and potentiator (VX-770) binding sites (Table S1). Previously reported computational analyses of the VX-445 binding site indicated that most of elexacaftor’s molecular scaffold occupies a hydrophobic crevice proximal to transmembrane (TM) helices 2 and 11 (residues I132, V1108, I1109, M1105). The drug’s corrective action may be due to the presence of charged residues like R1102 and R21 in the TM helix 11 and lasso motif, respectively.^18,41^ According to docking calculations, TM helices 2 and 11 were not essential for the interactions with **ARN23765**. The compound interacted via two salt bridges with only the positively charged R1102 and R21 residues and with one hydrogen bond involving the carboxy-terminus with W1098. The rest of **ARN23765**’s structure is right-shifted toward the TM helix 10 surface, where only Y1032 engages in a stable π-stacking interaction (Figure 5C and S5B).

Finally, **ARN23765** was docked in VX-770 binding site,^42,43^ which has been described on the protein/membrane interface, where TM-4, TM-5 and TM-8 helices contribute to creating a pocket, and MSD1 and MSD2 construct a small nesting groove for the CFTR modulator. **ARN23765** binds to the reported potentiator binding site only via its carboxyl and *gem*-difluorobenzodioxole moieties. The molecule’s core is driven toward the membrane by the lipophilic trifluoromethyl-tetrahydroindazole ring, while the carboxyl functionality directs the ligand to form a salt bridge with R933 and F932 backbone. While F236 and F305 bind the *gem*-difluorobenzodioxole group that is resting in the binding site’s right-hand crevice, F312 results in being stuck to the aromatic central core of the molecule (Figure 5D and S5C).

Due to the importance of the *gem*-difluorobenzodioxole moiety for stabilizing **ARN23765** within the type I corrector binding site, we investigated whether the structural modifications introduced in analogue **2** and **PAP_2/PAP_2 DS-biot** could impair the corresponding interaction within the binding site. We conducted computational docking analyses on analogue **2**, as a representative compound lacking this functionality (Figure S6A). In contrast to **ARN23765**, compound **2** displayed less affinity for the type I corrector binding site, while showing a similar binding mode toward the potentiator binding site (Figure S6B-D). Indeed, in type I corrector binding site, **2** is outward shifted to the binding pocket, linked by a series of alternates salt bridges with positively charged residues (R74, R78), but not strong enough to retain the molecule in its original conformation (Figure S6B). The lower value of docking score for **2**, in corrector type I binding pocket, is in agreement with its reduced activity as a CFTR corrector (Table S1).

The compounds docking scores across all three binding sites were complemented with binding free energy (Δ*G*bind) calculations for **ARN23765** and compound **2**. The analysis confirms **ARN23765**’s efficacy as a type I corrector, with nonbonded interaction energies (including Coulombic, lipophilic, packing, and van der Waals) showing similar magnitudes to those of VX-809 (Table S2).

### Molecular Dynamics Simulations of ARN23765 Binding to F508del-CFTR

**Figure 7:**
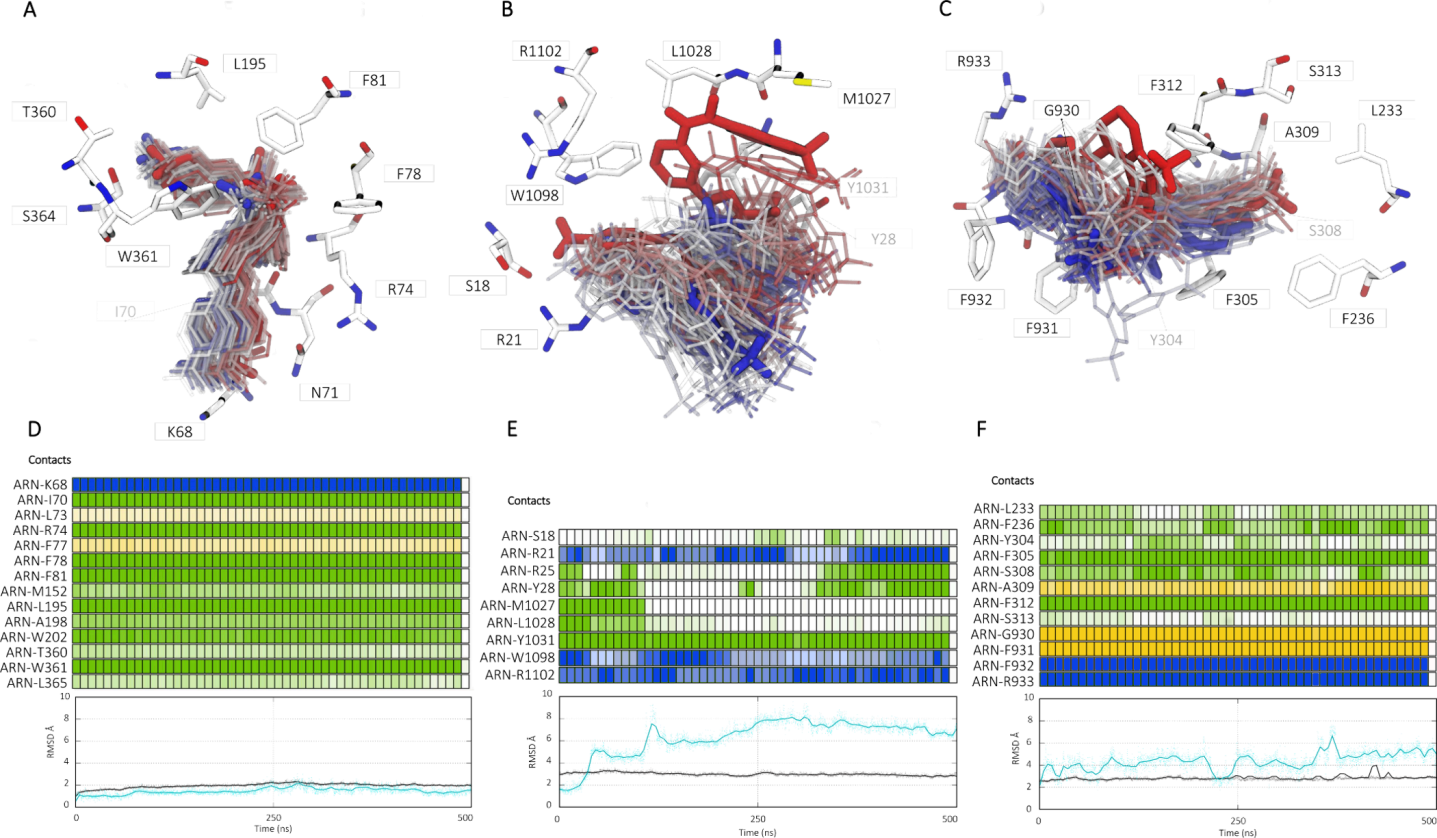
Molecular dynamics (MD) simulations contact analyses of **ARN23765** in VX-809, VX-445, and VX-770 binding cavities. A-C) Pictures represent the molecule conformational variability during the 500 ns long simulations. Red to blue color transitions stand for the time variation of the ligand in its cavity, from the time 0 to the nanosecond 500th. (D-F) MD time frame contact and root-mean-square-deviation (RMSD)^44^ analyses of **ARN23765** in the binding sites of type I (D), type III (E) corrector, and potentiator (F). *On the top:* time frame evolution of **ARN23765** during 0.5 μs of MD simulation, in which colored boxes represent the interactions between the ligand and the amino acid residues via H-bond (blue), side chain apolar (green) and backbone (yellow). The contact cutoff between **ARN23765** and the residues was set at 2.5 Å and the color intensity is proportional to the contact’s distance. White boxes represent lost interactions, i.e. over 6 Å. *On the bottom*: RMSD analysis for F508del-CFTR (black trace) and **ARN23765** (cyan trace) during the entire run. The protein conformational stability was calculated by measuring the atomic coordinates average displacement between each time step and a reference structure. In general, the smaller the deviation, the more stable is the protein structure. The equilibrated F508del-CFTR was used as a reference. The analysis was also performed to measure the ligand stability in each binding pocket, using the docking pose as reference.

To refine the binding modes observed in the docking procedure, we applied classical Molecular Dynamics (MD) simulations to all complexes involving the poses of **ARN23765** in the three effector pockets of F508del-CFTR (Figure 7A-C). By monitoring the stability of the binding mode over time, it is possible to identify unreliable docking results. A meaningful docking pose is expected to display stable and specific interactions with the target, showing a low root-mean-square deviation (RMSD)^44^ over time with respect to the starting configuration. As shown in Figure 7D-F, all investigated complexes achieved a stable protein conformation during the simulation time courses (RMSD black traces for F508del-CFTR reached convergence, maintaining on average a fixed value during the 500 ns simulation). Notably, **ARN23765** interacted differently depending on the binding site.

In type I corrector binding site, the compound tended to preserve its original conformation, as demonstrated by the stable RMSD trend (Figure 7D, bottom). The interaction pattern also remained consistent during the simulation time, with the preservation of both the salt bridge between the carboxy group and K68, and the double π–π stacking of the aromatic rings and W361 (Figure 7D, top). In the early stages of the simulation in the type III corrector binding site, **ARN23765** showed a different pattern, with large fluctuations within the binding pocket, but maintenance of some interactions from the docking pose. In general, the RMSD suggests poor ligand stability, jumping up to 9 Å (Figure 7E, bottom). The ionic interaction with R21 was transient from the initial time course, in favor of a long lasting and more stable interaction toward R1102. In addition, together with the H-bond charge enforced to W1098, the polar moiety of the ligand was strongly linked to those residues located on the TM helix 11. The tetrahydroindazole moiety and the *gem*-difluorobenzodioxole group were those most affected by the overall conformational changes of **ARN23765**, maintaining only the π–π interaction to Y1032 (Figure 7E, top).

For the simulation of **ARN23765** in the potentiator binding site, the ligand RMSD fluctuated up to 6 Å, demonstrating some changes from the predicted docking pose (Figure 7F, bottom). Again, the polar moiety showed the strongest and most persistent interactions with a positively charged residue (R933) and the backbone nitrogen of F932, to which the H-bond was maintained during the entire run. The molecule tended to vary its original conformation, adopting a stretched conformation and showing new π–π interactions with the peripheral phenylalanine residues (F229 and F236, respectively) (Figure 7E, top).

### In Silico Single-Point Mutation Analysis within the CFTR Binding Site for ARN23765

**Figure 8:**
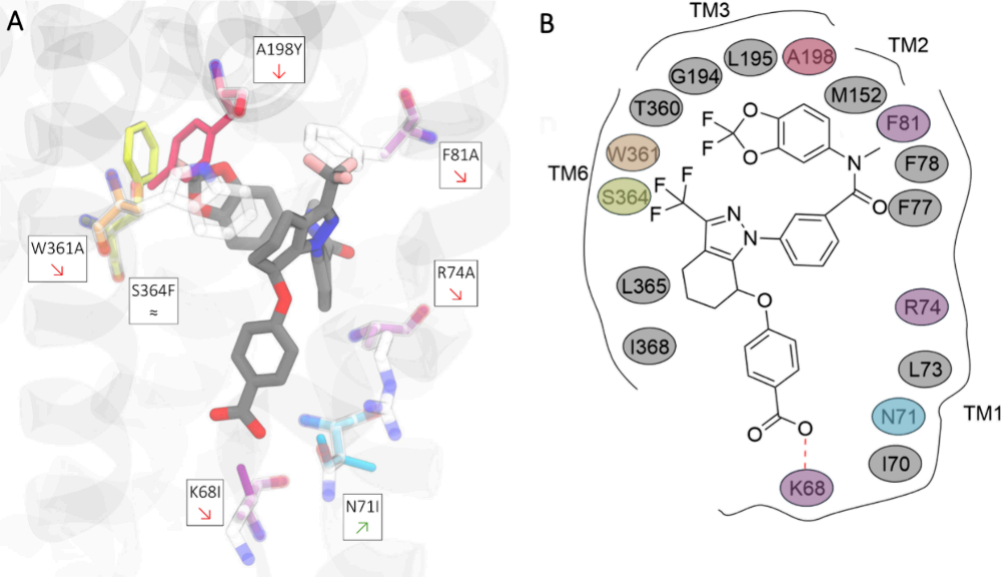
In silico single-point mutation analysis within the CFTR binding site for **ARN23765**. (A) Cartoon representing **ARN23765** docked in the type I corrector binding site; the original residues are shown in transparency, while the mutations are bold colored, consistent with Table 1; red and green arrows inside the residue name label depict the mutation’s effect. (B) 2D-Ligand interaction diagram of **ARN23765** into F508del-CFTR corrector type I binding site. The amino acids selected for mutations are colored consistent with Table 1.

To validate the amino acid interactions predicted by the docking calculations, we produced in silico single-point mutations of the type I corrector binding site residues involved in the ligand binding. Starting from the most relevant region within this binding site,^19^ the original residues were mutated from the inner cavity to the membrane-exposed interface (Figure 8) and the Δaffinity parameter^45^ was determined to assess the impact of each mutation (Table 1). The Δaffinity parameter measures how mutations affect ligand-protein binding strength. Higher values indicate the mutation weakens binding, while negative values indicate that the compound binds better to the mutant than the parent protein.^45^

**Table 1:**
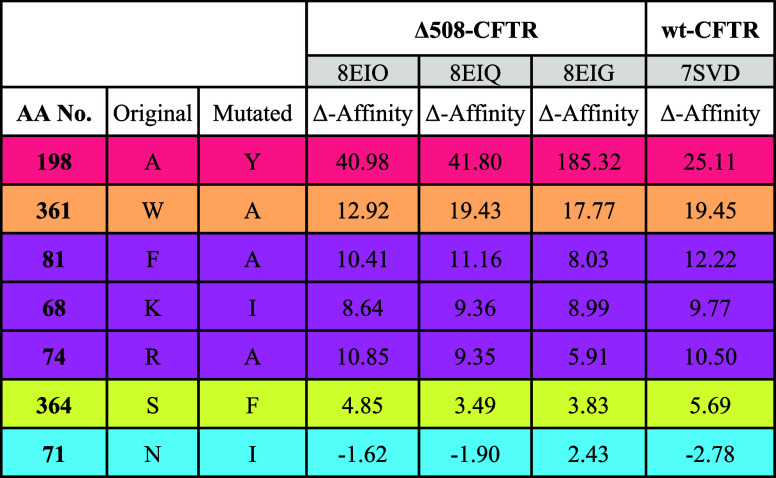
ΔAffinity Values^45^ for the Selected Single-Point Mutations on the Indicated PDB Structures of F508del- and wt-CFTR^a^

a The table colors are related to the binding residues shown in Figure 8.

Of the seven mutations analyzed, two were critical. First, A198Y significantly changed the pocket accessibility, with the volume change of the residue side chain being responsible for the increased Δaffinity in all investigated CFTR structures. Although the phenyl ring of the tyrosine side chain could form favorable contacts with the aromatic portion of *gem*-difluorobenzodioxole moiety of **ARN23765**, its steric hindrance prevented such interaction, suggesting that this accessible pocket space is important for ligand binding. Then, the pivotal role of W361 was demonstrated by the high Δaffinity value for the mutation to alanine (W361A) (Table 1). With the new methyl group located at the entrance of the hydrophobic cavity, the binding site loses its anchor point for the aromatic head and central linker of **ARN23765**, achieving a significantly worse ligand affinity, thus highlighting the key double π–π interaction with the corrector aromatic scaffold. The selected mutations F81A, K68I and R74A showed an intermediate impact on the ligand affinity. When F81 was replaced by alanine, the binding site lost its ability to cover the **ARN23765** aromatic linker, but the cavity remained accessible for binding, maintaining its hydrophobic nature. Although the changes in the binding site polarity could be considered significant, the mutations of K68 and R74 to lipophilic residues (i.e., isoleucine and alanine, respectively) only moderately increased the Δaffinity values. The marginal role of K68 was also demonstrated by simulating F508del/K68I-CFTR in complex with **ARN23765** (Figure S7). In absence of the former salt bridge interaction with the carboxyl functionality, the ligand showed a more pronounced conformational variability in its cavity (RMSDs up to 3 Å, Figure S7). In the presence of the replacing I68 mutation, the ligand remains stacked in its lipophilic cavity with the *gem*-difluorobenzodioxole. However, in this new configuration, the outwardly projected polar moiety tends to recover a stabilizing interaction with the proximal arginine (R74) via a new compensating salt bridge (Figure S7). The in silico mutation of S364 into a phenylalanine (S364F) resulted in a limited loss of Δaffinity value in the investigated CFTR structures and could therefore be considered marginal for insights into the binding site (Table 1). Finally, an irrelevant mutation (N71I)^19^ was used to test and validate the protocol.

### In Cells Single-Point Mutation Analysis within the CFTR Binding Site for ARN23765

**Figure 9:**
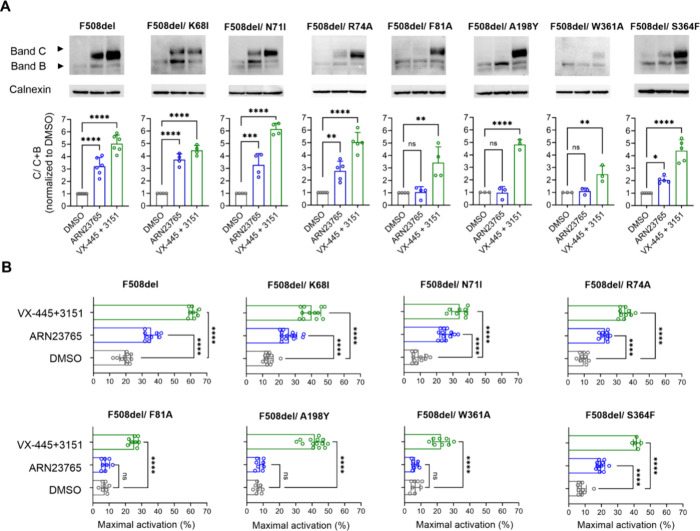
In-cell mutagenesis studies within the CFTR binding site for **ARN23765**. (A-B) HEK293 cells were transfected with the indicated F508del-CFTR double mutants and, after 24 h, were treated for a further 24 h with **ARN23765** (10 nM) or with a control mix of corrector type II, 3151 (10 μM), and corrector type III, VX-445 (3 μM). To enhance the mutated proteins’ rescue by correctors and to better appreciate any negative effect of the introduced mutations, incubation with compounds was performed at 30 °C. Analogous experiments performed at 37 °C are reported in Figure S8. (A) The maturation of CFTR double mutants was evaluated by WB. *Top*: representative WB images in which CFTR B- and C-band were detected. Calnexin was used as a loading control. *Bottom*: the abundance of band C was calculated with the conventional equation [C/C+B] and expressed as fold change of DMSO negative control (absence of corrector). (B) CFTR channel activity was evaluated by FLIPR assay. CFTR-mediated depolarization of the plasma membrane was detected as an increase in fluorescence after which the CFTR inhibitor, inh-172 (10 μM) was added. Bars report the peak change in fluorescence for the indicated mutants, expressed relative to baseline fluorescence. Representative traces are reported in Figure S9. For all experiments shown, mean ± SD of 3–6 replicates is reported. Statistical significance was calculated using one-way ANOVA and Dunnett’s multiple comparison test (DMSO vs corrector: nonsignificant (ns) *p* > 0.05; * *p* < 0.05; ** *p* < 0.01; *** *p* < 0.001; **** *p* < 0.0001).

To confirm in cells the above-predicted binding site for **ARN23765**, we introduced the investigated single-site mutations into the F508del-CFTR coding sequence. The rescue of double mutant proteins (F508del + new mutation) by **ARN23765** was assessed with WB analysis of protein maturation and evaluation of channel activity (FLIPR assay)^40^ in transfected HEK293 cells. Since correctors 3151 (type II)^16^ and VX-445 (type III)^18^ have been reported to bind to different CFTR sites, we used these compounds as controls. As expected, **ARN23765**-induced maturation of F508del-CFTR was not affected by the N71I substitution. In contrast, when point mutations F81A, W361A or A198Y were introduced into F508del-CFTR, the correction by **ARN23765** was markedly reduced or abolished. Indeed, the corrector was no longer able to promote protein maturation (Figure 9A and S8A). Notably, the substitution W361A was also critical for overall expression and correction of F508del protein by reference molecules, as already reported.^46^ Mutations R74A and S364F only slightly reduced **ARN23765**-induced protein maturation (Figure 9A), indicating that these amino acids may have a limited role in establishing binding contacts with the molecule. In line with MD simulations (Figure S7), no appreciable change in rescue by **ARN23765** was observed in the F508del/K68I-CFTR double mutant (Figure 9A and S8A). The results of protein maturation analysis were mostly confirmed by functional data (Figure 9B and S8B), in which the channel activity of double-mutated proteins was evaluated in terms of the change in cell membrane potential change (FLIPR assay).

## Discussion

**ARN23765** is a recently discovered potent CFTR small-molecule corrector, which has been licensed to a pharmaceutical company as a preclinical stage compound. Functional and biochemical studies have shown that **ARN23765** promotes F508del-CFTR stability and folding and thus partially rescues its function.^22^**ARN23765** belongs to a chemical class of compounds that was discovered via phenotypic screening. Therefore, identifying the molecular target(s) of this corrector could be crucial for understanding its mechanism of action and potentially discovering unknown mechanisms of CF disease, thus facilitating the development of innovative therapeutics.

To address this purpose, we chose live-cell photoaffinity labeling (PAL)^23^ as an innovative strategy for CF research to identify putative targets in a complete biological setting. We designed and synthesized **ARN23765**-derived PAPs, introducing onto the core scaffold of the parent compound, key reactive groups needed for either protein cross-linking (i.e., diazirine) or protein-probe adduct recovery (i.e., terminal alkyne or DS-biotin functionality). Diazirines upon UV irradiation at 350–380 nm release N_2_ generating a highly reactive carbene, which can react rapidly based on proximity by insertion into neighboring residues of the protein.^47,48^ However, due to their high reactivity they can get quenched fast either by water molecules or unspecific targets in a nonselective manner, or can isomerize by a 1,2-hydride shift, leading to low labeling yields.^23,26^ Nevertheless, for the design of the photoaffinity probes we favored diazirines due to their small-size and the possibility to be photoactivated at wavelengths that avoid biomolecules’ damage.^26^

Terminal alkyne VX-809-derived probes have previously been used to investigate the mechanism of action of VX-809 in cells.^49^ However, these probes have neither a photoactivatable moiety nor a chemically reactive electrophilic warhead, making it challenging to stably capture the interacting biomolecules, especially in a cellular environment. Recently, VX-770-derived biotinylated photoaffinity probes were used to identify their binding site on wt-CFTR in isolated cell membrane preparations.^50^ Although the putative VX-770 binding site was reported to be slightly different from the one previously identified by Yeh and colleagues,^42^ this discrepancy may arise from the use of alternative methodological approaches.

A possible pitfall when designing PAPs is that structural modifications of the original compound may reduce the affinity for the target and, eventually, the overall activity. Thus, we sought to introduce minimal perturbations into the **ARN23765** scaffold using the small-sized photoreactive diazirine ring and the terminal alkyne moiety needed for a two-step tag addition via CuAAC.^26^ We explored the introduction of these groups into different portions of the molecule, and we investigated their impact on compound activity. We found that the modification of the *gem*-difluorobenzodioxole moiety reduced the activity of the resulting molecules (e.g., **2**), whereas the replacement or functionalization of the carboxylic acid was tolerated well (e.g., **1**). As a purification tag, we selected DS-biotin, which lacking biotin’s tetrahydrothienyl ring, binds streptavidin with slightly less affinity than biotin.^51^ This modification allows using milder elution conditions, helpful in preserving CFTR, particularly its F508del mutant. Unfortunately, **ARN23765**-derived terminal alkyne PAPs (**PAP_1** and **PAP_2**) turned out to be ill-suited for CFTR identification. Indeed, we discovered that the protein is unstable under the copper-catalyzed experimental conditions needed to introduce the purification tag.

We then turned to DS-biot-labeled PAPs, thus avoiding the use of CuAAC protocol. The optimized protocol applied to **PAP_1 DS-biot** and **PAP_2 DS-biot** was successful in detecting both wt- and (most importantly) F508del-CFTR in cells, thus demonstrating the direct interaction of **ARN23765**-derived probes (and **ARN23765** itself, as shown by competition experiments) with CFTR in cells.

Correctors may interact with misfolded CFTR proteins to facilitate domain folding and/or assembly, thus decreasing its ubiquitylation (and therefore degradation), and enhancing the expression on the plasma membrane of a functional protein.^11^ We demonstrated that **ARN23765** directly stabilizes the MSD1 domain and improves overall CFTR folding by enhancing interactions between ICL4 (MSD2) and NBD1, as well as between ICL1 (MSD1) and NBD1. Previously, VX-809 was shown to modulate the ICL4-NBD1 and ICL1-NBD1 interfaces by stabilizing the MSD2 domain only when coexpressed with MSD1.^39^ Based on this finding, we speculated that **ARN23765**, similarly to VX-809, rescued F508del-CFTR folding by promoting a stable MSD1-MSD2 complex.

To identify the putative region of CFTR required for the binding of VX-809 or other CFTR correctors, CFTR fragments of different lengths have been employed by different laboratories. Current understanding suggests that VX-809 influences CFTR biogenesis at an early stage by binding to and modulating the conformation of MSD1.^35,52,53^ However, CFTR assembly requires cooperative domain folding, as evidenced by the fact that individual domains or certain domain combinations are largely non-native, leading to processing defects and ER retention. Specific domain–domain interactions, such as those between MSD1-NBD1-R-MSD2, are crucial for stabilizing CFTR and promoting its native-like state.^38,54^ Point mutations in domains like NBD1, MSD1, and MSD2 disrupt folding and destabilize the protein, explaining the misprocessing seen in numerous CFTR missense mutations, including F508del. Chaperones, such as Hsp70, Hsp90, and others, are known to assist in CFTR folding and mitigate misfolding caused by mutations like F508del. Therefore, modulating the activity of molecular chaperones, either through genetic manipulation or small-molecule inhibitors/activators, has shown promise in enhancing CFTR folding and promoting its transport to the plasma membrane.^55,56^ Although we did not directly investigate this aspect, our results suggest that **ARN23765** rescues F508del-CFTR by interacting directly with its MSD1. However, we cannot exclude that it might also modulate the activity of chaperones. Future studies will be needed to explore its potential effects on the activity of Hsp70, Hsp90 or other chaperones.

Cryo-electron microscopy (cryo-EM) data using constructs of purified wt- and mutant F508del-CFTR have shown that type I correctors (i.e., VX-809 and VX-661) enter a hydrophobic pocket in CFTR-MSD1, stabilizing this portion at an early stage of protein biogenesis and preventing its degradation.^19^ To provide a microscopically comprehensive description of **ARN23765**’s major interactions with previously reported binding pockets for F508del-CFTR correctors, we used computational techniques such as molecular docking and molecular dynamics simulations. From a structural perspective, **ARN23765** is similar to VX-809,^19^ VX-661,^19^ and GLPG-2222;^57^ namely, all these compounds share a similar *gem*-difluorobenzodioxole functionality. Other structural analogies include a polar group opposite the benzodioxole moiety (e.g., hydroxyl residues in VX-661 and C18 and the carboxyl functionality in VX-809 and GLPG-2222), and an aliphatic group (e.g., the trifluoromethyl in **ARN23765** or the methyl in VX-809) approximately eight carbon atoms from the benzodioxole head (Figure 1). VX-809 and VX-661 have been reported to intercalate in a hydrophobic and bent cavity buried between TM-1, TM-2, TM-3, and TM-6, engaging a sequence of Van der Walls interactions involving the *gem*-benzodioxole group.^19^ For both VX-809 and VX-661, the cyclopropane ring forces the molecule to adopt a 90° bent molecular shape, which perfectly fits into the lipophilic cavity, projecting the polar portion of the compounds outward. Similarly, **ARN23765**’s best-scoring pose demonstrated analogous interactions within type I corrector binding site, mostly overlapping with VX-809 and VX-661 binding modes (Figure 6A-B). The *gem*-difluorobenzodioxole was found to be wrapped by a few lipophilic residues, perfectly fitting into the same hydrophobic pocket as previously described.^19^ Furthermore, the tertiary amide functionality bends **ARN23765** in a *cis*-type conformation, allowing the two phenyl rings to embrace the W361 indole side-chain in a π–π stacking interaction, resulting in ligand stabilization. Contrary to previously reported type I correctors,^19^**ARN23765** shows a weaker interaction with R74 residue, which does not take part in the molecule’s stabilization.

Within the type III corrector binding site,^18^**ARN23765** tends to detach from the original interaction pattern, losing most of the initial binding interactions. This is shown by the MD analyses where the compound seemed to be constrained to this binding site by the action of the lipid bilayer only. Ultimately, when docked in the binding pocket for the potentiator VX-770, our corrector significantly changed the interaction patterns displaying only marginal interactions. This is in line with the functional data in the HS-YFP assay, highlighting no effect of **ARN23765** on the protein gating.^22^ A similar investigation on analogue **2**, bearing a trifluoromethyl-phenyl diazirine motif in place of the *gem*-difluorobenzodioxole moiety typical of **ARN23765**, showed lower affinity for the type I corrector binding site, while displaying relatively better preference for the potentiator binding site (Figure S6 and Tables S1–S2). This data is in line with the drop in activity observed for **2**, **PAP_2-** and **PAP_2 DS**-**biot**, which all lack a common *gem*-difluorobenzodioxole ring. Taken together, our findings demonstrate that this heterocyclic moiety is crucial for compound recognition in type I corrector binding site.

Overall, the in silico studies could help rationalizing **ARN23765**’s behavior as a type I corrector. Although the compound shares structural similarities with VX-809 and VX-661, it exhibits key characteristics. In particular, the molecular rigidity due to the trisubstituted tetrahydroindazole ring induces **ARN23765** to get a sandwiched conformation around the W361, emphasizing the crucial role of this amino acid and compensating the loss of π-cation interaction with the guanidine moiety of R74. Such peculiar structural pattern induces also a twisted conformation, contrasting with VX-770 planar structure thus preventing it from fitting into the potentiator pocket. **ARN23765** anionic character generates unique binding interactions at the type III corrector site, significantly altering its interaction pattern and resulting in a complex instability (Figure S10).

In silico and in vitro site-directed mutagenesis studies validated the putative binding site of **ARN23765** mapped with the computational analyses, highlighting a few amino acid residues that are essential for **ARN23765** rescue of F508del-CFTR. Namely, A198 was important for stabilizing the binding site, rather than for a direct connection with the compound. Residues W361 and F81 were proven to establish indispensable interactions with **ARN23765**. However, W361, seemed to be critical for the overall protein stability as already reported.^46^ Furthermore, of the two proximal positively charged residues (R74 and K68), involved in stabilizing the ligand via a salt bridge within the binding site pocket, the interaction with K68 was proven to be redundant. The guanidine-based residue (R74), which coordinates **ARN23765** carboxylic moiety, showed a compensatory effect offsetting the missing interaction with the lysine in the K68I mutant.

## Conclusions

In drug discovery, the elucidation of the protein target(s) of biologically active small molecules identified with phenotypic screening may be a challenging step in order to understand their mechanism of action.^58^ The search for CFTR modulators and their mode of action has mostly been conducted by evaluating the effect of compounds on the functional activity, cellular stability and maturation of mutant CFTR, or by investigating their interaction with purified full-length and/or CFTR domains.^59^ However, these strategies cannot conclusively provide information on the target(s) of CFTR modulators or the molecular basis of their function in cells. Given the multifaceted interactions of CFTR with the plasma membrane and the complexity of the protein network within the cellular compartments,^60^ in this study we verified the hypothesis of a direct binding of **ARN23765** with CFTR in live cells, and described its binding mode elucidating the molecular basis of **ARN23765**’s activity.

In summary, we have synthesized and validated **ARN23765**-derived probes for CFTR labeling in live cells. By in-cell PAL experiments, together with in-depth in silico and in cell investigations, we identified CFTR as a biological target for **ARN23765** and shed light on its putative binding site within the protein pocket for type I correctors, corroborating the functional results obtained with this corrector. Although we cannot rule out that other biological targets could also be engaged by **ARN23765**, our data advance the understanding of its biological activity in cells. To explore further cellular interplays and broaden the characterization of **ARN23765**, an unbiased target identification approach might be pursued.

## Experimental Section

### Chemistry

#### Synthetic Material and Methods

All solvents and reagents were obtained from commercial suppliers (Merck-Life Science, Carlo Erba, Alfa Aesar, Fluorochem, or Enamine) and used without further purification. 4-[3-(Trifluoromethyl)-3*H*-diazirin-3-yl]aniline hydrochloride was purchased from Enamine. CFTR modulators VX-809 and VX-661 (MedChemExpress), corr-4a (Sigma-Aldrich), corrector 3151 (LifeChemical) were obtained from commercial suppliers. VX-445 was synthesized in house.

Dry and deuterated solvents were purchased from Merck-Life Science. For simplicity, solvents and reagents are indicated as follows: acetonitrile (ACN), cyclohexane (Cy), dichloromethane (DCM), tetrahydrofuran (THF), ethyl acetate (EA), triethylamine (TEA), *N*,*N*-diisopropylethylamine (DIPEA). Reactions under anhydrous conditions were performed under argon atmosphere using oven-dried glassware and 3 cycles of vacuum/nitrogen.

All reactions involving photolabile compounds were performed in the dark (i.e., shielded by an aluminum foil) under an argon (Ar) atmosphere and the corresponding products were stored under inert Ar atmosphere at −21 °C to prevent decomposition. TLC analyses were performed using precoated TLC sheets ALUGRAM Xtra SIL G/UV254 from Macherey-Nagel. The visualization was done by UV light (254 nm) or staining with KMnO_4_. Automated column flash chromatography purifications were done using a Teledyne ISCO apparatus (CombiFlash Rf) with prepacked silica gel or neutral alumina columns cartridges of different sizes. Manual column chromatography purifications were carried out by using silica gel 60 Å, 40–63 μM (irregular shape).

NMR experiments of compounds were run on a Bruker Avance III 400 system (400 MHz for ^1^H NMR, 376 MHz for ^19^F) or Bruker Avance III 600 system (600 MHz for ^1^H NMR, 565 MHz for ^19^F) equipped with a BBI probe and Z-gradients. Spectra were acquired at 300 K using deuterated dimethyl sulfoxide (DMSO-*d*_*6*_, Sigma-Aldrich), as solvent. For intermediates and final compounds, chemical shifts for ^1^H NMR spectra were recorded in parts per million (ppm) using the residual non deuterated solvent as the internal standard (2.50 ppm). Data are reported as follows: chemical shift (ppm), multiplicity (indicated as b, broad; s, singlet; d, doublet; t, triplet; q, quartet; p, quintet, m, multiplet, and combinations thereof), coupling constants (J) in hertz (Hz), and integrated intensity. For final compounds, ^19^F- and ^1^H–^13^C HSQC (multiplicity edited Heteronuclear Single Quantum Coherence; 8 transients, 1024 data points, 256 increments) NMR data were also acquired.

UPLC-MS analyses of all the intermediates and final compounds were performed on Waters ACQUITY UPLC-MS system consisting of a single quadrupole detector (SQD) mass spectrometer equipped with an electrospray ionization (ESI) interface and a photodiode array detector (PDA) (from Waters Inc., Milford, MA, USA). ESI in positive and negative mode was applied in the mass scan range 100–1200 Da. The PDA range was 210–400 nm. The mobile phase was 10 mM NH_4_OAc in H_2_O at pH 5 adjusted with AcOH (A) and 10 mM NH_4_OAc in CH_3_CN-H_2_O (95:5) at pH 5 (B) with 0.5 mL/min as the flow rate. For intermediates, analyses were performed on an ACQUITY UPLC BEH C_18_ column (50 mm × 2.1 mm ID, particle size 1.7 μm) with a VanGuard BEH C_18_ precolumn (5 mm × 2.1 mm ID, particle size 1.7 μm). Analyses were performed with the following methods (flow rate 0.5 mL/min, temperature 40 °C): Generic method: initial hold at 5% in 0.2 min followed by a linear gradient 5–95% B over 2.5 min; Apolar method: initial hold at 50% in 0.2 min followed by a linear gradient 50–100% B over 2.5 min. Super-Apolar method: initial hold at 80% in 0.2 min followed by a linear gradient 80–100% B over 2.5 min. UPLC-MS analyses of the final compounds and intermediate **11** were performed using freshly prepared 10 mM DMSO-*d*_*6*_ stock solutions, diluted 20-fold in CH_3_CN/H_2_O (1:1) and directly analyzed. The analyses were performed on an ACQUITY UPLC BEH C_18_ column (100 mm × 2.1 mm ID, particle size: 1.7 μm) with a VanGuard BEH C_18_ precolumn (5 mm × 2.1 mm ID, particle size: 1.7 μm) at 40 °C using 10 mM NH_4_OAc in H_2_O at pH 5 adjusted with AcOH (A) and 10 mM NH_4_OAc in CH_3_CN-H_2_O (95:5) at pH 5 (B) as mobile phase at 0.5 mL/min.

For final compounds, high-resolution mass spectrometry (HRMS) for accurate mass measurements was performed on a Sciex TripleTOF high-resolution LC-MS using a Waters UPLC ACQUITY chromatographic system (from Waters Inc., Milford, MA, USA) coupled to a TripleTOF 5600+ mass spectrometer (from Sciex, Warrington, UK) equipped with a DuoSpray Ion source. The analyses were run on an ACQUITY UPLC BEH C_18_ column (50 × 2.1 mm ID, particle size 1.7 μm), using H_2_O + 0.1% HCOOH (A) and CH_3_CN + 0.1% HCOOH as mobile phase.

**ARN23765** was synthesized and characterized as previously reported.^22^ Purity of all final compounds (**1**, **2**, **PAP_1**, **PAP_2**, **PAP_1 DS-biot**, **PAP_2 DS-biot**, **PAP_N1 DS-biot**, **PAP_N2 DS-biot**) was determined by UPLC/MS and quantitative ^1^H NMR (qNMR, see the Supporting Information) analyses and was equal or greater than 95%, except for **PAP-2** (>90% purity).

### Synthesis of **ARN23765** Analogues **1** and **2**

#### (*R*)-Ethyl-3-(7-hydroxy-3-(trifluoromethyl)-4,5,6,7-tetrahydro-1*H*-indazol-1-yl)benzoate (**4**)

Formic acid (514 μL, 13.62 mmol) and dry TEA (944 μL, 6.77 mmol) were added dropwise to a stirred solution of ethyl 3-(7-oxo-3-(trifluoromethyl)-4,5,6,7-tetrahydro-1*H*-indazol-1-yl)benzoate (**3**)^29^ (1200.15 mg, 3.41 mmol) in dry 2-propanol (13 mL) at 0 °C under Ar atmosphere. RuCl(*p*-cymene)[(*R*,*R*)-Ts-DPEN] (22.0 mg, 0.035 mmol) was added and the reaction was stirred at room temperature for 18 h. Sat. aq. NH_4_Cl solution (10 mL) was added, and the mixture was extracted with ethyl acetate (3 × 10 mL), the combined organic layers were washed with brine (2 × 15 mL), dried over Na_2_SO_4_, and evaporated *in vacuo*. The crude product was purified by flash chromatography on a 24 g silica cartridge, using EA/Cy (0–12%) as eluent, to give **4** (1122.5 mg, 93%), as a yellow resin. UPLC-MS (Apolar method): *R*_t_ = 2.51 min. MS (ESI+) calcd. for C_17_H_18_F_3_N_2_O_3_ [M + H]^+^ 355.1; obsd. 355.1. ^1^H NMR (400 MHz, DMSO-*d*_*6*_) δ 8.38 (app t, *J* = 1.9 Hz, 1H), 8.13 (ddd, *J* = 8.1, 2.3, 1.1 Hz, 1H), 8.03 (dt, *J* = 7.8, 1.3 Hz, 1H), 7.72 (t, *J* = 7.9 Hz, 1H), 5.58 (d, *J* = 6.1 Hz, 1H), 4.75–4.72 (m, 1H), 4.36 (q, *J* = 7.2 Hz, 2H), 2.72–2.68 (m, 1H), 2.55–2.47 (m, 1H), 1.99–1.89 (m, 2H), 1.78–1.70 (m, 2H), 1.34 (t, *J* = 7.2 Hz, 3H).

#### (*S*)-Ethyl-3-(3-(trifluoromethyl)-7-(4-((trimethylsilyl)ethynyl)phenoxy)-4,5,6,7-tetrahydro-1*H*-indazol-1-yl)benzoate (**5**)

To a stirred solution of **4** (1032.2 mg, 2.91 mmol) in dry THF (10 mL), PMe_3_ (1.0 M in THF) (4.4 mL, 4.4 mmol) and a freshly prepared solution of 4-((trimethylsilyl)ethynyl)phenol^30^ (831.6 mg, 4.4 mmol) in dry THF (5.0 mL) were added. DIAD (860 μL, 4.4 mmol) was added dropwise at 0 °C under Ar atmosphere, and the reaction was stirred at room temperature for 18 h. Water (20 mL) was added, and the mixture was extracted with ethyl acetate (3 × 20 mL), the combined organic layers were washed with brine (2 × 50 mL), dried over Na_2_SO_4_, and evaporated *in vacuo*. The crude product was purified by flash chromatography on a 24 g silica cartridge, using EA/Cy (0–3.1%) as eluent, to give **5** (1163.0 mg, 76%), as white solid. UPLC-MS (Super-Apolar method): *R*_t_ = 2.18 min. MS (ESI+) calcd. for C_28_H_30_F_3_N_2_O_3_Si [M + H]^+^ 527.2; obsd. 527.2. ^1^H NMR (400 MHz, DMSO-*d*_6_) δ 8.06 (app t, *J* = 1.9 Hz, 1H), 7.94 (dt, *J* = 7.8, 1.3 Hz, 1H), 7.84 (ddd, *J* = 8.1, 2.3, 1.1 Hz, 1H), 7.56 (t, *J* = 7.9 Hz, 1H), 7.34–7.30 (m, 2H), 6.88–6.84 (m, 2H), 5.76–5.74 (m, 1H), 4.24–4.11 (m, 2H), 2.81–2.77 (m, 1H), 2.64–2.57 (m, 1H), 2.09–2.03 (m, 1H), 1.91–1.81 (m, 3H), 1.18 (t, *J* = 7.2 Hz, 3H), 0.20 (s, 9H).

#### (*S*)-3-(7-(4-Ethynylphenoxy)-3-(trifluoromethyl)-4,5,6,7-tetrahydro-1*H*-indazol-1-yl)benzoic acid (**6**)

To a stirred solution of **5** (1160.9 mg, 2.20 mmol) in THF:MeOH (1:1, 12 mL), a freshly prepared 1.1 M solution of LiOH in water (6 mL, 6.6 mmol) was added, and the reaction was stirred at room temperature for 18 h. The mixture was concentrated *in vacuo*, the residue was taken up in THF (6.0 mL), treated with a freshly prepared 1.1 M solution of LiOH in water (3 mL, 3.3 mmol) and stirred at room temperature for further 18 h. The mixture was concentrated *in vacuo*, the residue was cooled to 0 °C, treated dropwise with 2.0 M HCl until pH = 5–6, and extracted with ethyl acetate (3 × 20 mL). The combined organic layers were dried over Na_2_SO_4_ and evaporated *in vacuo* to afford **6** (940.0 mg, *quant*.), as a white-beige solid, which was used in the next step without further purification. UPLC-MS (Apolar method): *R*_t_ = 1.14 min. MS (ESI-) calcd. for C_23_H_16_F_3_N_2_O_3_ [M-H]^−^ 425.1; obsd. 425.1. ^1^H NMR (400 MHz, DMSO-*d*_*6*_) δ 8.09 (app t, *J* = 1.9 Hz, 1H), 7.93 (app d, *J* = 7.8 Hz, 1H), 7.80 (app dd, *J* = 7.9, 2.3 Hz, 1H), 7.52 (t, *J* = 7.9 Hz, 1H), 7.37–7.33 (m, 2H), 6.91–6.89 (m, 2H), 5.76–5.74 (m, 1H), 4.01 (s, 1H), 2.81–2.77 (m, 1H), 2.64–2.55 (m, 1H), 2.13–2.03 (m, 1H), 1.91–1.79 (m, 3H).

#### (*S*)-*N*-(2,2-Difluorobenzo[*d*][1,3]dioxol-5-yl)-3-(7-(4-ethynylphenoxy)-3-(trifluoromethyl)-4,5,6,7-tetrahydro-1*H*-indazol-1-yl)-*N*-methylbenzamide (**1**)

To a stirred solution of **6** (198.0 mg, 0.46 mmol) in dry ethyl acetate (4.2 mL), 1-propanephosphonic anhydride (T_3_P) 50 wt % in ethyl acetate (410 μL, 0.7 mmol) and dry DIPEA (200 μL, 1.15 mmol) were added at room temperature under Ar atmosphere. The mixture was cooled to 0 °C, 2,2-difluoro-*N*-methylbenzo[*d*][1,3]dioxol-5-amine hydrochloride was added, and the reaction was stirred at room temperature for 18 h. Water was added (4 mL) and the mixture was extracted with ethyl acetate (3 × 5 mL), the combined organic layers were dried over Na_2_SO_4_, and evaporated *in vacuo*. The crude product was purified by flash chromatography on a 12 g silica cartridge, using EA/Cy (0–28%) as eluent. The resulting product was further purified by another flash chromatography on a 24 g neutral alumina cartridge, using EA/Cy (0–13%) as eluent. The product was lyophilized from ACN:water (1:3) to afford **1** (243.0 mg, 88%), as white solid. UPLC-MS (Apolar method): *R*_t_ = 4.61 min. MS (ESI+) calcd. for C_31_H_23_F_5_N_3_O_4_ [M + H]^+^ 596.16; obsd. 596.08. HRMS (ESI+) *m*/*z*: calcd. for C_31_H_23_F_5_N_3_O_4_ [M + H]^+^, 596.1603; found 596.1616. ^1^H NMR (400 MHz, DMSO-*d*_6_) δ 7.55 (bs, 1H), 7.52–7.45 (m, 1H), 7.40 (d, *J* = 2.1 Hz, 1H), 7.38–7.33 (m, 2H), 7.30–7.21 (m, 2H), 7.18 (d, *J* = 8.6 Hz, 1H), 6.90–6.84 (m, 2H), 6.79 (d, *J* = 8.6 Hz, 1H), 5.70–5.65 (m, 1H), 4.02 (s, 1H), 3.25 (s, 3H), 2.82–2.73 (m, 1H), 2.63–2.53 (m, 1H), 2.11–2.02 (m, 1H), 1.92–1.77 (m, 3H). ^19^F (376 MHz, DMSO-*d*_6_) δ −48.94, −60.45.

#### (*S*)-Ethyl-3-(7-(4-(*tert*-butoxycarbonyl)phenoxy)-3-(trifluoromethyl)-4,5,6,7-tetrahydro-1*H*-indazol-1-yl)benzoate (**7**)

To a stirred solution of **4** (999.8 mg, 2.82 mmol) in dry THF (10 mL), PMe_3_ (1.0 M in THF) (4.2 mL, 4.23 mmol) and a freshly prepared solution of *tert*-butyl 4-hydroxybenzoate (822.1 mg, 4.23 mmol) in dry THF (4.0 mL) were added. DIAD (833 μL, 4.23 mmol) was added dropwise at 0 °C under Ar atmosphere, and the reaction was stirred at room temperature for 18 h. Sat. aq. NH_4_Cl solution (20 mL) was added, and the mixture was extracted with ethyl acetate (3 × 20 mL). The combined organic layers were washed with brine (2 × 50 mL), dried over Na_2_SO_4_, and evaporated *in vacuo*. The crude product was purified by flash chromatography on a 24 g silica cartridge, using EA/heptane (0–6%) as eluent. To discard traces of *tert*-butyl 4-hydroxybenzoate, the obtained product was taken up in EA (50 mL), washed with 1.0 M NaOH (2 × 50 mL), dried over Na_2_SO_4_, and evaporated *in vacuo* to give **7** (993.0 mg, 66%), as off-white solid. UPLC-MS (Super-Apolar method): *R*_t_ = 1.59 min. MS (ESI+) calcd. for C_28_H_28_F_3_N_2_O_5_ [M + H]^+^ 531.2; obsd. 531.0. ^1^H NMR (400 MHz, DMSO-*d*_6_) δ 8.04 (app t, *J* = 1.9 Hz, 1H), 7.93 (dt, *J* = 7.8, 1.3 Hz, 1H), 7.84 (ddd, *J* = 8.1, 2.3, 1.1 Hz, 1H), 7.80–7.75 (m, 2H), 7.58 (t, *J* = 7.9 Hz, 1H), 6.99–6.93 (m, 2H), 5.85–5.82 (m, 1H), 4.23–4.08 (m, 2H), 2.84–2.75 (m, 1H), 2.68–2.56 (m, 1H), 2.13–2.04 (m, 1H), 1.97–1.80 (m, 3H), 1.52 (s, 9H), 1.16 (t, *J* = 7.2 Hz, 3H).

#### (*S*)-3-(7-(4-(*tert*-Butoxycarbonyl)phenoxy)-3-(trifluoromethyl)-4,5,6,7-tetrahydro-1*H*-indazol-1-yl)benzoic Acid (**8**)

To a stirred solution of **7** (993.0 mg, 1.87 mmol) in THF (13 mL), a freshly prepared 0.88 M solution of LiOH in water (6.5 mL, 5.6 mmol) was added, and the reaction was stirred at room temperature for 18 h. Another aliquot of freshly prepared 0.88 M solution of LiOH in water (2 mL, 1.8 mmol) was added, and the reaction was stirred at room temperature for further 1 h. The mixture was cooled to 0 °C, treated dropwise with 2.0 M HCl until pH = 4–5, and extracted with DCM (3 × 20 mL). The combined organic layers were dried over Na_2_SO_4_, and evaporated *in vacuo*. The obtained product was washed with *n*-pentane to afford **8** (940.0 mg, *quant*.), as an off-white solid, which was used in the next step without further purification. UPLC-MS (Apolar method): *R*_t_ = 1.46 min. MS (ESI+) calcd. for C_26_H_26_F_3_N_2_O_5_ [M + H]^+^ 503.2; obsd. 502.9. ^1^H NMR (400 MHz, DMSO-*d*_6_) δ 8.09 (app t, *J* = 1.9 Hz, 1H), 7.92 (dt, *J* = 7.8, 1.3 Hz, 1H), 7.71–7.75 (m, 3H), 7.52 (t, *J* = 7.9 Hz, 1H), 7.00–6.94 (m, 2H), 5.85–5.79 (m, 1H), 2.85–2.75 (m, 1H), 2.68–2.55 (m, 1H), 2.13–2.06 (m, 1H), 1.96–1.79 (m, 3H), 1.52 (s, 9H).

#### (*S*)-*tert*-Butyl-4-((3-(trifluoromethyl)-1-(3-((4-(3-(trifluoromethyl)-3H-diazirin-3-yl)phenyl)carbamoyl)phenyl)-4,5,6,7-tetrahydro-1*H*-indazol-7-yl)oxy)benzoate (**9**)

PPh_3_ (210.0 mg, 0.80 mmol) and trichloroacetonitrile (80 μL, 0.80 mmol) were added to a stirred solution of **8** in dry DCM (12.5 mL) at 0 °C, and the reaction was stirred at room temperature for 1.5 h under Ar atmosphere. The mixture was concentrated *in vacuo*, the residue was taken up in dry DCM (3.0 mL) and added to a stirred solution of 4-[3-(trifluoromethyl)-3*H*-diazirin-3-yl]aniline hydrochloride (114.6 mg, 0.48 mmol) and TEA (167 μL, 1.2 mmol) in dry DCM (3.0 mL) at 0 °C under Ar atmosphere in the dark. The ice bath was allowed to melt, and the mixture was stirred at room temperature for 1.5 h under Ar atmosphere in the dark. Sat. aq. NH_4_Cl solution (5 mL) was added, and the mixture was extracted with DCM (3 × 6 mL) and ethyl acetate (2 × 5 mL). The combined organic layers were dried over Na_2_SO_4_ and evaporated *in vacuo*. The crude product was purified by flash chromatography on a 24 g silica cartridge, using EA/Cy (0–10%) as eluent, to give **9** (186.7 mg, 68%), as a white-yellow solid. UPLC-MS (Super-Apolar method): *R*_t_ = 1.85 min. MS (ESI-) calcd. for C_34_H_28_F_6_N_5_O_4_ [M-H]^−^ 684.2; obsd. 684.1. ^1^H NMR (400 MHz, DMSO-*d*_*6*_) δ 10.40 (s, 1H), 8.10 (app t, *J* = 1.9 Hz, 1H), 7.93 (dt, *J* = 7.9, 1.4 Hz, 1H), 7.82–7.76 (m, 3H), 7.66–7.61 (m, 2H), 7.58 (t, *J* = 7.9 Hz, 1H), 7.25 (d, *J* = 8.5 Hz, 2H), 6.98–6.90 (m, 2H), 5.81–5.76 (m, 1H), 2.87–2.75 (m, 1H), 2.69–2.56 (m, 1H), 2.14–2.04 (m, 1H), 1.99–1.79 (m, 3H), 1.47 (s, 9H).

#### (*S*)-*tert*-Butyl-4-((1-(3-(methyl(4-(3-(trifluoromethyl)-3*H*-diazirin-3-yl)phenyl)carbamoyl)phenyl)-3-(trifluoromethyl)-4,5,6,7-tetrahydro-1H-indazol-7-yl)oxy)benzoate (**10**)

Cs_2_CO_3_ (74.0 mg, 0.23 mmol) and MeI (14 μL, 0.22 mmol) were added to a well-stirred solution of **9** (79.0 mg, 0.12 mmol) in dry DMF (1.4 mL) at 0 °C under Ar in the dark. The mixture was stirred at room temperature for 18 h under Ar atmosphere in the dark. Sat. aq. NH_4_Cl solution (2 mL) was added, the mixture was extracted with ethyl acetate (3 × 5 mL), the combined organic layers were washed with LiCl 10% (3 × 8 mL), dried over Na_2_SO_4_, and evaporated *in vacuo* to provide **10** (78.9 mg, 94%), as a dark yellow oil, which was used in the next step without further purification. UPLC-MS (Super-Apolar method): *R*_t_ = 1.48 min. MS (ESI+) calcd. for C_31_H_23_F_5_N_3_O_4_ [M + H]^+^ 700.2; obsd. 700.0. ^1^H NMR (400 MHz, DMSO-*d*_*6*_) δ 7.82 (d, *J* = 8.8 Hz, 2H), 7.57 (app t, *J* = 1.9 Hz, 1H), 7.50 (app d, *J* = 8.2 Hz, 1H), 7.23 (t, *J* = 7.9 Hz, 1H), 7.12–6.94 (m, 7H), 5.81–5.77 (m, 1H), 3.24 (s, 3H), 2.83–2.74 (m, 1H), 2.65–2.54 (m, 1H), 2.15–2.06 (m, 1H), 1.96–1.76 (m, 3H), 1.48 (s, 9H).

#### (*S*)-4-((1-(3-(Methyl(4-(3-(trifluoromethyl)-3*H*-diazirin-3-yl)phenyl)carbamoyl)phenyl)-3-(trifluoromethyl)-4,5,6,7-tetrahydro-1H-indazol-7-yl)oxy)benzoic Acid (**2**)

TFA (81 μL, 1.06 mmol) was added dropwise to a stirred solution of **10** (42.4 mg, 0.061 mmol) in dry DCM (400 μL) at 0 °C under Ar in the dark. The ice bath was allowed to melt, and the mixture was stirred at room temperature for 4 h under Ar in the dark. The mixture was diluted with DCM (2.0 mL) and 2.0 M HCl (2.0 mL), extracted with DCM (3 × 5 mL), and the combined organic layers were dried over Na_2_SO_4_, and evaporated *in vacuo*. The crude product was purified by flash chromatography on a 4 g silica cartridge, using (10% MeOH/DCM)/DCM (0–19%) as eluent. The compound was triturated with 20% EA in *n*-pentane, and lyophilized from ACN:water (1:3) to afford **2** (22.0 mg, 56%), as white solid. UPLC-MS (Generic method): *R*_t_ = 5.44 min. MS (ESI+) calcd. for C_31_H_24_F_6_N_5_O_4_ [M + H]^+^ 644.17; obsd. 644.39. HRMS (ESI+) *m*/*z*: calcd. for C_31_H_24_F_6_N_5_O_4_ [M + H]^+^, 644.1727; found 644.1741. ^1^H NMR (400 MHz, DMSO-*d*_6_) δ 7.88–7.82 (m, 2H), 7.56–7.53 (m, 1H), 7.52–7.48 (m, 1H), 7.24 (t, *J* = 7.8 Hz, 1H), 7.17–7.07 (m, 3H), 7.00 (dd, *J* = 12.0, 8.5, 4H), 5.80–5.75 (m, 1H), 3.23 (s, 3H), 2.83–2.74 (m, 1H), 2.64–2.54(m, 1H), 2.15–2.07 (m, 1H), 1.94–1.76 (m, 3H). ^19^F (376 MHz, DMSO-*d*_6_) δ −60.41, - 64.67.

### Synthesis of **ARN23765**-Derived Alkyne-Substituted Photoaffinity Probes (**PAP_1** and **PAP_2)**

#### (*S*)-3-(7-(4-((2-(3-(But-3-yn-1-yl)-3*H*-diazirin-3-yl)ethyl)carbamoyl)phenoxy)-3-(trifluoromethyl)-4,5,6,7-tetrahydro-1H-indazol-1-yl)-*N*-(2,2-difluorobenzo[*d*][1,3]dioxol-5-yl)-*N*-methylbenzamide (**PAP_1**)

To a stirred solution of **ARN23765** (185.6 mg, 0.30 mmol) in dry DMF (2.0 mL), DIPEA (80 μL, 0.46 mmol) and HATU (137.6 mg, 0.36 mmol) were added. The mixture was stirred for 10 min and then cooled to 0 °C and treated with a freshly prepared solution of 2-(3-(but-3-yn-1-yl)-3*H*-diazirin-3-yl)ethan-1-amine^34^ (45.5 mg, 0.33 mmol) in dry DMF (3.0 mL) under Ar in the dark. The reaction was stirred at room temperature for 18 h under Ar in the dark. Sat. aq. NH_4_Cl solution (5 mL) was added, the mixture was extracted with ethyl acetate (3 × 10 mL), the combined organic layers were washed with LiCl 10% (3 × 15 mL), dried over Na_2_SO_4_, and evaporated *in vacuo*. The crude product was purified by flash chromatography on a 12 g silica cartridge, using EA/Cy (0–36%), as eluent. The obtained compound was taken up in ethyl acetate (30 mL) and washed with water (4 × 20 mL), organic layer was dried over Na_2_SO_4_, evaporated *in vacuo,* and lyophilized from ACN:water (1:3) to afford **PAP_1** (186.7 mg, 84%), as a white powder. UPLC-MS (Apolar method): *R*_t_ = 3.19 min. MS (ESI+) calcd. for C_37_H_32_F_5_N_6_O_5_ [M + H]^+^ 735.23; obsd. 735.21. HRMS (ESI+) *m*/*z*: calcd. for C_37_H_32_F_5_N_6_O_5_ [M + H]^+^, 735.2349; found 735.2349. ^1^H NMR (400 MHz, DMSO-*d*_6_) δ 8.29 (t, *J* = 5.6 Hz, 1H), 7.80–7.75 (m, 2H), 7.56 (s, 1H), 7.51 (d, *J* = 7.1 Hz, 1H), 7.36 (d, *J* = 2.1 Hz, 1H), 7.31–7.21 (m, 2H), 7.13 (d, *J* = 8.6 Hz, 1H), 7.00–6.93 (m, 2H), 6.72 (d, *J* = 8.6 Hz, 1H), 5.74–5.69 (m, 1H), 3.22 (s, 3H), 3.17–3.08 (m, 2H), 2.81–2.77 (m, 2H), 2.65–2.53 (m, 1H), 2.14–2.06 (m, 1H), 1.99 (td, *J* = 7.4, 2.7 Hz, 2H), 1.93–1.77 (m, 3H), 1.61 (q, *J* = 6.8 Hz, 4H). ^19^F (376 MHz, DMSO-*d*_6_) δ −48.95, - 60.44.

#### (*S*)-3-(7-(4-Ethynylphenoxy)-3-(trifluoromethyl)-4,5,6,7-tetrahydro-1*H*-indazol-1-yl)-*N*-(4-(3-(trifluoromethyl)-3*H*-diazirin-3-yl)phenyl)benzamide (**11**)

PPh_3_ (254.7 mg, 0.97 mmol) and trichloroacetonitrile (97 μL, 0.97 mmol) were added to a stirred solution of **6** (207.0 mg, 0.49 mmol) in dry DCM (15.0 mL) at 0 °C, and the reaction was stirred at room temperature for 2 h under Ar. The mixture was concentrated *in vacuo*, the residue was taken up in dry DCM (3.5 mL) and added to a stirred solution of 4-[3-(trifluoromethyl)-3*H*-diazirin-3-yl]aniline hydrochloride (139.7 mg, 0.59 mmol) and TEA (203 μL, 1.5 mmol) in dry DCM (3.0 mL) at 0 °C under Ar atmosphere in the dark. The ice bath was allowed to melt, and the mixture was stirred at room temperature for 1.5 h under Ar atmosphere in the dark. Sat. aq. NH_4_Cl solution (5 mL) was added, and the mixture was extracted with DCM (3 × 6 mL) and ethyl acetate (2 × 5 mL), the combined organic layers were dried over Na_2_SO_4_, and evaporated *in vacuo*. The crude product was purified by flash chromatography on a 24 g silica cartridge, using (10% EA in Cy)/Cy (0–98.5%), as eluent, to give **11** (239.5 mg, 80%), as a white solid. UPLC-MS (Apolar method): *R*_t_ = 5.21 min. MS (ESI-) calcd. for C_31_H_20_F_6_N_5_O_2_ [M-H]^−^ 608.15; obsd. 608.23. ^1^H NMR (400 MHz, DMSO-*d*_*6*_) δ 10.46 (s, 1H), 8.11 (app t, *J* = 1.9 Hz, 1H), 7.95 (dt, *J* = 7.8, 1.3 Hz, 1H), 7.84–7.77 (m, 3H), 7.57 (t, *J* = 7.9 Hz, 1H), 7.27 (d, *J* = 8.4 Hz, 2H), 7.23–7.18 (m, 2H), 6.91–6.84 (m, 2H), 5.75–5.71 (m, 1H), 3.97 (s, 1H), 2.86–2.77 (m, 1H), 2.68–2.57 (m, 1H), 2.13–2.05 (m, 1H), 1.93–1.80 (m, 3H).

#### (*S*)-3-(7-(4-Ethynylphenoxy)-3-(trifluoromethyl)-4,5,6,7-tetrahydro-1*H*-indazol-1-yl)-*N*-methyl-*N*-(4-(3-(trifluoromethyl)-3*H*-diazirin-3-yl)phenyl)benzamide (**PAP_2**)

Cs_2_CO_3_ (57.2 mg, 0.18 mmol) and MeI (11 μL, 0.18 mmol) were added to a stirred solution of **11** (53.5 mg, 0.09 mmol) in dry DMF (1.0 mL) at 0 °C under Ar in the dark. The ice bath was allowed to melt, and the mixture was stirred at room temperature for 2.5 h under Ar atmosphere in the dark. Sat. aq. NH_4_Cl solution (1.0 mL) was added, and the mixture was extracted with ethyl acetate (3 × 5 mL), the combined organic layers were washed with LiCl 10% (3 × 8 mL), dried over Na_2_SO_4_, and evaporated *in vacuo.* The crude product was purified by flash chromatography on a 4 g silica cartridge, using EA/Cy (0–14%) as eluent, to give **PAP_2** (43.7 mg, 80%), as a white-yellow solid. UPLC-MS (Apolar method): *R*_t_ = 5.09 min. MS (ESI+) calcd. for C_32_H_24_F_6_N_5_O_2_ [M + H]^+^ 624.18; obsd. 621.12. HRMS (ESI+) *m*/*z*: calcd. for C_32_H_24_F_6_N_5_O_2_ [M + H]^+^, 624.1829; found 624.1830. ^1^H NMR (600 MHz, DMSO-*d*_*6*_) δ 7.54 (app t, *J* = 1.9 Hz, 1H), 7.52–7.48 (m, 1H), 7.40–7.36 (m, 2H), 7.26 (t, *J* = 7.8, 1H), 7.16 (d, *J* = 8.0 Hz, 1H), 7.14–7.11 (m, 2H), 7.06 (d, *J* = 8.3 Hz, 2H), 6.95–6.90 (m, 2H), 5.72–5.70 (m, 1H), 4.04 (s, 1H), 3.25 (s, 3H), 2.81–2.74 (m, 1H), 2.63–2.54 (m, 1H), 2.11–2.05 (m, 1H), 1.88–1.77 (m, 3H). ^19^F (565 MHz, DMSO-*d*_6_) δ −59.46, - 63.67.

### Synthesis of **ARN23765**-Derived Desthiobiotinylated Photoaffinity Probes (**PAP_1 DS-biot**, **PAP_N1 DS-biot**, **PAP_2 DS-biot**, and **PAP_N2 DS-biot**)

#### *N*-(2,2-Difluorobenzo[*d*][1,3]dioxol-5-yl)-*N*-methyl-3-((*S*)-7-(4-((2-(3-(2-(1-(18-((4*S*,5*R*)-5-methyl-2-oxoimidazolidin-4-yl)-13-oxo-3,6,9-trioxa-12-azaoctadecyl)-1*H*-1,2,3-triazol-4-yl)ethyl)-3*H*-diazirin-3-yl)ethyl)carbamoyl)phenoxy)-3-(trifluoromethyl)-4,5,6,7-tetrahydro-1*H*-indazol-1-yl)benzamide (**PAP_1 DS-biot**)

A freshly prepared 0.1 M solution of desthiobiotin-PEG3-azide in dry THF (286 μL, 0.0286 mmol) was added to **PAP_1** (21.0 mg, 0.0286 mmol) under Ar in the dark. *Tert*-butanol (175 μL), water (175 μL), a freshly prepared 1.0 M solution of CuSO_4_**·** 5H_2_O in water (5.7 μL, 0.0057 mmol), and a freshly prepared 2.0 M solution of sodium ascorbate in water (1.7 μL, 0.0034 mmol) were sequentially added, and the reaction was stirred at room temperature for 18 h in the dark. Water (2.0 mL) was added, and the mixture was extracted with DCM (3 × 5.0 mL), before the combined organic layers were washed with brine (2 × 5 mL), dried over Na_2_SO_4_, and evaporated *in vacuo.* The crude product was purified by flash chromatography on a 4 g silica cartridge, using 10% MeOH in DCM/DCM (0–100%), as eluent. The obtained product was lyophilized from ACN:water (1:3) to afford **PAP_1 DS-biot** (19.6 mg, 60%), as a white powder. UPLC-MS (Generic method): *R*_t_ = 5.12 min. MS (ESI+) calcd. for C_55_H_66_F_5_N_12_O_10_ [M + H]^+^ 1149.19; obsd. 1149.36. HRMS (ESI+) *m*/*z*: calcd. for C_55_H_66_F_5_N_12_O_10_ [M + H]^+^, 1149.1940; found 1149.4970. ^1^H NMR (400 MHz, DMSO) δ 8.32 (t, *J* = 5.6 Hz, 1H), 7.84–7.74 (m, 3H), 7.58–7.46 (m, 2H), 7.37 (d, *J* = 2.1 Hz, 1H), 7.31–7.20 (m, 2H), 7.13 (d, *J* = 8.7 Hz, 1H), 6.96 (d, *J* = 8.8 Hz, 2H), 6.73 (d, *J* = 8.6 Hz, 1H), 6.28 (s, 1H), 6.11 (s, 1H), 5.75–5.68 (m, 1H), 4.46 (t, *J* = 5.3 Hz, 2H), 3.77 (t, *J* = 5.3 Hz, 2H), 3.59 (p, *J* = 6.4 Hz, 1H), 3.53–3.42 (m, 7H), 3.25–3.08 (m, 6H), 2.83–2.73 (m, 1H), 2.64–2.54 (m, 1H), 2.41 (dd, *J* = 9.1, 6.8 Hz, 2H), 2.13–2.00 (m, 3H), 1.92–1.72 (m, 5H), 1.60 (t, *J* = 7.2 Hz, 2H), 1.45 (q, *J* = 7.3 Hz, 2H), 1.38–1.09 (m, 7H), 0.94 (d, *J* = 6.4 Hz, 3H). ^19^F (376 MHz, DMSO-*d*_6_) δ −48.96, −60.44.

#### (*S*)-*tert*-Butyl-4-((1-(3-((2,2-difluorobenzo[*d*][1,3]dioxol-5-yl)carbamoyl)phenyl)-3-(trifluoromethyl)-4,5,6,7-tetrahydro-1*H*-indazol-7-yl)oxy)benzoate (**12**)

To a stirred solution of **8** (154.9 mg, 0.31 mmol) in dry DMF (1.5 mL), DIPEA (81 μL, 0.47 mmol) and HATU (140.7 mg, 0.37 mmol) were added. The mixture was stirred for 10 min and then cooled to 0 °C and treated with a freshly prepared solution of 2,2-difluoro-5-aminobenzodioxole (59.0 mg, 0.34 mmol) in dry DMF (2.0 mL) under Ar in the dark. The reaction was stirred at room temperature for 18h under Ar in the dark. Sat. aq. NH_4_Cl solution (5 mL) was added, the mixture was extracted with ethyl acetate (3 × 10 mL), the combined organic layers were washed with LiCl 10% (3 × 15 mL), dried over Na_2_SO_4_, and evaporated *in vacuo*. The crude product was purified by flash chromatography on a 12 g silica cartridge, using EA/Cy (0–9%) as eluent, to provide **12** (172.9 mg, 85%), as a yellowish solid. UPLC-MS (Apolar method): *R*_t_ = 2.66 min. MS (ESI-) calcd. for C_33_H_27_F_5_N_3_O_6_ [M-H]^−^ 656.2; obsd. 656.4. ^1^H NMR (400 MHz, DMSO-*d*_6_) δ 10.34 (s, 1H), 8.08 (app t, *J* = 1.9 Hz, 1H), 7.94–7.89 (m, 1H), 7.82–7.72 (m, 2H), 7.67–7.56 (m, 3H), 7.39–7.34 (m, 2H), 6.98–6.93 (m, 2H), 5.82–5.77 (m, 1H), 2.88–2.77 (m, 1H), 2.68–2.58 (m, 1H), 2.14–2.06 (m, 1H), 1.97–1.81 (m, 3H), 1.46 (s, 9H).

#### (*S*)-4-((1-(3-((2,2-Difluorobenzo[*d*][1,3]dioxol-5-yl)carbamoyl)phenyl)-3-(trifluoromethyl)-4,5,6,7-tetrahydro-1*H*-indazol-7-yl)oxy)benzoic Acid (**13**)

TFA (300 μL, 3.92 mmol) was added dropwise to a stirred solution of **12** (158.8 mg, 0.24 mmol) in dry DCM (1.6 mL) at 0 °C under Ar in the dark. The ice bath was allowed to melt, and the mixture was stirred at room temperature for 3 h under Ar in the dark. The mixture was diluted with DCM (5.0 mL) and 2.0 M HCl (5.0 mL), extracted with DCM (3 × 10 mL). The combined organic layers were dried over Na_2_SO_4_, and evaporated *in vacuo* to afford **13** (145.0 mg, *quant*.), as beige solid, which was used in the next step without further purification. UPLC-MS (Apolar method): *R*_t_ = 1.43 min. MS (ESI+) calcd. for C_29_H_21_F_5_N_3_O_6_ [M + H]^+^ 602.1; obsd. 602.2.

#### (*S*)-3-(7-(4-((2-(3-(But-3-yn-1-yl)-3*H*-diazirin-3-yl)ethyl)carbamoyl)phenoxy)-3-(trifluoromethyl)-4,5,6,7-tetrahydro-1*H*-indazol-1-yl)-*N*-(2,2-difluorobenzo[*d*][1,3]dioxol-5-yl)benzamide (**14**)

To a stirred solution of **13** (25.0 mg, 0.042 mmol) in dry DMF (500 μL), DIPEA (11 μL, 0.063 mmol) and HATU (19.0 mg, 0.05 mmol) were added. The mixture was stirred for 10 min and then cooled to 0 °C and treated with a freshly prepared solution of 2-(3-(but-3-yn-1-yl)-3*H*-diazirin-3-yl)ethan-1-amine^34^ (7.0 mg, 0.051 mmol) in dry DMF (800 μL) under Ar in the dark. The reaction was stirred at room temperature for 18h under Ar in the dark. Sat. aq. NH_4_Cl solution (1.0 mL) was added, the mixture was extracted with ethyl acetate (3 × 5 mL), the combined organic layers were washed with LiCl 10% (3 × 5 mL), dried over Na_2_SO_4_, and evaporated *in vacuo*. The crude product was purified by flash chromatography on a 4 g silica cartridge, using EA/Cy (0–40%), as eluent. The obtained compound was taken up in ethyl acetate (5 mL) and washed with water (4 × 3 mL), organic layer was dried over Na_2_SO_4_, evaporated *in vacuo,* to afford **14** (17.0 mg, 56%), as a white powder. UPLC-MS (Apolar method): *R*_t_ = 2.10 min. MS (ESI+) calcd. for C_36_H_30_F_5_N_6_O_5_ [M + H]^+^ 721.2; obsd. 721.3. ^1^H NMR (400 MHz, DMSO-*d*_6_) δ 10.40 (s, 1H), 8.21 (t, *J* = 5.6 Hz, 1H), 8.11 (app t, *J* = 2.0 Hz, 1H), 7.93 (dt, *J* = 7.9, 1.3 Hz, 1H), 7.83–7.78 (m, 1H), 7.77–7.74 (m, 1H), 7.68–7.63 (m, 2H), 7.56 (t, *J* = 7.9 Hz, 1H), 7.37–7.33 (m, 2H), 6.97–6.91 (m, 2H), 5.82–5.76 (m, 1H), 3.10 (q, *J* = 6.9 Hz, 2H), 2.86–2.78 (m, 2H), 2.68–2.58 (m, 1H), 2.14–2.06 (m, 1H), 2.00 (td, *J* = 7.4, 2.7 Hz, 2H), 1.94- 1.81 (m, 3H), 1.61 (t, *J* = 7.3 Hz, 4H).

#### *N*-(2,2-Difluorobenzo[*d*][1,3]dioxol-5-yl)-3-((*S*)-7-(4-((2-(3-(2-(1-(18-((4*S*,5*R*)-5-methyl-2-oxoimidazolidin-4-yl)-13-oxo-3,6,9-trioxa-12-azaoctadecyl)-1*H*-1,2,3-triazol-4-yl)ethyl)-3*H*-diazirin-3-yl)ethyl)carbamoyl)phenoxy)-3-(trifluoromethyl)-4,5,6,7-tetrahydro-1*H*-indazol-1-yl)benzamide (**PAP_N1 DS-biot**)

A freshly prepared 0.1 M solution of desthiobiotin-PEG3-azide in dry THF (300 μL, 0.03 mmol) was added to **14** (21.6 mg, 0.03 mmol) under Ar atmosphere in the dark. *Tert*-butanol (185 μL), water (185 μL), a freshly prepared 1.0 M solution of CuSO_4_**·** 5H_2_O in water (6 μL, 0.006 mmol), and a freshly prepared 2.0 M solution of sodium ascorbate in water (1.8 μL, 0.0036 mmol) were sequentially added, and the reaction was stirred at room temperature for 18 h in the dark. Water (2.0 mL) was added, and the mixture was extracted with ethyl acetate (3 × 5.0 mL), before the combined organic layers were washed with brine (2 × 5 mL), dried over Na_2_SO_4_, and evaporated *in vacuo.* The crude product was purified by flash chromatography on a 4 g silica cartridge, using 10% MeOH in DCM/DCM (0–68%), as eluent. The obtained product was lyophilized from ACN:water (1:3) to afford **PAP_N1 DS-biot** (10.1 mg, 30%), as a white solid. UPLC-MS (Generic method): *R*_t_ = 5.15 min. MS (ESI+) calcd. for C_54_H_64_F_5_N_12_O_10_ [M + H]^+^ 1135.48; obsd. 1135.42. HRMS (ESI+) *m*/*z*: calcd. for C_54_H_64_F_5_N_12_O_10_ [M + H]^+^, 1135.4783; found 1135.4796. ^1^H NMR (400 MHz, DMSO-*d*_6_) δ 10.46 (s, 1H), 8.24 (t, *J* = 5.6 Hz, 1H), 8.12 (app t, *J* = 2.0 Hz, 1H), 7.94 (dt, *J* = 7.9, 1.3 Hz, 1H), 7.83–7.75 (m, 4H), 7.69–7.63 (m, 2H), 7.56 (t, *J* = 7.9 Hz, 1H), 7.39–7.32 (m, 2H), 6.97–6.91 (m, 2H), 6.29 (s, 1H), 6.11 (s, 1H), 5.82–5.77 (m, 1H), 4.46 (t, *J* = 5.3 Hz, 2H), 3.77 (t, *J* = 5.3 Hz, 2H), 3.59 (p, *J* = 6.4 Hz, 1H), 3.52–3.42 (m, 8H), 3.20–3.06 (m, 4H), 2.86–2.76 (m, 1H), 2.67–2.57 (m, 1H), 2.46–2.39 (m, 2H), 2.15–2.00 (m, 3H), 1.95–1.73 (m, 5H), 1.59 (t, *J* = 7.3 Hz, 2H), 1.46 (p, *J* = 7.3 Hz, 2H), 1.39–1.06 (m, 8H), 0.94 (d, *J* = 6.4 Hz, 3H). ^19^F (376 MHz, DMSO-*d*_6_) δ −49.00, −60.31.

#### *N*-Methyl-3-((*S*)-7-(4-(1-(18-((4*S*,5*R*)-5-methyl-2-oxoimidazolidin-4-yl)-13-oxo-3,6,9-trioxa-12-azaoctadecyl)-1*H*-1,2,3-triazol-4-yl)phenoxy)-3-(trifluoromethyl)-4,5,6,7-tetrahydro-1*H*-indazol-1-yl)-*N*-(4-(3-(trifluoromethyl)-3*H*-diazirin-3-yl)phenyl)benzamide (**PAP_2 DS-biot**)

A freshly prepared 0.1 M solution of desthiobiotin-PEG3-azide in dry THF (300 μL, 0.03 mmol) was added to **PAP_2** (18.4 mg, 0.03 mmol) under Ar atmosphere in the dark. *Tert*-butanol (181 μL), water (181 μL), a freshly prepared 1.0 M solution of CuSO_4_**·** 5H_2_O in water (1.8 μL, 0.002 mmol), and a freshly prepared 2.0 M solution of sodium ascorbate in water (1.5 μL, 0.003 mmol) were sequentially added, and the reaction was stirred at room temperature for 18 h in the dark. Additional amounts of a freshly prepared 1.0 M solution of CuSO_4_**·** 5H_2_O in water (4 × 1.8 μL, 0.008 mmol) and of a freshly prepared 2.0 M solution of sodium ascorbate in water (4 × 1.5 μL, 0.012 mmol) were added within 6 h, after which the reaction was worked-up. Water (2.0 mL) was added, and the mixture was extracted with ethyl acetate (3 × 5.0 mL), before the combined organic layers were washed with brine (2 × 5 mL), dried over Na_2_SO_4_, and evaporated *in vacuo.* The crude product was purified on silica gel by manual flash chromatography, using 2% MeOH in DCM, as eluent. The obtained product was lyophilized from ACN:water (1:3) to afford **PAP_2 DS-biot** (12.6 mg, 41%), as a white-yellow solid. UPLC-MS (Apolar method): *R*_t_ = 3.04 min. MS (ESI+) calcd. for C_50_H_58_F_6_N_11_O_7_ [M + H]^+^ 1038.44; obsd. 1038.06. HRMS (ESI+) *m*/*z*: calcd. for C_50_H_58_F_6_N_11_O_7_ [M + H]^+^, 1038.4419; found 1038.4427. ^1^H NMR (600 MHz, DMSO-*d*_6_) δ 8.44–8.41 (m, 1H), 7.83–7.79 (m, 1H), 7.77–7.73 (m, 2H), 7.60–7.56 (m, 1H), 7.54 (d, *J* = 7.8 Hz, 1H), 7.29 (t, *J* = 7.9 Hz, 1H), 7.21–7.16 (m, 1H), 7.12 (d, *J* = 8.3 Hz, 2H), 7.01 (dd, *J* = 7.7, 3.5 Hz, 3H), 6.30 (s, 1H), 6.12 (s, 1H), 5.71–5.66 (m, 1H), 4.54 (t, *J* = 5.2 Hz, 2H), 3.84 (t, *J* = 5.2 Hz, 2H), 3.61–3.41 (m, 11H), 3.23–3.12 (m, 5H), 2.82–2.75 (m, 1H), 2.62–2.55 (m, 1H), 2.17–2.09 (m, 1H) 2.03 (t, *J* = 7.4 Hz, 2H), 1.88–1.78 (m, 3H), 1.45 (p, *J* = 7.4 Hz, 2H), 1.36–1.11 (m, 11H), 0.93 (d, *J* = 6.4 Hz, 3H). ^19^F (376 MHz, DMSO-*d*_6_) δ −60–41, - 64.70.

#### 3-((*S*)-7-(4-(1-(18-((4*S*,5*R*)-5-Methyl-2-oxoimidazolidin-4-yl)-13-oxo-3,6,9-trioxa-12-azaoctadecyl)-1*H*-1,2,3-triazol-4-yl)phenoxy)-3-(trifluoromethyl)-4,5,6,7-tetrahydro-1*H*-indazol-1-yl)-*N*-(4-(3-(trifluoromethyl)-3*H*-diazirin-3-yl)phenyl)benzamide (**PAP_N2 DS-biot**)

A freshly prepared 0.1 M solution of desthiobiotin-PEG3-azide in dry THF (300 μL, 0.03 mmol) was added to **11** (18.4 mg, 0.03 mmol) under Ar atmosphere in the dark. *Tert*-butanol (185 μL), water (185 μL), a freshly prepared 1.0 M solution of CuSO_4_**·** 5H_2_O in water (1.8 μL, 0.002 mmol), and a freshly prepared 2.0 M solution of sodium ascorbate in water (1.5 μL, 0.003 mmol) were sequentially added, and the reaction was stirred at room temperature for 18 h in the dark. Additional amounts of a freshly prepared 1.0 M solution of CuSO_4_**·** 5H_2_O in water (4 × 1.8 μL, 0.008 mmol) and of a freshly prepared 2.0 M solution of sodium ascorbate in water (4 × 1.5 μL, 0.012 mmol) were added within 6.5 h, after which the reaction was worked-up. Water (2.0 mL) was added, and the mixture was extracted with ethyl acetate (3 × 5.0 mL), before the combined organic layers were washed with brine (2 × 5 mL), dried over Na_2_SO_4_, and evaporated *in vacuo.* The crude product was purified on silica gel by manual flash chromatography, using 5% MeOH in DCM, as eluent. The obtained product was lyophilized from ACN:water (1:3) to afford **PAP_N2 DS-biot** (15.1 mg, 49%), as a white-yellow solid. UPLC-MS (Apolar method): *R*_t_ = 3.21 min. MS (ESI+) calcd. for C_49_H_56_F_6_N_11_O_7_ [M + H]^+^ 1024.43; obsd. 1024.32. HRMS (ESI+) *m*/*z*: calcd. for C_49_H_56_F_6_N_11_O_7_ [M + H]^+^, 1024.4263; found 1024.4290. ^1^H NMR (400 MHz, DMSO-*d*_6_) δ 10.48 (s, 1H), 8.34–8.30 (m, 1H), 8.20–8.15 (m, 1H), 7.98–7.93 (m, 1H), 7.86–7.75 (m, 3H), 7.63–7.54 (m, 3H), 7.20 (d, *J* = 8.4 Hz, 2H), 6.98–6.92 (m, 2H), 6.28 (s, 1H), 6.10 (s, 1H), 5.76–5.71 (m, 1H), 4.54 (t, *J* = 5.2 Hz, 2H), 3.85 (t, *J* = 5.2 Hz, 2H), 3.62–3.41 (m, 9H), 3.14 (q, *J* = 5.8 Hz, 2H), 2.87–2.78 (m, 1H), 2.69–2.57 (m, 1H), 2.18–2.08 (m, 1H), 2.03 (t, *J* = 7.4 Hz, 2H), 1.94–1.82 (m, 3H), 1.44 (q, *J* = 7.3 Hz, 2H), 1.38–1.07 (m, 7H), 0.93 (d, *J* = 6.4 Hz, 3H). ^19^F (376 MHz, DMSO-*d*_6_) δ −60.29, −64.68.

### Biology

#### Cell Culture

Wild type (wt)-CFTR or F508del-CFTR-overexpressing CFBE41o- cell lines^61^ were cultured in minimum essential medium (MEM). HEK293 (CRL-1573, ATCC) cells were cultured in Dulbecco’s Modified Eagle Medium (DMEM, D6546 Sigma-Aldrich). Both media were supplemented with 10% fetal bovine serum (FBS, EUS5000L Euroclone), 2 mM (*L*)-glutamine (G7513, Sigma-Aldrich), penicillin (100 U/mL), and streptomycin (100 μg/mL) (P4333, Sigma-Aldrich). Puromycin (2 μg/mL) (P8833, Sigma-Aldrich) was added to wt-CFTR or F508del-CFTR CFBE41o- cell lines to maintain the recombinant genes’ expression. Cells were grown at 37 °C and 5% CO_2_ and passaged every 3–5 days.

#### In-Cell Photo-Affinity Labeling

Wt-CFTR or F508del-CFTR CFBE41o- cells were plated at 2–2.5 × 10^6^ cells/plate in 6 cm diameter dishes. The day after, sodium butyrate (5–2.5 mM) was added for 24 h; next, the medium was exchanged with fresh one supplemented with DMSO (0.25%) (276855, Sigma-Aldrich) or probe (1 μM). For competition experiments, a 25-fold excess of **ARN23765** (25 μM) was added 30 min before probe’s addition. For the analysis of “corrected” F508del-CFTR, F508del-CFTR CFBE41o- cells were kept at 27 °C for 24 h before adding the probes. Probes were incubated with cells for 2 h, then the medium was removed, and plates were washed twice with phosphate buffer saline (PBS). Cells were placed on ice and irradiated for 5 min at a wavelength of 365 nm using a 100-W UV-lamp (95–0127–02 model B100-AP, Analytik Jena). Cells were harvested with trypsin (T4299, Sigma-Aldrich), washed with PBS (D8537, Sigma-Aldrich), and pellets were stored at −80 °C until used, centrifuged at 300 × g and washed twice with PBS (D8537, Sigma-Aldrich). Pellets from in-cell photoaffinity labeling with alkyne-probes (**PAP_1** and **PAP_2**) were lysed in PBS plus protease inhibitor cocktail (78429, Thermo Scientific) by sonication. The suspension was centrifuged at 800 × g for 20 min at 4 °C to discard nuclei and cell debris. The total extracted proteins were quantified with the bicinchoninic acid (BCA) protein assay (23225, Thermo Scientific) and subjected to CuAAC before proceeding with pull-down. Pellets from in-cell photoaffinity labeling with desthio-biotinylated (DS-biot) probes (**PAP_1 DS-biot**, **PAP_2 DS-biot**, **PAP_N1 DS-biot**, **PAP_N2 DS-biot**) were lysed with a modified RIPA buffer [50 mM tris-HCl pH 8, 150 mM NaCl, 0.5% Triton-X100, 0.2% SDS, 1 mM ethylenediaminetetraacetic acid (EDTA)] supplemented with protease inhibitor cocktail and centrifuged at 10.000× g for 10 min. The protein content in the supernatant was quantified by BCA protein assay. Protein lysate (200 μg) was diluted to 800 μL and the Triton-X100 percentage was reduced to 0.2% before proceeding to pull-down.

#### Copper-Catalyzed Azide–Alkyne Cycloaddition (CuAAC)

Copper-catalyzed azide–alkyne cycloaddition (CuAAC) was performed according to a previously described protocol.^62^ Briefly, to the protein preparation (250 μL at 1 mg/mL protein concentration) the following reagents were added at the indicated final concentrations: 100 μM 5/6-TAMRA-azide-desthiobiotin (CLK-1110–5, Jena Bioscience), 1.0 mM *tris*-(2-carbossietil)phosphine (TCEP, C4706, Sigma-Aldrich), 100 μM *tris-*[(1-benzyl-1H-1,2,3-triazol-4-yl)methyl]amine (TBTA, 678937, Sigma-Aldrich), 1.0 mM CuSO_4_.5H_2_O (Sigma), and 0.8% sodium dodecyl sulfate (SDS). TBTA was first dissolved in DMSO at 83.5 mM and then diluted with four volumes of *tert*-butanol. The reaction was mixed by vortexing and incubated for 1 h at 25 °C. After the reaction time, proteins were precipitated with 5 volumes of cold acetone overnight at −20 °C. Samples were centrifuged for 20 min at 20.000× g, and the pellets were washed with methanol, air-dried, and finally resuspended with 600 μL of 1% SDS. SDS concentration was then diluted to 0.2% with PBS before proceeding to pull-down.

#### Pull-Down

High capacity streptavidin agarose (20359, Thermo Scientific) (25 μL) was added to PAP-labeled cell extracts for 1 h at room temperature under gentle rotation. The streptavidin beads were collected by centrifugation and washed four times with lysis buffer. Resin-bound proteins were eluted with 45 μL of 5 mM biotin (B4501, Sigma-Aldrich) for 30 min at room temperature and analyzed with Western blot for CFTR expression. Input samples (4 and 8 μg for wt-CFTR and F508del-CFTR CFBE41o-, respectively) were collected immediately before incubation with streptavidin resin. Input and pulled-down samples were analyzed with WB as described in the supplementary methods.

#### CFTR Fragment Stability Analysis

Constructs coding for MSD1 (residues 1–380), A52-tagged MSD2 (residues 837–1196) and MSD2-NBD2 (residues 850–1480) were transfected in HEK293 cells using JetPEI reagent (101000053, Polyplus Technology). After 18 h transfection, cells were treated for 24 h with either 0.2% DMSO, VX-661 (3 μM), **ARN23765** (10 nM) or corr-4a (10 μM). Cell lysates were analyzed by WB, as described in the dedicated paragraph.

#### Cycloheximide Chase Assay

HEK293 cells were transfected with MSD1- or MSD2-fragments as previously described.^39^ After 18 h, cells were treated with either 0.2% DMSO or **ARN23765** (10 nM). Following 24 h treatment, the protein synthesis was stopped by addition of a medium containing 0.5 mg/mL cycloheximide and either DMSO (0.2%) or **ARN23765** (10 nM). The cells were incubated at 37 °C and lysed at various time periods (0–6 h). The whole cell extracts were then analyzed by WB, as described in the supplementary methods of the Supporting Information.

#### Protein Maturation Assay

HEK293 cells were plated in 24-wells at around 80% of confluence and transfected with constructs coding for F508del-CFTR or mutants using JetPEI reagent (101000053, Polyplus Technology) following the manufacturer’s instructions. After 24 h, the correctors were added for a further 24 h. Correctors were used at the following concentrations: **ARN23765** (10 nM), VX-445 (3 μM), and 3151 (10 μM). For binding site analysis experiments, the cells were kept either at 37 °C (Figure S8) or moved to 30 °C (Figure 9) during incubation with the selected correctors. VX-445 and corrector 3151 were mixed together as a control for maximal protein correction. DMSO (0.2%) was added to nontreated controls. After treatment, cells were lysed in RIPA buffer [50 mM *tris*-HCl pH 8, 150 mM NaCl, 1% NP40, 0.5% sodium deoxycolate, 0.1% SDS, 2.0 mM EDTA] and lysates were analyzed with WB, as described in the supplementary methods of the Supporting Information.

### Computational Chemistry

#### Molecular Docking Calculations

Docking studies were performed on a F508del-CFTR protein model obtained as previously described.^18^ A series of cross docking calculations were performed on the protein complexes at PDB-ID: 8EIG, 8EIQ, 8EIO.^18^ Among them, 8EIQ was chosen for all the computations. The proposed binding modes for each former lead were compared to the crystal pose and were considered as the reference point for the subsequent optimization strategy. Prior to proceeding with the docking of the designed compounds, the ability of *Glide* docking software (Schrödinger Release 2022–2: Glide, Schrödinger, LLC, New York, NY, 2022)^63^ in reproducing the previous data was evaluated. **ARN23765** and compound **2** were manually designed and then prepared for the subsequent studies using the LigPrep utility of the Schrödinger suite, selecting the specific stereoisomers and evaluating all the possible tautomers and ionization states (pH 7.0 ± 2.0) by means of the Epik software. The cryo-EM structure used for the calculations/simulations was retro-mutated from the original PDB file. The partially resolved R-domain was not completed and then removed from the gained model. All the gaps were fulfilled, in agreement with the default procedure of the Protein preparation wizard tool from Maestro. Docking studies were run using the Glide SP scoring function, an enhanced sampling for the generation of ligand conformations, leaving the rest of parameters to their default values. Only the first binding mode for each ligand was visually inspected and evaluated.

#### In-Silico Single Point Mutations

The changes in binding affinity (Δaffinity)^45^ were calculated through Binding Affinity Prediction and Residue Scanning tool from Schrodinger. The change in the binding affinity of the protein due to the mutation was calculated from the two individual binding energies, which can be represented as follows:
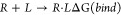

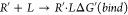


*L* is the ligand/compound in the parent protein, *R* is the wild type protein and *R’* is the mutated protein. *R+L* and *R’+L* represent the separated protein and ligand/compound. *R·L* and *R’·L* represent the protein bound to the ligand/compound. The change in binding affinity is represented by
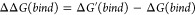


The calculations were done with Prime MM-GBSA, which uses an implicit (continuum) solvation model. A negative value indicates that the mutant binds better than the parent protein to the ligand/compound.^64,65^ The binding energy calculations were performed with Molecular Mechanics-Generalized Born Surface Area (MM-GBSA) protocol using VSGB solvation model and OPLS4 Force field.

#### Molecular Dynamics Simulations

The molecular complexes, derived from docking calculations, were embedded in a 1-palmitoyl-2-oleoyl-*sn*-glycero-3-phosphatidylcholine (POPC model) membrane, solvated with water molecules (TIP3P model) and ionized with KCl at 0.15M. The Force Field used for protein, water and ions was Amber19SB, whereas Lipid21 was used for lipids. **ARN23765** in each complex was parametrized with AnteChamber, adopting AM1BCC as semiempirical calculations to derives the ligand charges. The entire procedure was conducted using CHARMM-GUI server.^66^ Each system consisted of ca. 180,000 atoms and the preproduction procedure entailed 10,000 steps of minimization using a mixed steepest descent and conjugate gradient protocol. The equilibration procedure comprised the system heating up to 303.15 K and pressurized up to 1 atm. The complexes equilibration was performed for 300 ns, using a protocol of stepwise decreasing restrained simulation to guarantee the maintaining of the channel conformation and the ligands binding poses before the production steps. The Molecular Dynamics (MD) simulations in production were conducted without any restraint applied to protein, ligand or cofactor, adopting an NPT ensemble for both equilibration and production. The temperature coupling algorithm used was V-Rescale,^67^ and the barostat algorithm used was C-Rescale.^68^ Each complex was simulated for 0.5 μs at 2 fs as time step. The MD simulations were conducted using GROMACS23 engine and relative tools for analysis.^69^ The dynamical ligand contact analyses were performed using PyContact protocol adopting a distance cutoff of 2.5 Å.^70^

### Statistical Analysis

All the data are represented as mean ± SD of at least three independent replicates, the exact number of replicates is reported in Figure legends. GraphPad 10.0 software was used for all statistical analyses. Unpaired two-tailed *t* test, one-way or two-way ANOVA were conducted as appropriate and significance was assigned with a *p* ≤ 0.05. Multiple comparisons were assessed using Bonferroni’s, Dunnett’s or Tukey’s posthoc tests. All statistical analyses, posthoc tests and assigned *p* values are detailed in the Figure legends.

## Supporting Information Available

^1^H, ^1^H–^13^C HSQC and ^19^F NMR spectral data and UPLC/MS traces for all final compounds; additional figures (Figures S1–S10), biology methods, and tables (Tables S1–S3) (PDF)
Video of MD analyses of **ARN23765** and compound **2** in corrector type I binding site, and **ARN23765** in K68I mutant corrector type I binding site (MPG)
PDB file for the docking calculations of **ARN23765** to VX-445 (PDB)
PDB file for the docking calculations of **ARN23765** to VX-661 (PDB)
PDB file for the docking calculations of **ARN23765** to VX-770 (PDB)
PDB file for the docking calculations of compound **2** to VX-445(PDB)
PDB file for the docking calculations of compound **2** to VX-661(PDB)
PDB file for the docking calculations of compound **2** to VX-770 PDB)
SMILES (CSV)

## Supplementary Material

jm4c02654_si_001.pdf

jm4c02654_si_002.mpg

jm4c02654_si_003.pdb

jm4c02654_si_004.pdb

jm4c02654_si_005.pdb

jm4c02654_si_006.pdb

jm4c02654_si_007.pdb

jm4c02654_si_008.pdb

## ABBREVIATIONS

CF: cystic fibrosis
CFTR: cystic fibrosis transmembrane conductance regulator
CFBE41o-: cystic fibrosis bronchial epithelial 41o-
CuAAC: copper-catalyzed azide–alkyne cycloaddition
DIAD: diisopropylazodicarboxylate
DIPEA: N,N-diisopropylethylamine
DS-biot: desthio-biotin
F508del: deletion of phenylalanine 508
FLIPR: fluorometric imaging plate reader
HATU: 1-[bis(dimethylamino)methylene]-1H-1,2,3-triazolo[4,5-*b*]pyridinium 3-oxid hexafluorophosphate
HEK293: human embryonic kidney 293
HS-YFP: halide-sensitive yellow fluorescent protein
ICL: intracellular loop
MSD: membrane-spanning domain
NBD: nucleotide-binding domain
PAL: photoaffinity labeling
PAP: photoaffinity probe
RMSD: root-mean-square deviation
TM: transmembrane
YFP: yellow fluorescent protein
