# Supplementary material for: Target Identification with Live-Cell Photoaffinity Labeling and Mechanism of Action Elucidation of ARN23765, a Highly Potent CFTR Corrector
Source: J Med Chem. 2025 Feb 10;68(4):4596–618. doi: 10.1021/acs.jmedchem.4c02654 (PMC11873939; doi:10.1021/acs.jmedchem.4c02654)
Supplement: Supplementary file 1 — jm4c02654_si_001.pdf [file jm4c02654_si_001.pdf]

Supporting Information

Target identification with live-cell photo-affinity labelling and mechanism of action elucidation of ARN23765, a highly potent CFTR corrector

Elisa Romeo[1,£,]; Francesco Saccoliti[2,$,£]; Riccardo Ocello[3,4,£]; Angela Andonaia[2]; Caterina Allegretta[5]; Cristina Pastorino[6]; Nicoletta Pedemonte[6]; Federico Falchi[3,4]; Onofrio Laselva[5]; Tiziano Bandiera[2]; Fabio Bertozzi[2,*]*

[1]Structural Biophysics Facility, Istituto Italiano di Tecnologia (IIT), Genova, ITALY; [2]D3-PharmaChemistry, Istituto Italiano di Tecnologia (IIT), Genova, ITALY; [3]Department of Pharmacy and Biotechnology, University of Bologna, Bologna, ITALY; [4]Computational and Chemical Biology, Istituto Italiano di Tecnologia (IIT), Genova, ITALY; [5]Department of Clinical and Experimental Medicine, University of Foggia, Foggia, ITALY; [6]U.O.C. Genetica Medica, Istituto Giannina Gaslini (IGG), Genova, ITALY.

[$]Current address: Department of Life Science, Health, and Health Professions, Link Campus University, Rome, ITALY

[£]These authors contributed equally to this work

*Correspondence. fabio.bertozzi@iit.it, http://orcid.org/0000-0001-7434-3688 (F.B.); elisa.romeo@iit.it (E.R.)

TABLE OF CONTENTS

1. ***SUPPLEMENTARY FIGURES*** S.3
   - *Figure S1.* Structures of investigated final compounds.
   - *Figure S2.* **ARN23765**-derived analogues and PAPs testing with the HS-YFP assay in F508del-CFTR CFBE41o- cells.
   - *Figure S3.* PAL experiments with terminal alkyne-substituted PAPs.
   - *Figure S4.* Quantification analysis of PAL competitive experiments shown in Figure 2 (B-C).
   - *Figure S5.* Binding mode comparison between **ARN23765** and former ligand after docking calculations, in surface representations.
   - *Figure S6*. Molecular docking analyses of compound **2** in the described CFTR binding sites.
   - *Figure S7.* MD simulation of **ARN23765** in K68I/F508del-CFTR.
   - *Figure S8.* In-cell mutagenesis studies at 37°C.
   - *Figure S9.* Representative traces of CFTR channel activity evaluated by FLIPR assay.
   - *Figure S10.* 2D-Binding mode comparison between ARN23765 and former ligand after docking calculations.
2. ***SUPPLEMENTARY BIOLOGY METHODS*** S.13
   - HS-YFP-based assay for CFTR activity
   - Western blot
   - CFTR Channel function analysis by fluorometric imaging plate reader functional (FLIPR) Assay
   - Site-directed mutagenesis
3. ***SUPPLEMENTARY TABLES*** S.15
   - *Table S1:* Molecular docking scores of VX-809, VX-445, VX-770, **ARN23765** and compound **2** in F508del-CFTR (PDB-ID: 8EIQ).
   - *Table S2:* Binding free energies and non-covalent contributions for VX-809, VX-445, VX-770, **ARN23765** and compound **2** in F508del-CFTR.
   - *Table S3:* Primers used for mutagenesis
4. ***REPRESENTATIVE ^{1}H, ^{1}H-^{13}C HSQC, ^{19}F NMR SPECTRA OF FINAL COMPOUNDS*** S.17
5. ***LC-MS ANALYSES OF FINAL COMPOUNDS*** S.34
6. ***REFERENCES*** S.43

1. *SUPPLEMENTARY FIGURES*

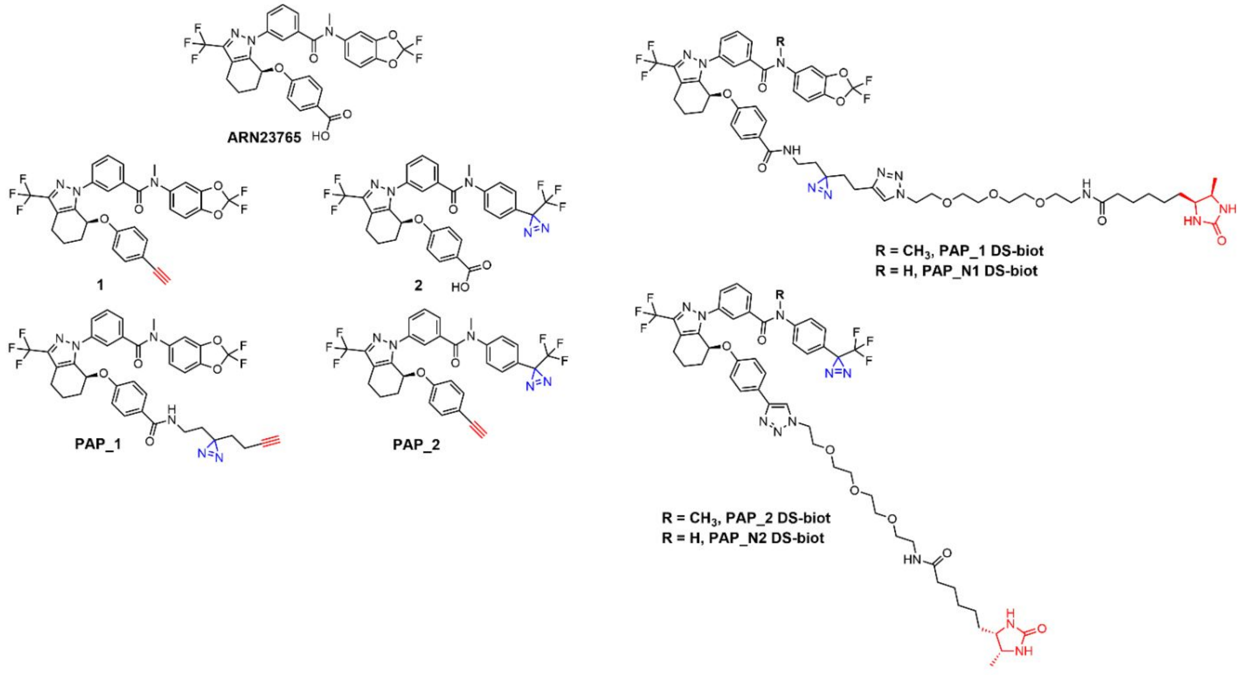

Figure S1. *Structures of investigated final compounds.* **ARN23765**, its close analogues (**1** and **2**), structurally derived alkyne-substituted (**PAP_1** and **PAP_2**) and desthio-biotinylated (**PAP_1 DS-biot**, **PAP_N1 DS-biot**, **PAP_2 DS-biot**, and **PAP_N2 DS-biot**) probes.

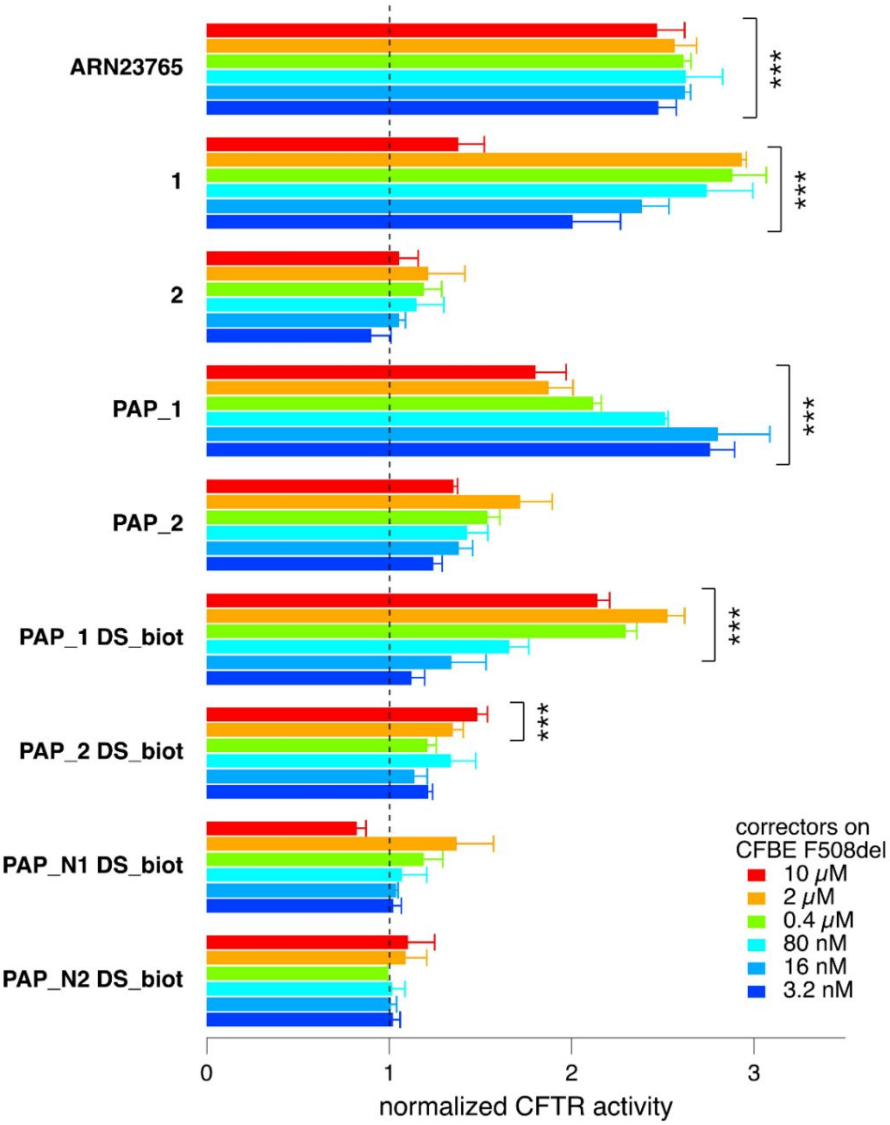

Figure S2. ***ARN23765**-derived analogues and PAPs testing with the HS-YFP assay in F508del-CFTR CFBE41o- cells.*[1] Bar graph showing CFTR activity (mean ± SD; n=3) in F508del-CFTR CFBE41o- cells following 24 h treatment with **ARN23765**-derived analogues and probes at the indicated concentrations, determined by the HS-YFP assay. Values were normalized to DMSO-incubated cells. One-way ANOVA (Dunnett's multiple comparisons test, compound *vs.* DMSO: ***, $p < 0.001$).

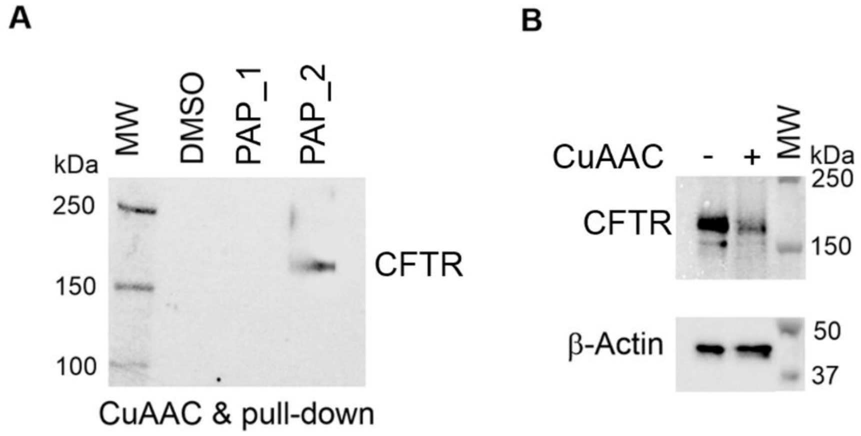

Figure S3. *PAL experiments with terminal alkyne-substituted PAPs.* (A) Western blot analysis of CFTR in biotin-streptavidin pulled-down protein fraction from in-cell PAL experiments in wt-CFTR CFBE41o-. Cells were incubated with **PAP_1** or **PAP_2** (2.5 μM) and irradiated under UV light. DMSO (0.25%) was used as negative control. After cell lysis, a DS-biot tag was added via CuAAC and protein-probe adducts were pulled down with streptavidin. (B) Western blot analysis of CFTR expression in wt-CFTR CFBE41o- lysate before (-) and after (+) the implementation of CuAAC protocol. Beta-actin was detected for comparison. MW: molecular weight marker. The anti-CFTR antibody 596 (provided by the American CFF) was used to detect CFTR. Images are representative of three different experiments.

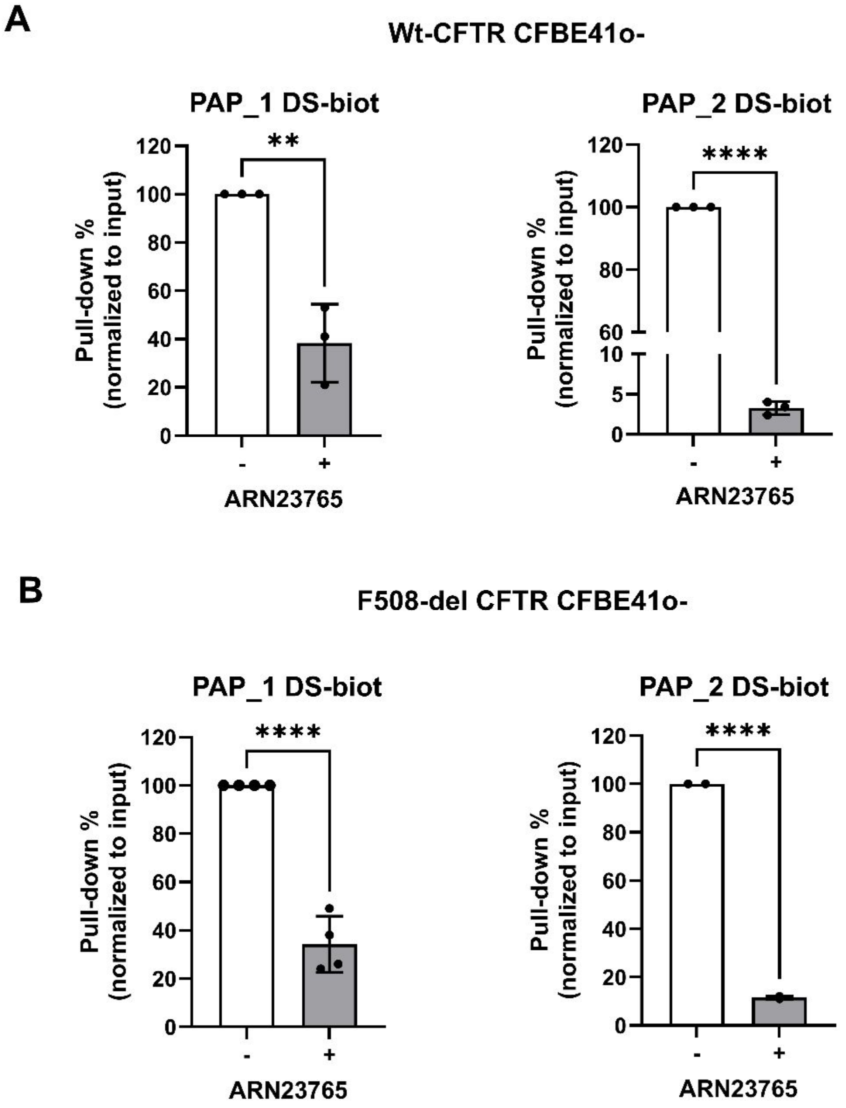

Figure S4. *Quantification analysis of PAL competitive experiments shown in Figure 2 (B-C).* Wt- (A) or F508del-CFTR (B) CFBE41o- cells were either pre-incubated (+) or not (-) with **ARN23765** (25 μM, 30 min at 37 °C) and then **PAP_1 DS-biot** (1 μM) or **PAP_2 DS-biot** (1 μM) were added for 2 h at 37 °C. Input and pull-down samples were analyzed with WB, as shown in Figure 2 (B-C). Pull-down band intensities obtained were normalized to the corresponding input signals and expressed as a percentage of full signals obtained in the absence of **ARN23765**. Mean ± SD of 2-4 independent experiments are reported. Unpaired t-test analysis: ** $p<0.01$, **** $p< 0.0001$.

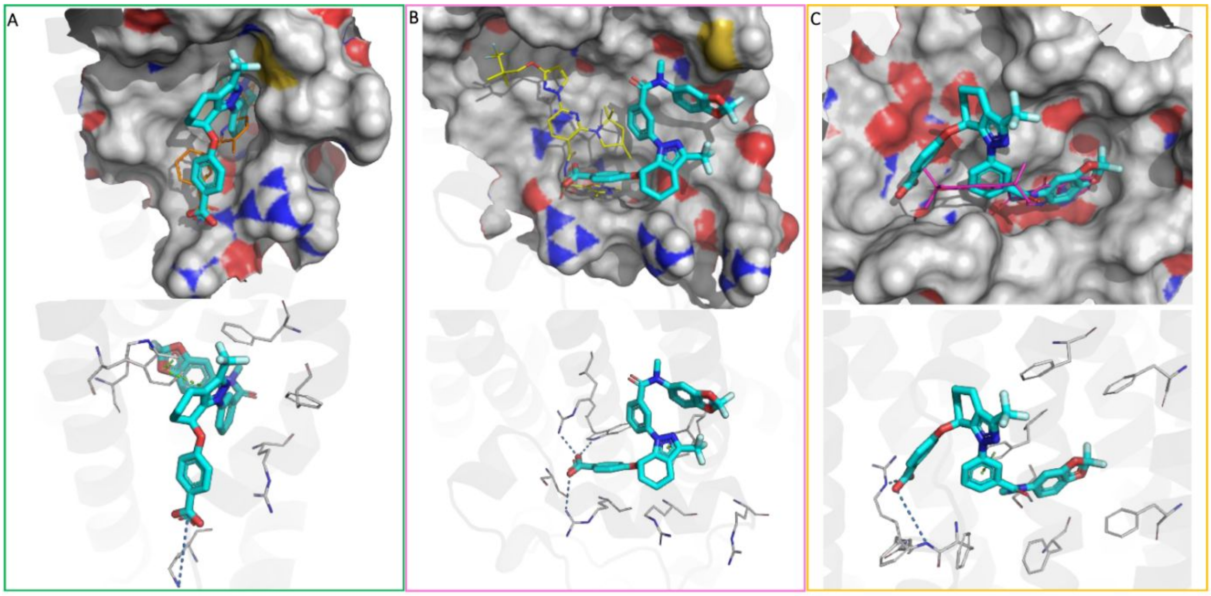

Figure S5. *Binding mode comparison between **ARN23765** and former ligand after docking calculations, in surface representations.* Docking of **ARN23765** in VX-809 (*A, thin stick orange molecule*), VX-445 (*B, thin stick yellow molecule*), VX-770 (*C, thin stick pink molecule*) binding sites, and the corresponding stick representations. The superimposition of ligands allows conformational comparison between the adopted binding poses.

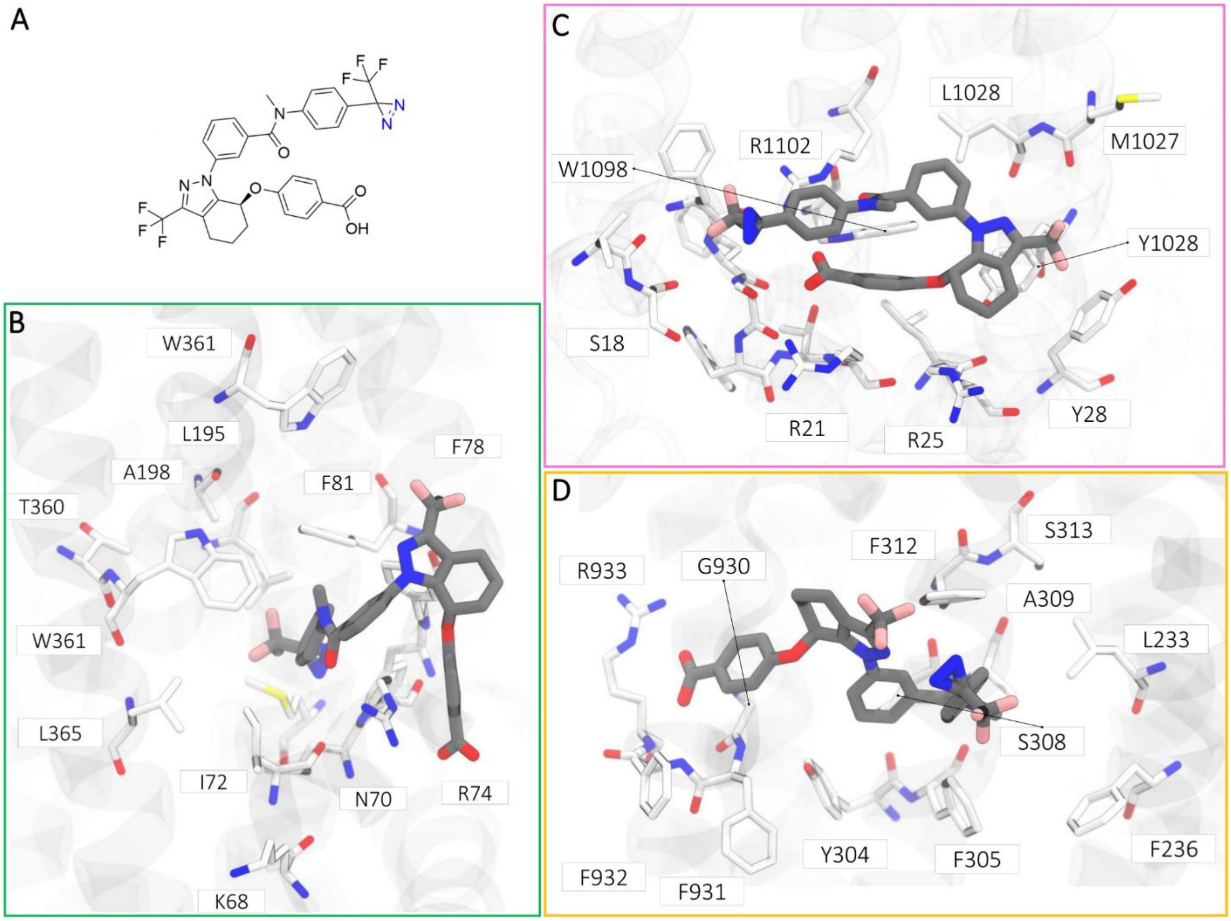

Figure S6. *Molecular docking analyses of compound* ***2*** *in the described CFTR binding sites*[2]. (A) Structure of compound **2**. (B-D) Docking poses of compound **2** in VX-809 (*B, green frame*), VX-445 (*C, pink frame*), VX-770 (*D, yellow frame*) binding site.

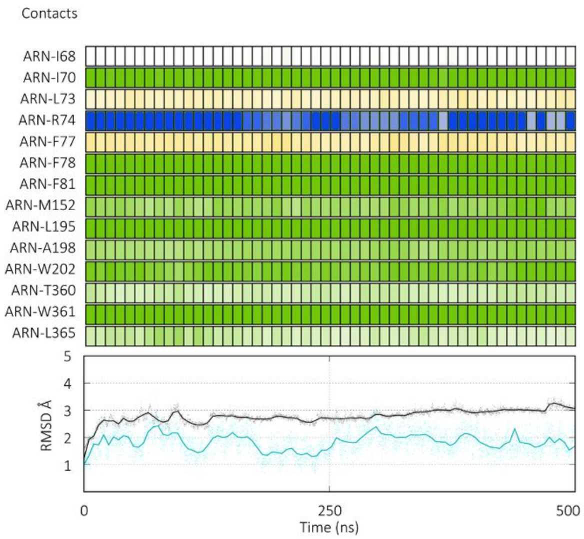

Figure S7. *MD simulation of **ARN23765** in K68I/F508del-CFTR. Top*: Time frames of **ARN23765** interactions within K68I/F508del-CFTR binding site during the 0.5 µs long MD simulation, in which colored boxes are representative of the interactions' engagement between **ARN23765** and the amino acid residues, through H-bond (blue), side chain apolar (green) and backbone (yellow). The contact cut-off between **ARN23765** and the residues was set at 2.5 Å and the color intensity is proportional to the contact's distance. White boxes stand for lost interaction, *i.e.,* over 6 Å; *bottom*: RMSD analysis for K68I/F508del-CFTR (black trace) and **ARN23765** (cyan trace) during the entire trajectory simulation. The protein conformational stability was calculated by measuring the atomic coordinates average displacement between each timestep and a reference structure. In general, the smaller the deviation, the more stable the protein structure. The equilibrated F508del-CFTR was used as a reference.

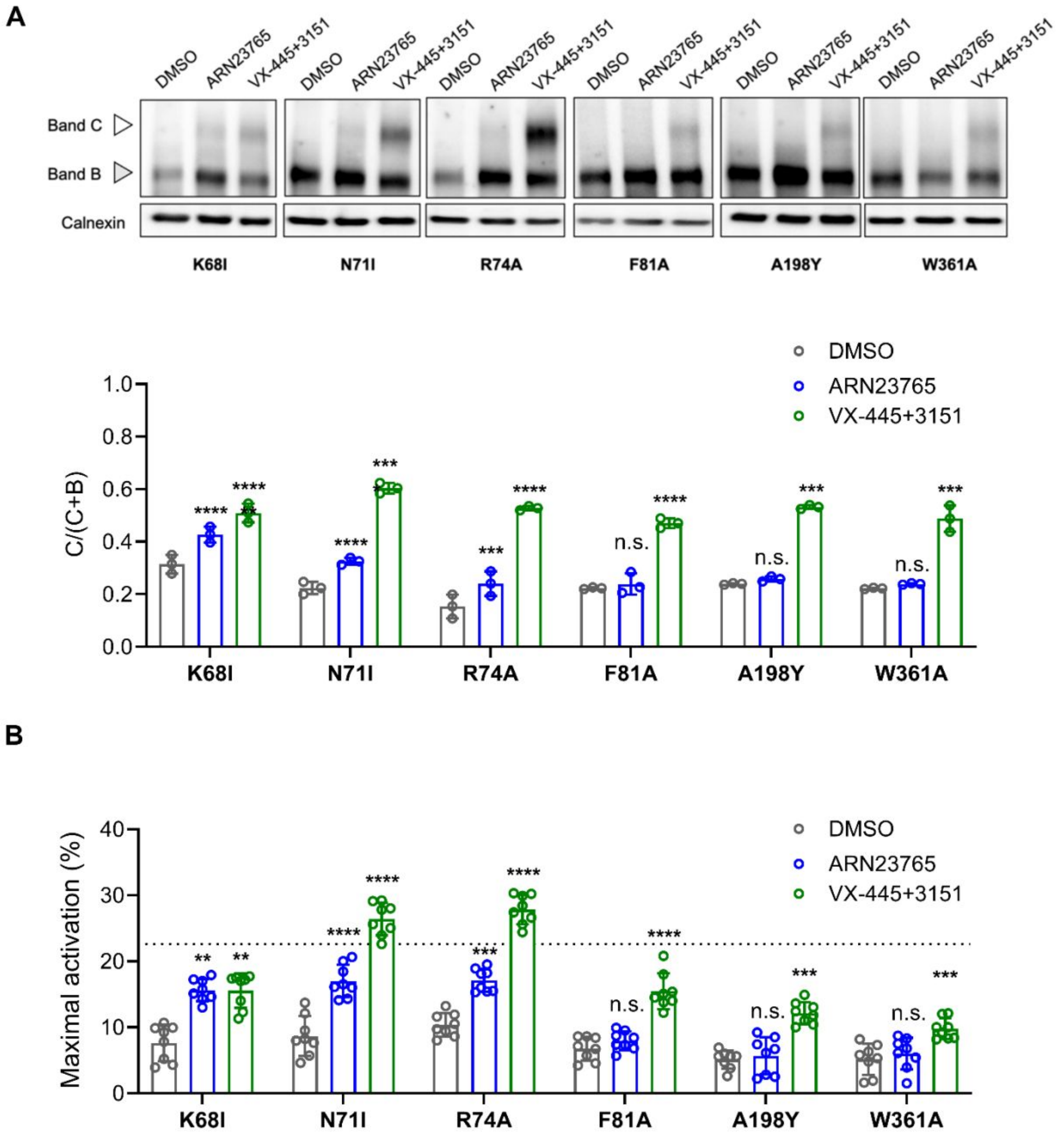

Figure S8. *In-cell mutagenesis studies at 37°C.* Experiments were conducted as described in Figure 9, except that the temperature during the incubation with correctors was kept at 37 °C. **A**) *Top*: Representative WB analysis of HEK293 cells transfected with the reported F508del additional mutants and treated with the indicated correctors for 24 h at 37 °C. *Bottom*: CFTR maturation analysis quantified using the conventional equation [C/ C+B]. **B**) CFTR channel activity in HEK293 cells transfected and treated as described in (A) was evaluated by fluorometric imaging plate reader functional assay (FLIPR).[3] Bars report the peak change in fluorescence for the indicated mutants, expressed as relative to the baseline fluorescence. The level of correction of F508del-CFTR by **ARN23765** is indicated by the horizontal dropped line. For all experiments shown, mean ± SD of 3-8 replicates is reported. Statistical significance was calculated using two-way ANOVA and Dunnett's multiple comparison test (DMSO vs. corrector: non-significant (ns) $p > 0.05$; * $p < 0.05$; ** $p < 0.01$; *** $p < 0.001$; **** $p < 0.0001$).

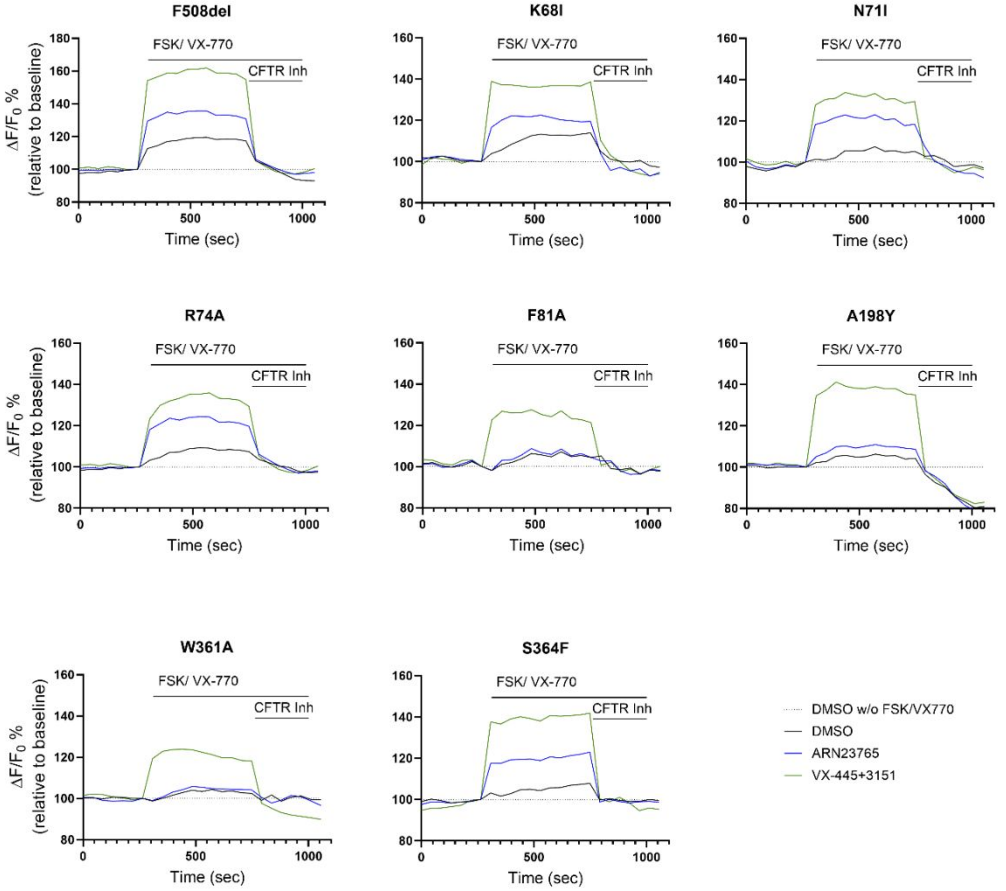

Figure S9. *Representative traces of CFTR channel activity evaluated by FLIPR assay.* The shown traces are relative to data analyses reported in Figure 9B. HEK293 cells were transfected with F508del-CFTR carrying the additional indicated mutations and treated for 24 h with **ARN23765** (10 nM) or with a control mix of corrector type II, 3151 (10 µM), and corrector type III, VX-445 (3 µM). FSK: forskolin; CFTR Inh: CFTR inhibitor-172.

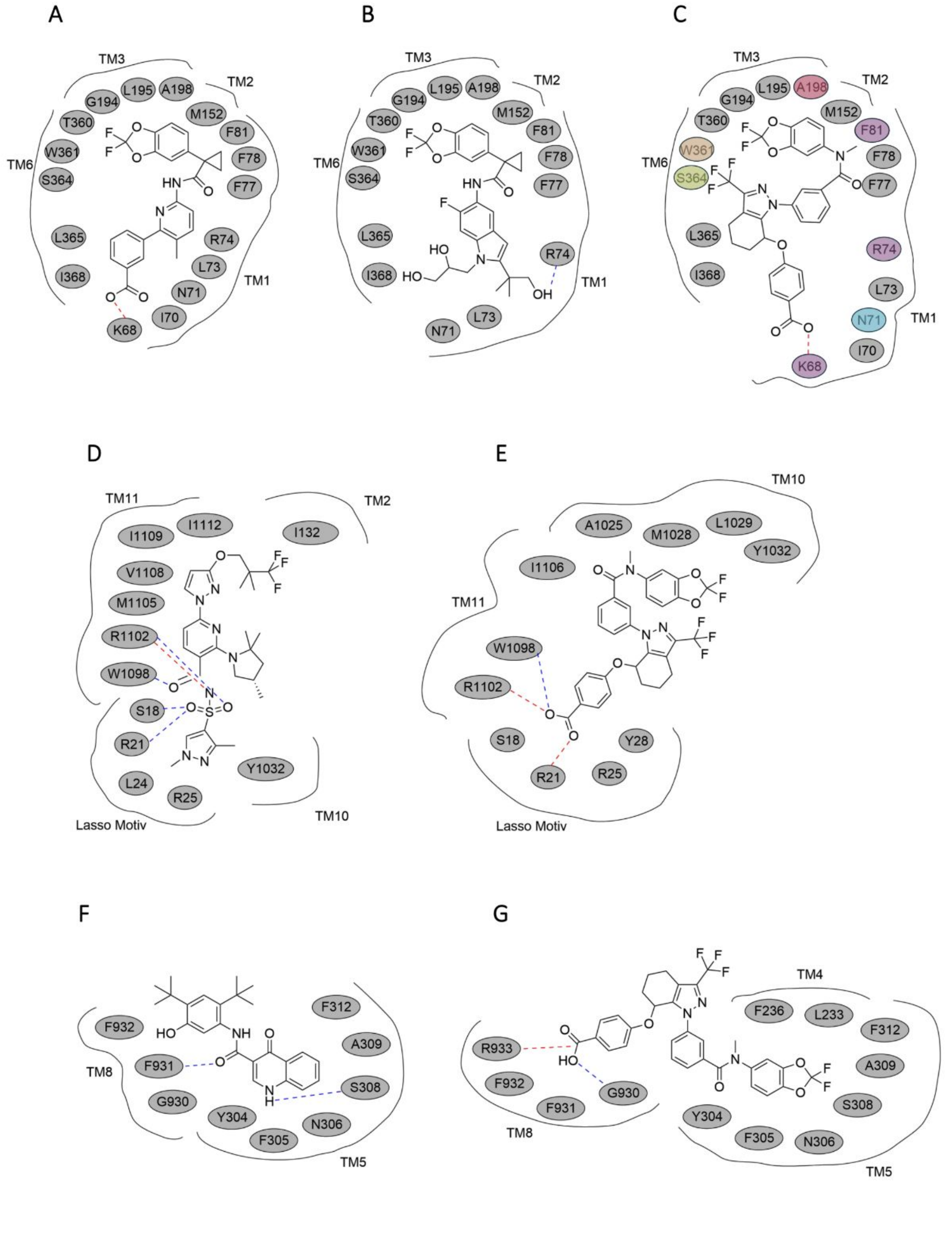

Figure S10. *2D-Binding mode comparison between **ARN23765** and former ligand after docking calculations.* A-C) 2D-Binding mode on corrector type I binding site: A) VX-809, B) VX-661, C) **ARN23765**; D-E) 2D-Binding mode on corrector type III binding site: D) VX-445, E) **ARN23765**; F-G) 2D-Binding mode on potentiator site: F) VX-770, G) **ARN23765**. Red dashed line indicates salt bridge between the ligand and the backbone residues; blue dashed lines indicate H-bonds. Colored residues in C) are related to the in silico single point mutations.

SUPPLEMENTARY BIOLOGY METHODS

HS-YFP-based assay for CFTR activity

Twenty-four hours after seeding on 96-well plates, CFBE41o$^-$ cells stably over-expressing F508del-CFTR and the HS-YFP were treated with test compounds at different concentrations in the 3 nM-10 μM range.[1] Cells treated with vehicle alone (DMSO) and with **ARN23765**, respectively, served as negative and positive controls. The following day, cells were washed with PBS containing (in mM) 137 NaCl, 2.7 KCl, 8.1 Na_2HPO_4, 1.5 KH_2PO_4, 1.0 $CaCl_2$, and 0.5 $MgCl_2$. Cells were then incubated for 25 min with 60 mL of PBS plus forskolin (20 μM) and VX-770 (1 μM) to maximally stimulate F508del-CFTR. Cells were then transferred to a microplate reader (FluoStar Galaxy; BMG Labtech, Offenburg, Germany) for CFTR activity determination. The plate reader was equipped with high-quality excitation (HQ500/20X: 500 ± 10 nm) and emission (HQ535/30M: 535 ± 15 nm) filters for YFP (Chroma Technology). Each assay comprised a continuous 14 s fluorescence reading, 2 s before and 12 s after injection of 165 mL of an iodide-containing solution (PBS with Cl^- replaced by I^-; final I^- concentration 100 μM). Data were normalized to the initial background-subtracted fluorescence. To determine I^- influx rate, the final 11 s of the data for each well were fitted with an exponential function to extrapolate initial slope (dF/dt). Reproducibility of results was confirmed by performing three independent experiments.

Western blot

Cell lysates or eluates from pull-down experiments were resolved by SDS-PAGE on 4-8 % *tris*-acetate gels (EA0378BOX, Life technologies) and were transferred into PVDF membranes (Immobilion-E IEVH00005, Millipore). CFTR fragments were resolved by SDS-PAGE on 4-12 % *tris*-glycine gels (Life Technologies) and transferred into nitrocellulose membranes (Bio-Rad). Membranes were then blocked with 5 % non-fat dry milk powder dissolved in PBS/0.1 % Tween-20. Antibody-596 (American CFF) (1:5000) was used for CFTR identification in pull-down studies and analysis of full-length CFTR in protein maturation assays and for MSD2-NBD2 analysis; mAb MM13-4 (05-581 Sigma-Aldrich) (1:5000) was used for the analysis of MSD1-contaning domains; monoclonal antibody A52 (kindly provided by Dr. D. Clarke, University of Toronto) (1:1000) was used for the analysis of MSD2-contaning domains. For calnexin detection the antibody sc46669 (Santa Cruz Biotechnology) was used. Images were acquired with Chemidoc MP (BIO-RAD laboratories) and quantified with Image J software.

CFTR Channel function analysis by fluorometric imaging plate reader functional (FLIPR) assay

FLIPR assay was performed as previously described.[3] Briefly, HEK293 cells were seeded in 96-well plates (black, flat bottom, Corning) and transfected with mutated CFTR constructs. After 18 h, cells were treated

with either DMSO (0.2%), VX-661 (3 μM), **ARN23765** (10 nM) or VX-445 (3 μM) + corrector 3151 (10 μM) for 24 h at 30 °C or 37 °C. Cells were then loaded with blue membrane potential dye dissolved in chloride free buffer. The plate was then read in a fluorescence plate reader (SpectraMax i3, Molecular Devices) at 37 °C (excitation: 530 nm, emission: 560 nm). CFTR was stimulated with 10 μM forskolin (FSK, Sigma) and potentatior VX-770 (1 μM) (Selleck Chemicals). CFTR-mediated depolarization was detected as an increase in fluorescence. CFTR inhibitor (Inh-172, 10 μM) (Selleck chemicals LLC) was added to inhibit CFTR. The peak changes in fluorescence were normalized to fluorescence immediately before agonist addition.

Site-directed mutagenesis

Desired mutations were introduced using the QuikChange II XL site-directed mutagenesis kit (200522, Agilent Technologies) according to the manufacturer's instructions. F508del-CFTR pCDNA3.1 was used as a template for the synthesis of mutated constructs K68I/F508del-CFTR, N71I/F508del-CFTR, R74A/F508del-CFTR, F81A/F508del-CFTR, A198Y/F508del-CFTR, W361A/F508del-CFTR, S364F/F508del-CFTR. The introduction of the mutation and the whole CFTR sequence were checked by Sanger sequencing. The used primers are listed in *Table S3*. Mutants R1070W/F508del-CFTR and R170G-CFTR were produced as previously described.[4]

2. SUPPLEMENTARY TABLES

Table S1: Molecular docking scores of VX-809, VX-445, VX-770, ***ARN23765*** *and compound* ***2*** *in F508del-CFTR (PDB-ID: 8EIQ).*

Ligand	***Binding site cavity***		
	VX-809	***VX-445***	***VX-770***
VX-809	*-8.8*	*-5.9*	*-5.9*
VX-445	*-6.0*	*-7.2*	*-4.8*
VX-770	*-4.2*	*-3.7*	*-6.5*
ARN23765	*-9.1*	*-5.3*	*-6.0*
Cmp 2	*-6.9*	*-5.3*	*-6.6*

Table S2: Binding free energies and non-covalent contributions for VX-809, VX-445, VX-770, ***ARN23765*** *and compound* ***2*** *in F508del-CFTR.*

	ΔG_bind	***Coulomb***	***Covalent***	***H-bond***	***Lipo***	***Packing***	***Self-Cont***	***Solv***	***vdW***
VX-809 in VX-809 cavity	-87.52	-137.41	1.76	-2.05	-31.27	-4.44	/	142.49	-56.59
VX-445 in VX-445 cavity	-68.56	-123.62	2.60	-3.63	-18.88	-1.60	/	126.19	-49.61
VX-770 in VX-770 cavity	-64.53	-24.64	0.46	-1.02	-21.59	-4.75	/	18.50	-31.48
ARN23765 in VX-809 cavity	-80.64	-113.72	-1.58	-0.16	-27.75	-4.51	/	117.61	-50.53
Cmp 2 in VX-809 cavity	-63.98	-144.98	9.41	-2.56	-21.36	-6.67	/	144.93	-43.75
ARN23765 in VX-445 cavity	-64.29-	-156.06	9.4	-3.29	-26.71	-2.99	/	162.29	-46.92
ARN23765 in VX-770 cavity	-52.61	-86.35	7.49	-1.28	-25.18	-3.56	/	97.81	-63.36

Table S3: Primers used for mutagenesis

K68I	Rv 5'-GGCTTCAAAGAAAAATCCT**AtA**CTCATTAATGCCCTTCGGCG
	Fw 5'-CGCCGAAGGGCATTAATGAG**TaT**AGGATTTTTCTTTGAAGCC
N71I	Fw 5'-CCTAAACTCATT**AtT**GCCCTTCGGCGATG
	Rv 5'-CATCGCCGAAGGGC**AaT**AATGAGTTTAGG
R74A	Fw 5'-CTCATTAATGCCCTT**gcG**CGATGTTTTTCTGGAG
	Rv 5'-CTCCAGAAAAACATCG**Cgc**AAGGGCATTAATGAG
F81A	Fw 5'-CGGCGATGTTTTTCTGGAGA**gcT**ATGTTCTATGGAATC
	Rv 5'-GATTCCATAGAACAT**Agc**TCTCCAGAAAAACATCGCCG
A198Y	Fw 5'-GATGAAGGACTTGCATTG**tat**CATTTCGTGTGGATCGCTCC
	Rv 5'-GGAGCGATCCACACGAAATG**ata**CAATGCAAGTCCTTCATC
W361A	Fw 5'-CTGGGCTGTACAAACA**gcG**TATGACTCTCTTGGAGC
	Rv 5'-GCTCCAAGAGAGTCATA**Cgc**TGTTTGTACAGCCCAG
S364F	Fw 5'-GTACAAACATGGTATGAC**TtT**CTTGGAGCAATAAAC
	Rv 5'-GTTTATTGCTCCAAG**AaA**GTCATACCATGTTTGTAC

Bold: codons coding for the mutated amino acid; *lower case*: nucleotides that were changed for the codon switch.

4. REPRESENTATIVE ^{1}H, ^{1}H-^{13}C HSQC, ^{19}F NMR SPECTRA OF FINAL COMPOUNDS

NMR experiments were run at 300 K on a Bruker Avance III 400 system (400.13 MHz for ^{1}H, and 100.62 MHz for ^{1}H-^{13}C HSQC), equipped with a BBI probe and Z-gradients, and Bruker FT NMR Avance III 600 MHz spectrometer equipped with a 5 mm CryoProbeTM QCI ^{1}H/^{19}F–^{13}C/^{15}N–D quadruple resonance, a shielded z-gradient coil and the automatic sample changer SampleJet™ NMR system (600 MHz for ^{1}H, 151 MHz for ^{13}C and 565 MHz for ^{19}F). Chemical shifts for ^{1}H and ^{13}C spectra were reported in parts per million (ppm), calibrating the residual non-deuterated solvent peak for the ^{1}H and ^{13}C to 2.50 ppm and 39.52 ppm for DMSO-d_6, whereas spectra in D_2O were referred to TSP (Trimethylsilylpropanoic acid) peak set at 0.00 ppm.

Quantitative ^{1}H NMR

Purity of the final compounds was determined by UPLC/MS and quantitative ^{1}H NMR (qNMR). qNMR experiments were acquired with 64 transients, after an automatic 90°degree pulse length optimization,[5] by using 65536 digit points, 30 s of interpulses delay, and the receiver gain fixed (64), the spectral width was 22.55 ppm with the offset positioned at 6.17 ppm. An apodization esponential function equivalent to 0.3Hz was applied to FIDs before Fourier transform. Spectra were phased, and baseline corrected, automatically. For purity evaluation by NMR assay (qNMR), the signal of final compound (10 mM solution in DMSO-d_6), was compared to the peak of an equimolar external standard solution of maleic acid (TraceCERT, 99.99%, Sigma-Aldrich, Milan, Italy), after the normalization for the number of protons generating such signals, by using the PULCON method.[6]

*(S)-N-(2,2-Difluorobenzo[d][1,3]dioxol-5-yl)-3-(7-(4-ethynylphenoxy)-3-(trifluoromethyl)-4,5,6,7-tetrahydro-1H-indazol-1-yl)-N-methylbenzamide (**1**)*

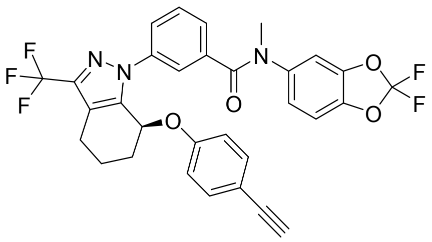

^{1}H NMR (400 MHz, DMSO-d_6)

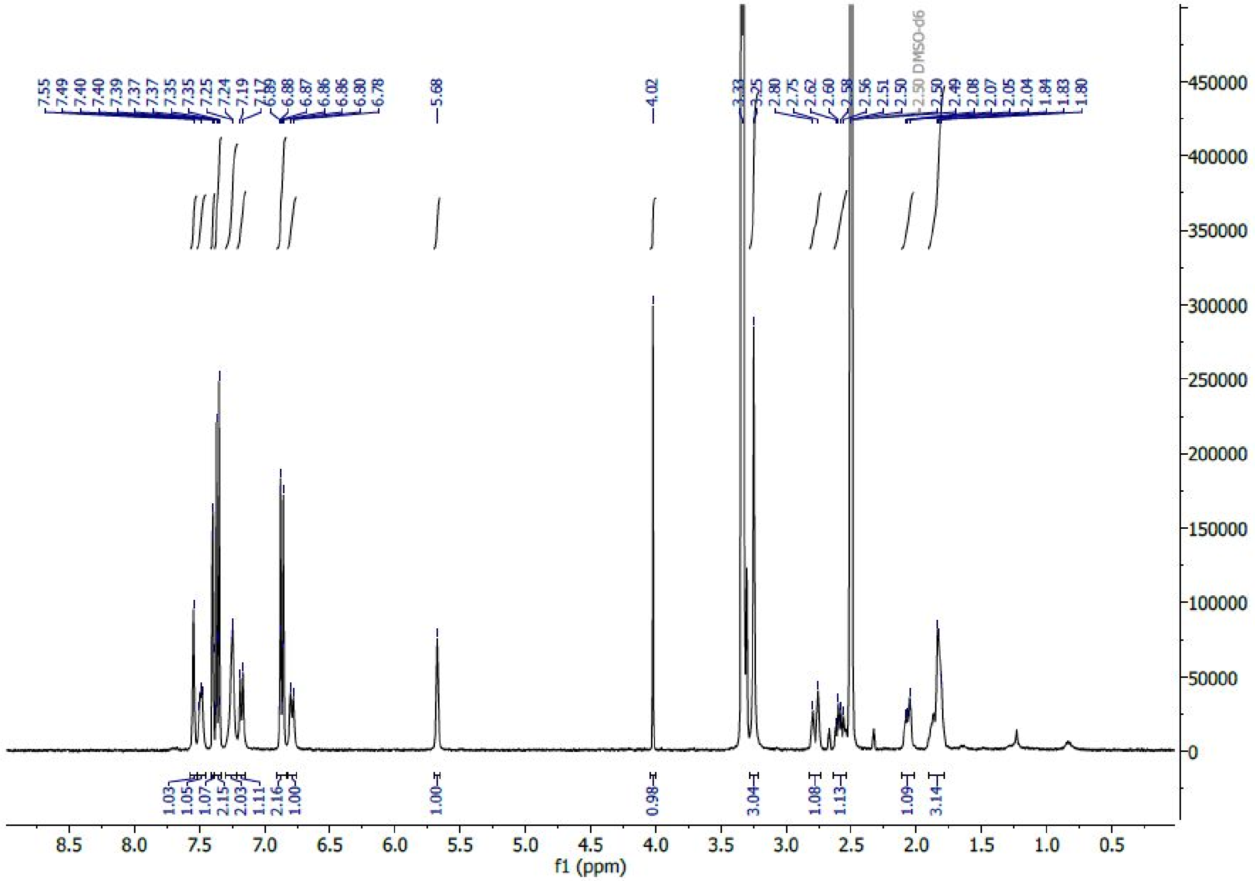

^{19}F NMR (376 MHz, DMSO-d_6)

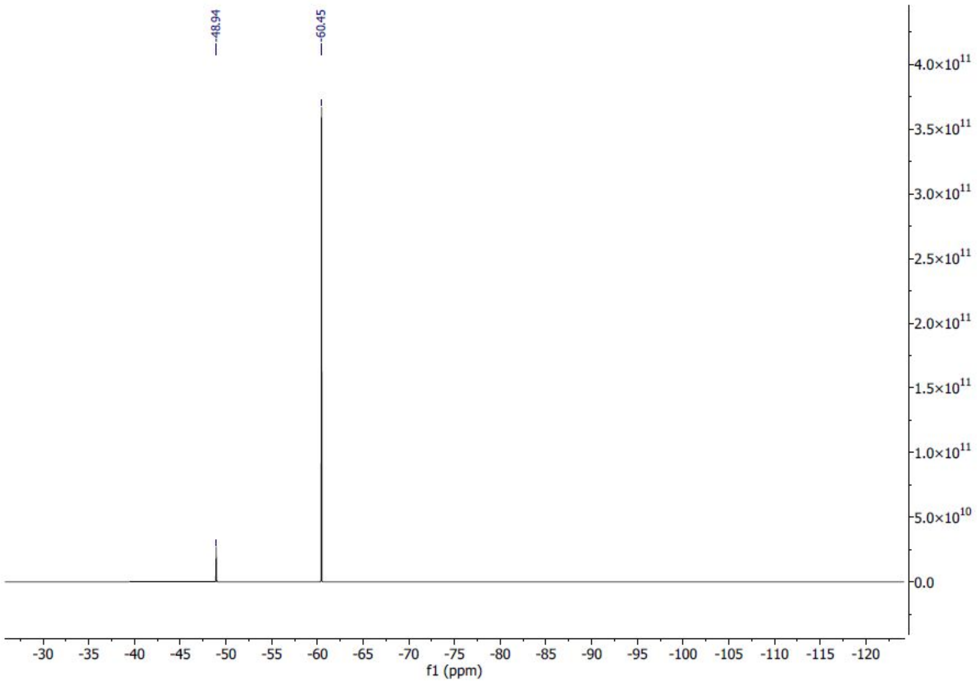

HSQC

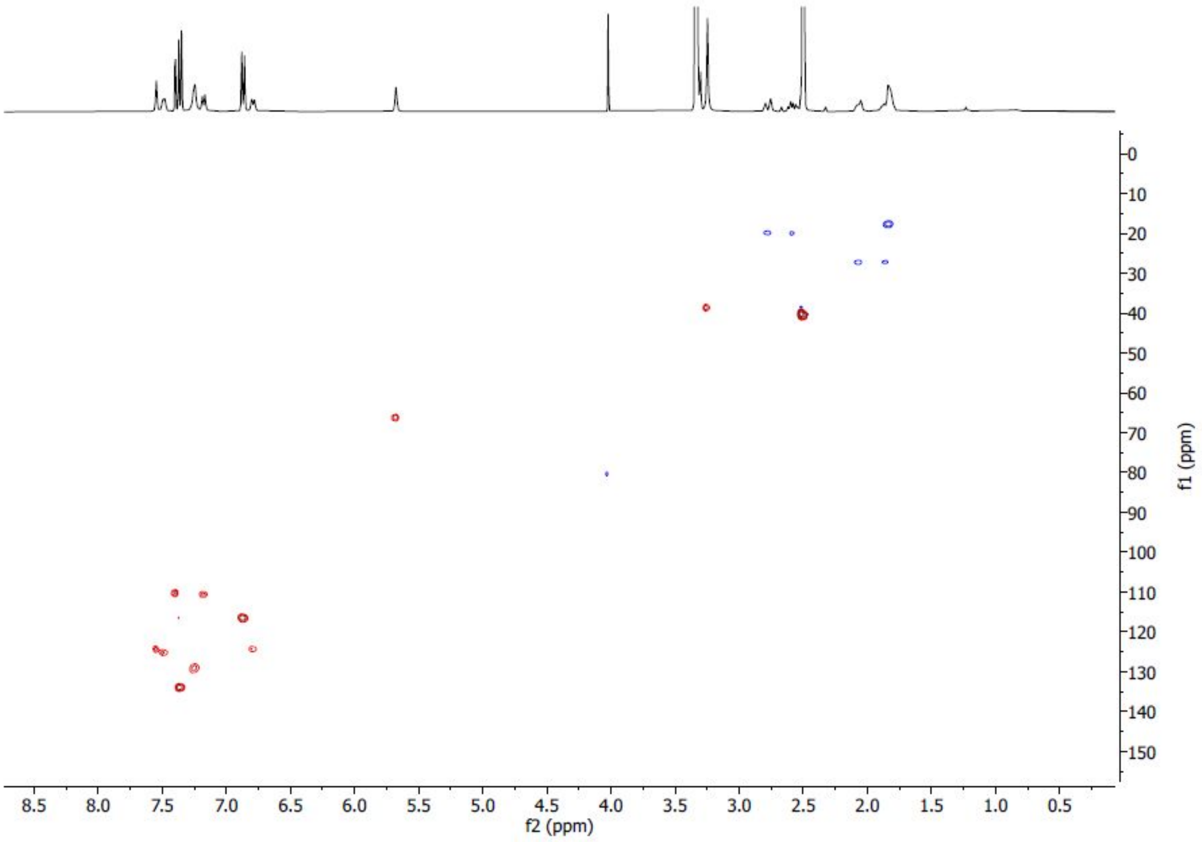

*(S)-4-((1-(3-(Methyl(4-(3-(trifluoromethyl)-3H-diazirin-3-yl)phenyl)carbamoyl)phenyl)-3-(trifluoromethyl)-4,5,6,7-tetrahydro-1H-indazol-7-yl)oxy)benzoic acid (**2**)*

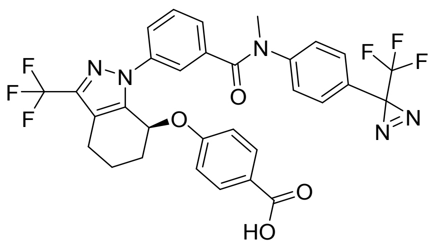

^{1}H NMR (400 MHz, DMSO-d_6)

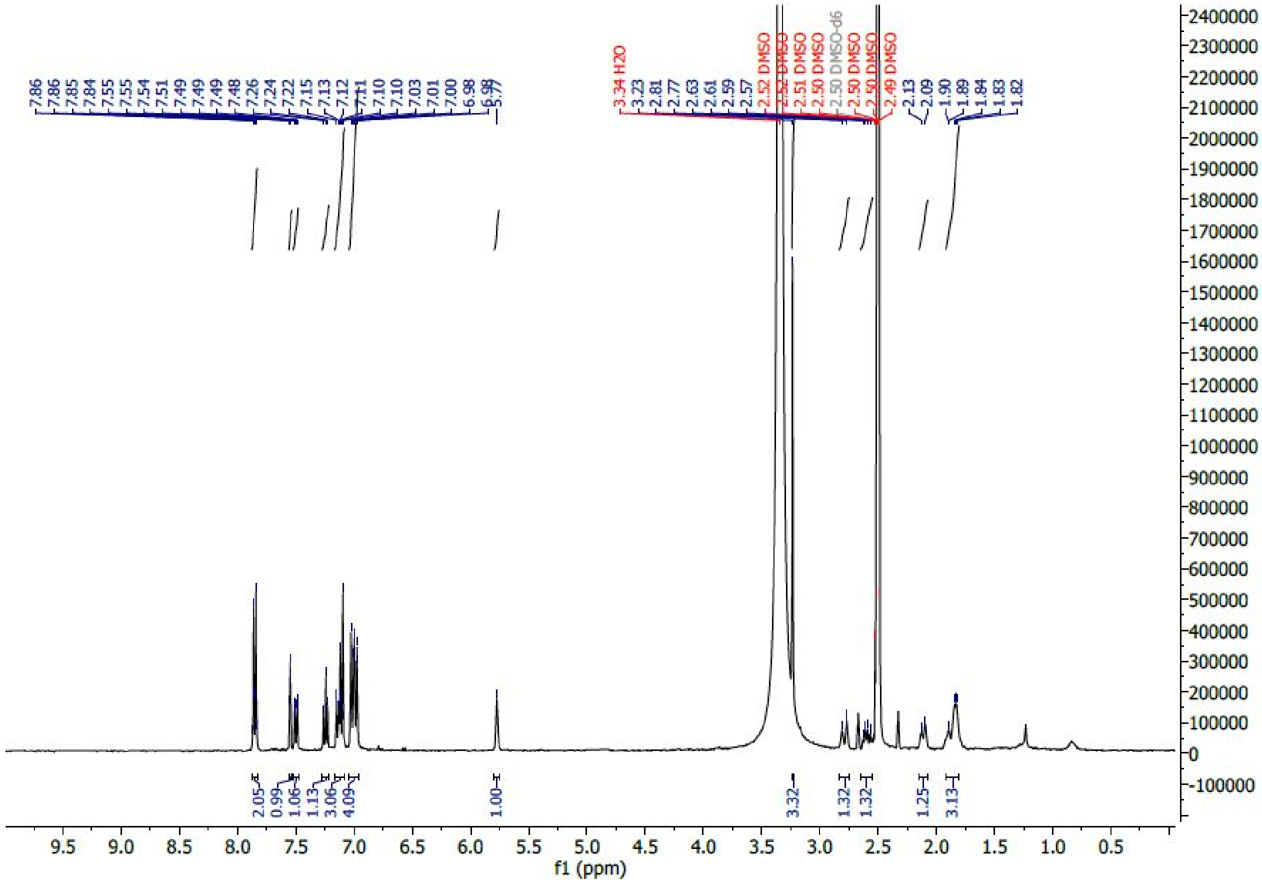

^{19}F NMR (376 MHz, DMSO-d_6)

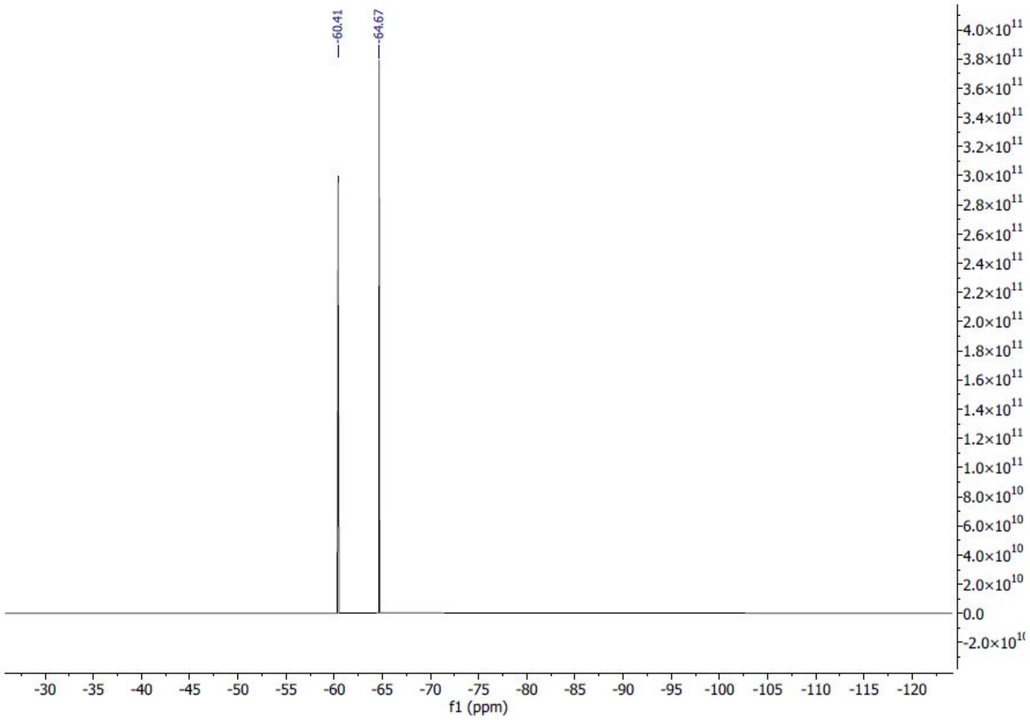

HSQC

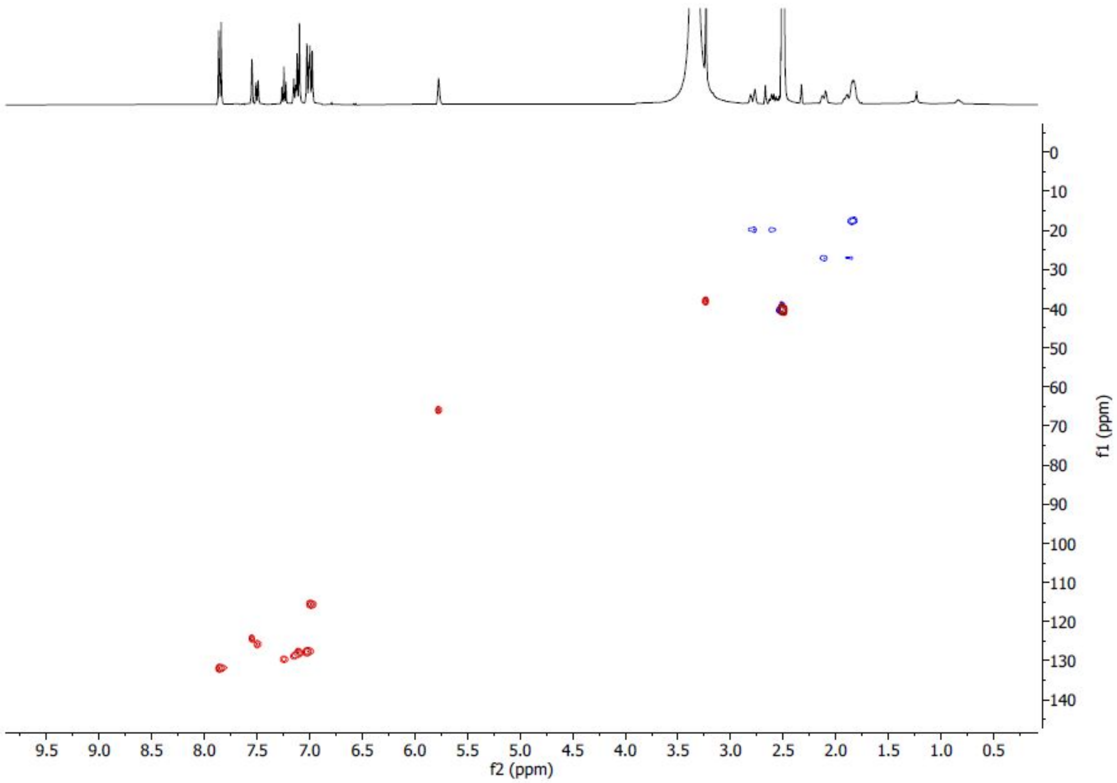

*(S)-3-(7-(4-((2-(3-(But-3-yn-1-yl)-3H-diazirin-3-yl)ethyl)carbamoyl)phenoxy)-3-(trifluoromethyl)-4,5,6,7-tetrahydro-1H-indazol-1-yl)-N-(2,2-difluorobenzo[d][1,3]dioxol-5-yl)-N-methylbenzamide (**PAP_1**)*

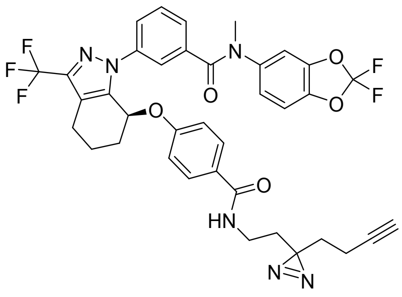

^{1}H NMR (400 MHz, DMSO-d_6)

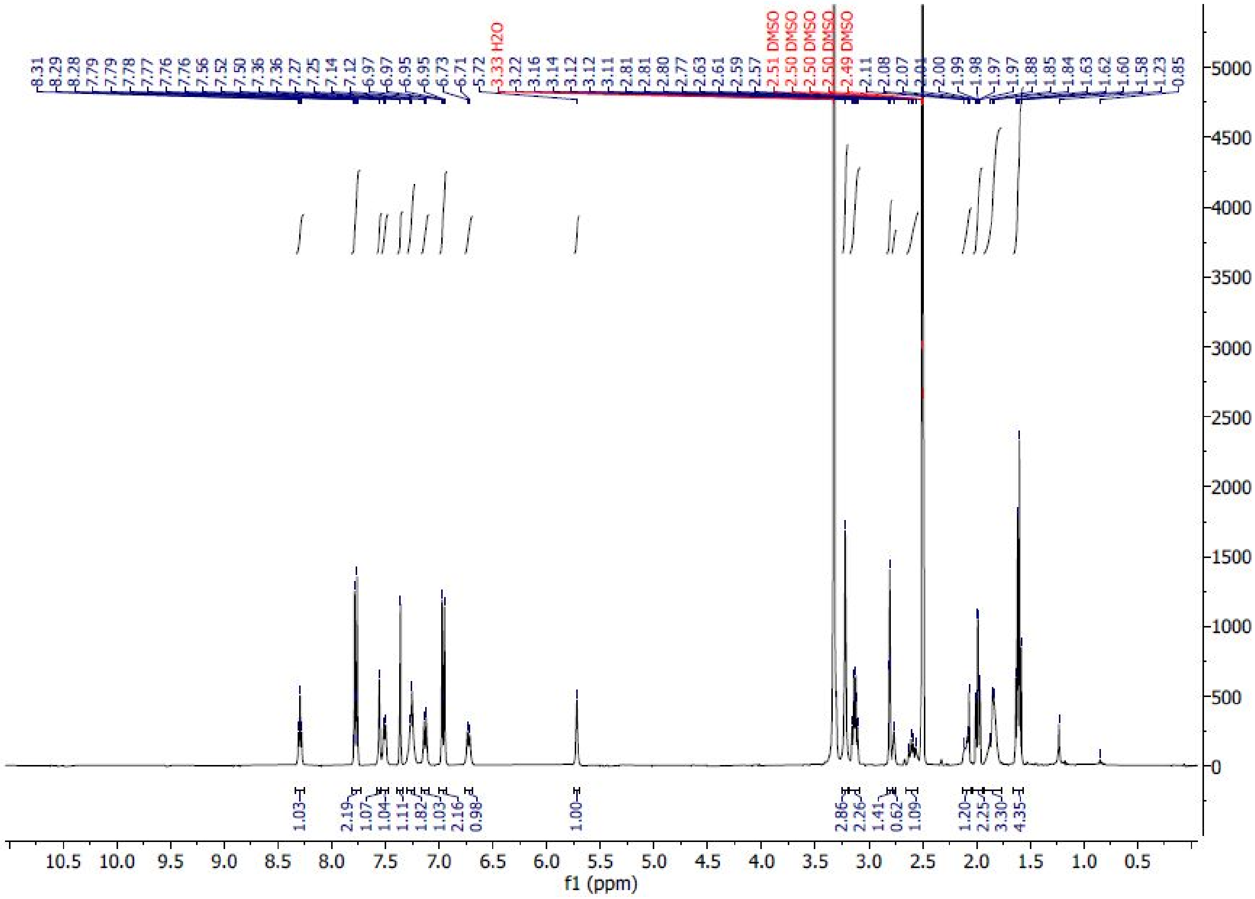

^{19}F NMR (376 MHz, DMSO-d_6)

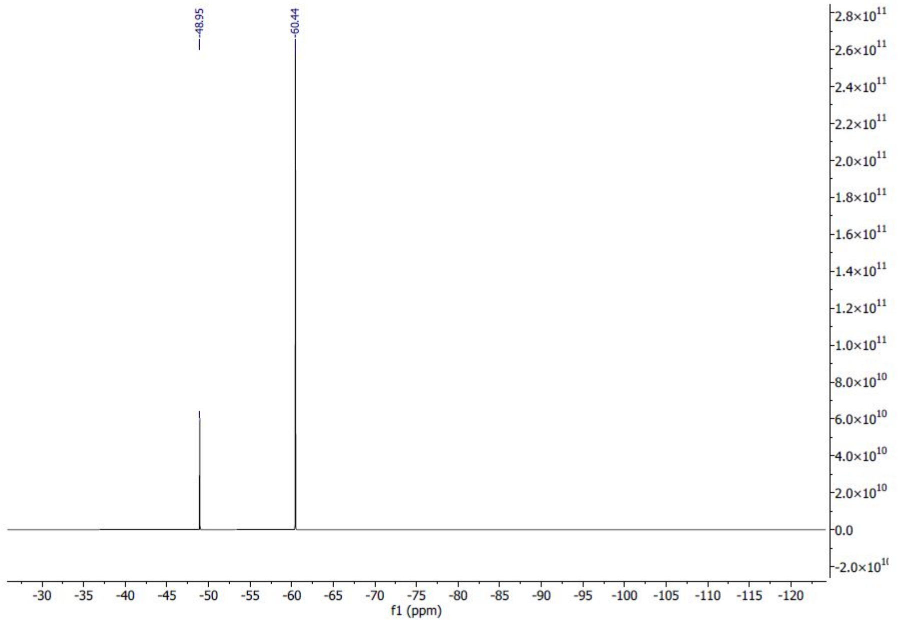

HSQC

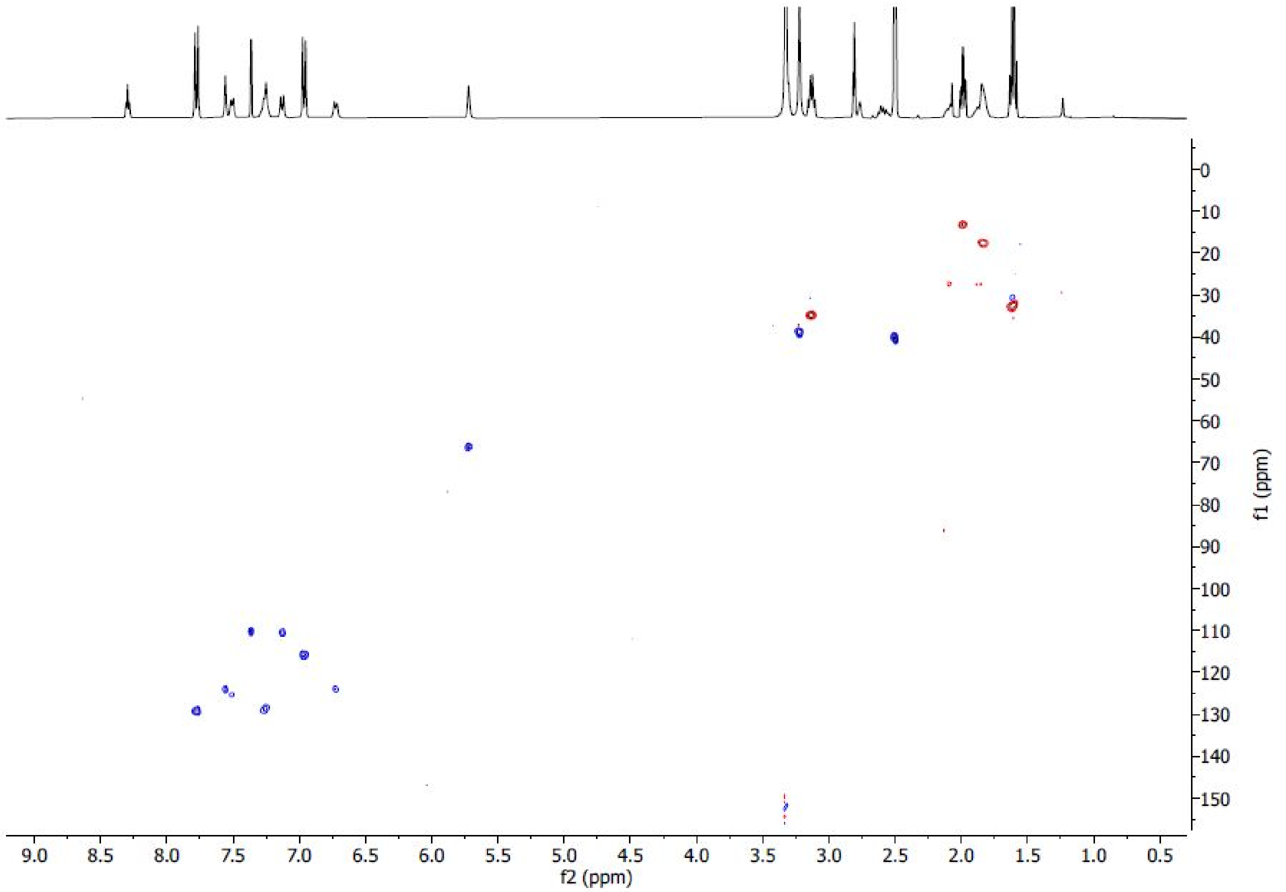

*(S)-3-(7-(4-Ethynylphenoxy)-3-(trifluoromethyl)-4,5,6,7-tetrahydro-1H-indazol-1-yl)-N-methyl-N-(4-(3-(trifluoromethyl)-3H-diazirin-3-yl)phenyl)benzamide (**PAP_2**)*

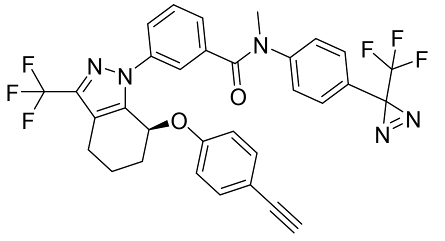

^{1}H NMR (600 MHz, DMSO-d_6)

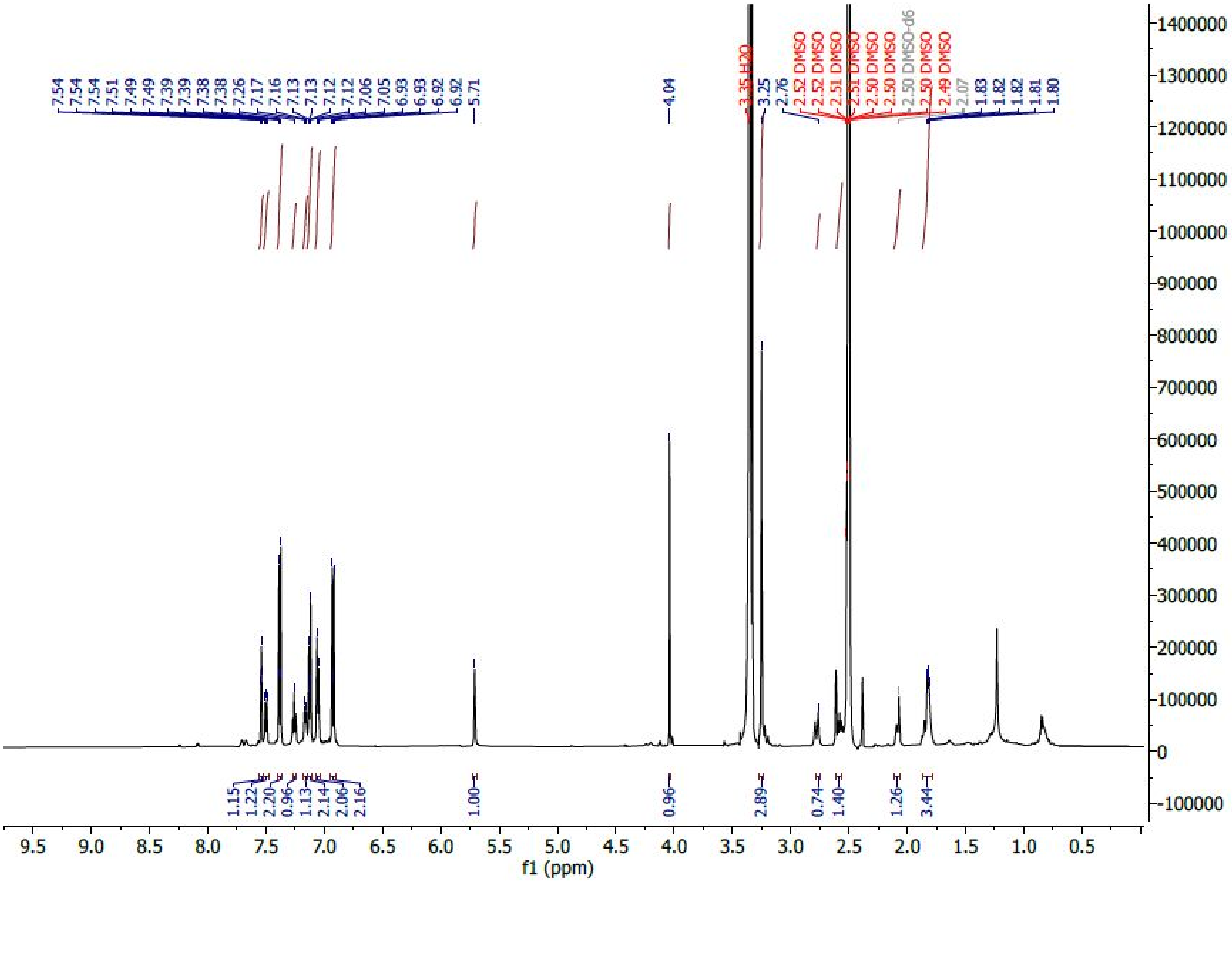

^{19}F (565 MHz, DMSO-d_6)

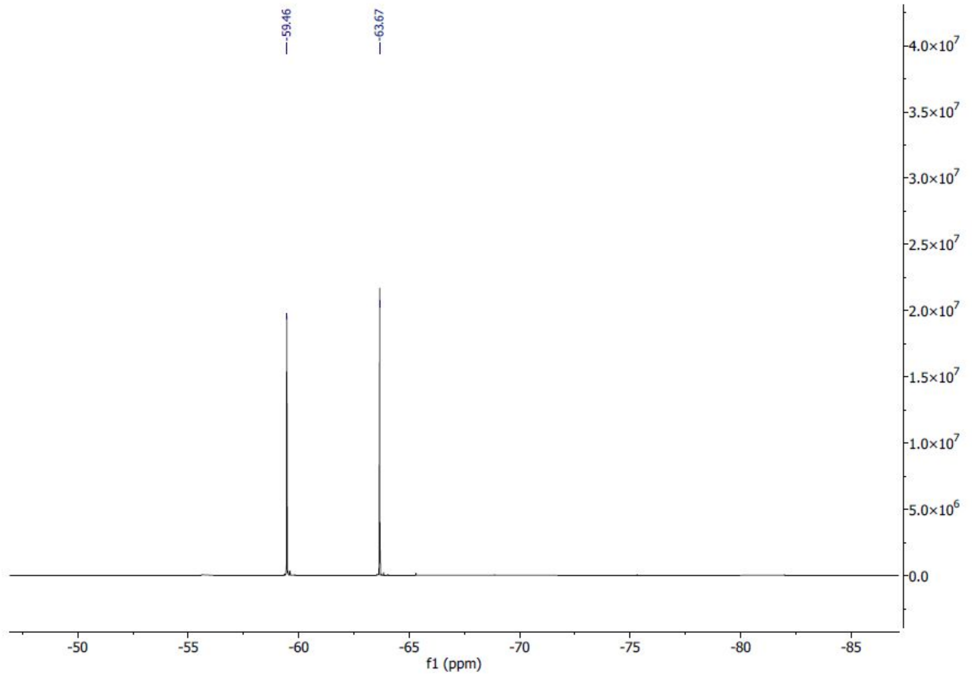

HSQC

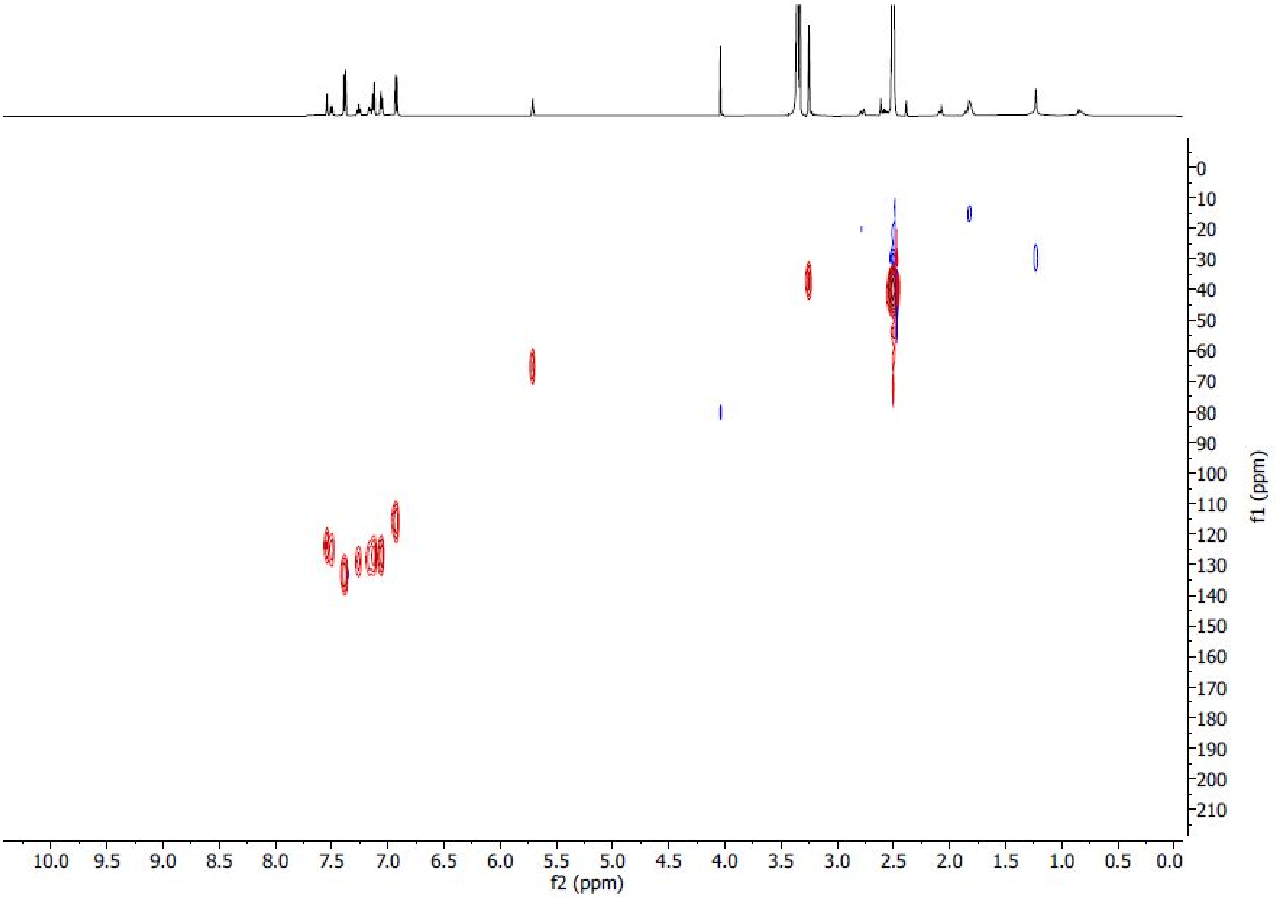

N-(2,2-Difluorobenzo[d][1,3]dioxol-5-yl)-N-methyl-3-((S)-7-(4-((2-(3-(2-(1-(18-((4S,5R)-5-methyl-2-oxoimidazolidin-4-yl)-13-oxo-3,6,9-trioxa-12-azaoctadecyl)-1H-1,2,3-triazol-4-yl)ethyl)-3H-diazirin-3-yl)ethyl)carbamoyl)phenoxy)-3-(trifluoromethyl)-4,5,6,7-tetrahydro-1H-indazol-1-yl)benzamide ***(PAP_1 DS-biot)***

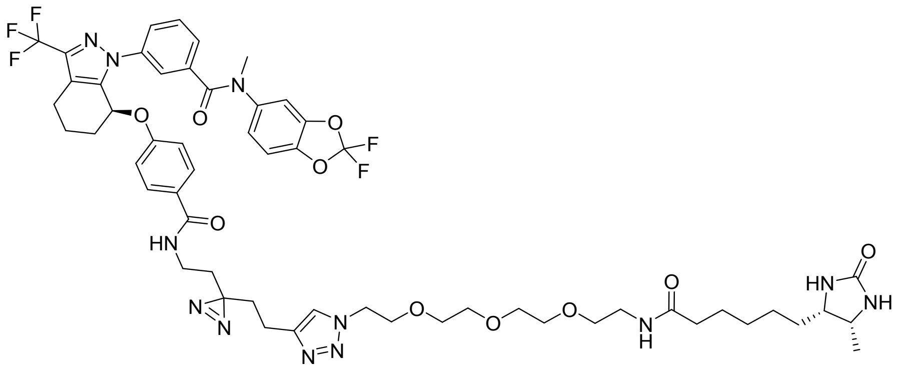

^{1}H NMR (400 MHz, DMSO-d_6)

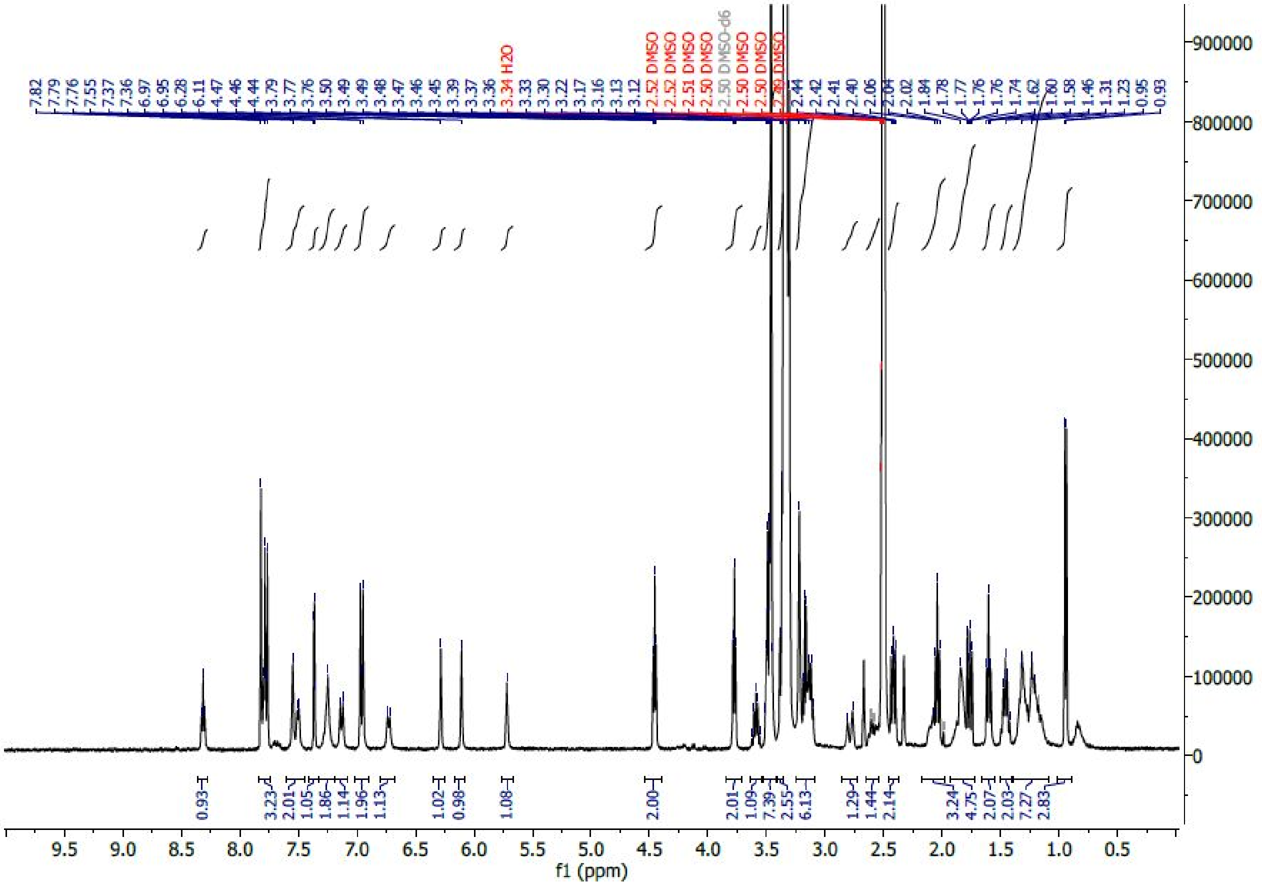

^{19}F NMR (376 MHz, DMSO-d_6)

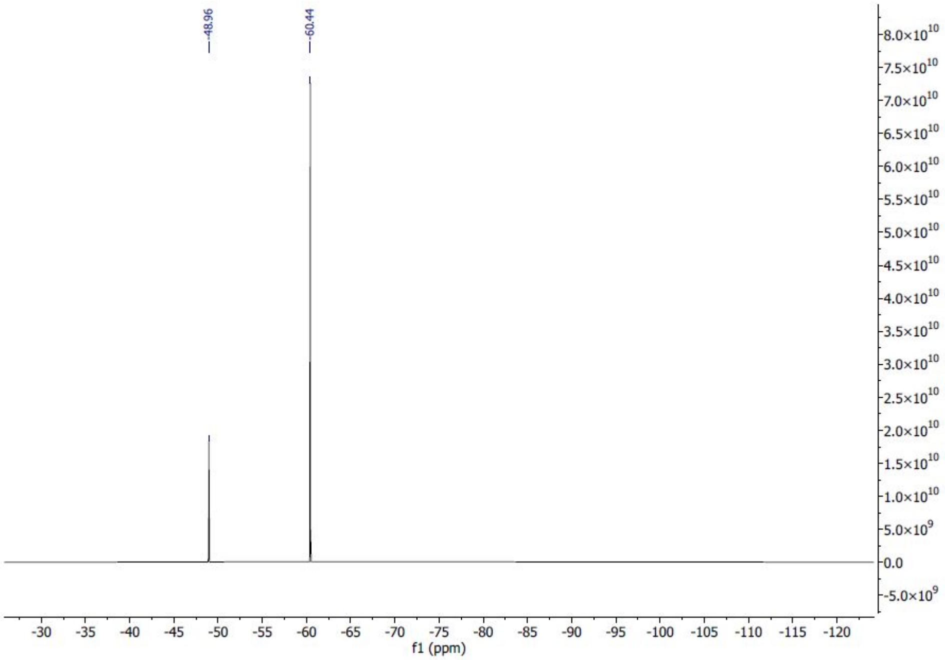

HSQC

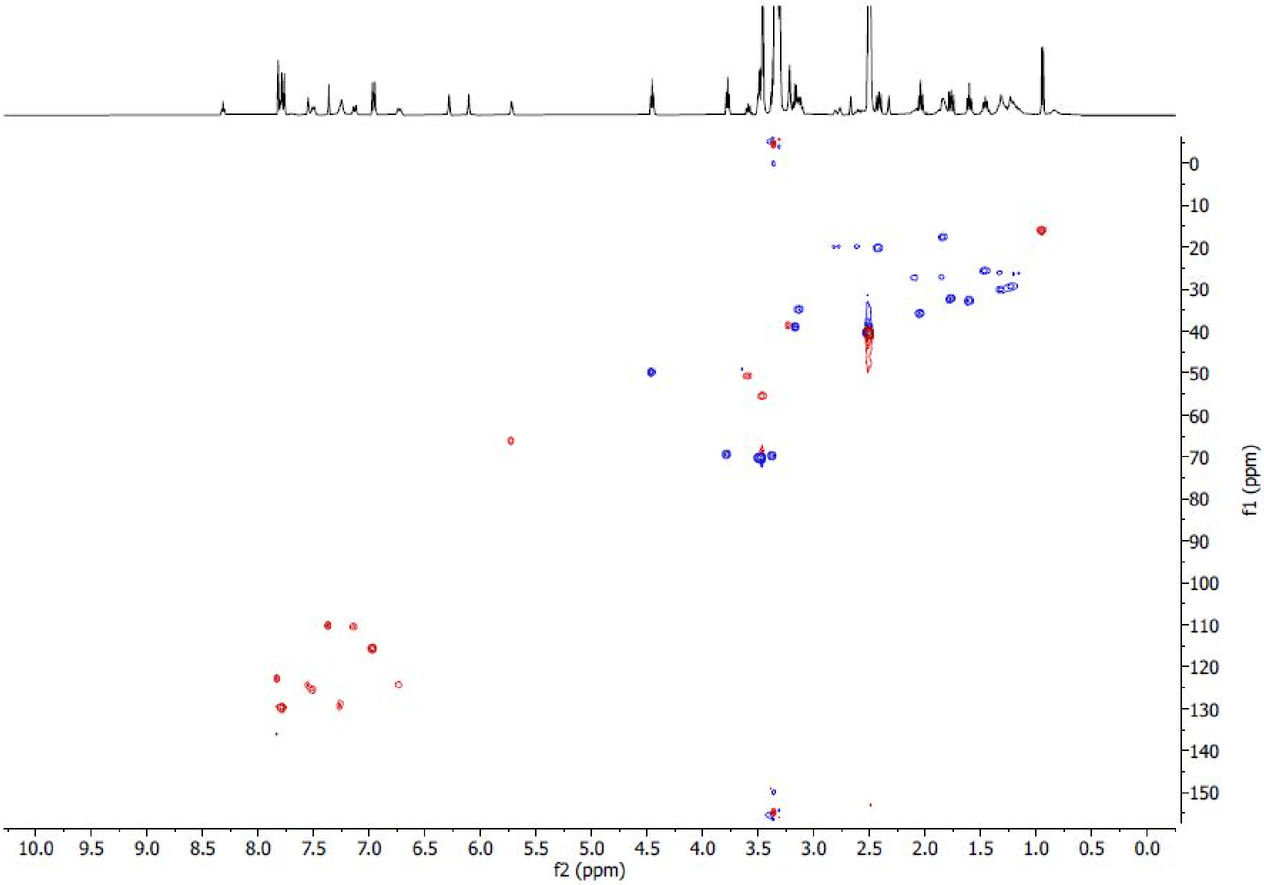

N-(2,2-Difluorobenzo[d][1,3]dioxol-5-yl)-3-((S)-7-(4-((2-(3-(2-(1-(18-((4S,5R)-5-methyl-2-oxoimidazolidin-4-yl)-13-oxo-3,6,9-trioxa-12-azaoctadecyl)-1H-1,2,3-triazol-4-yl)ethyl)-3H-diazirin-3-yl)ethyl)carbamoyl)phenoxy)-3-(trifluoromethyl)-4,5,6,7-tetrahydro-1H-indazol-1-yl)benzamide
(PAP_N1 DS-biot)

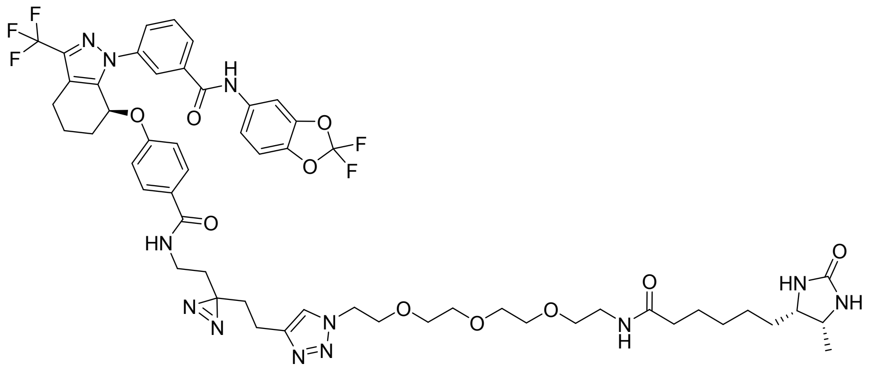

^{1}H NMR (400 MHz, DMSO-d_6)

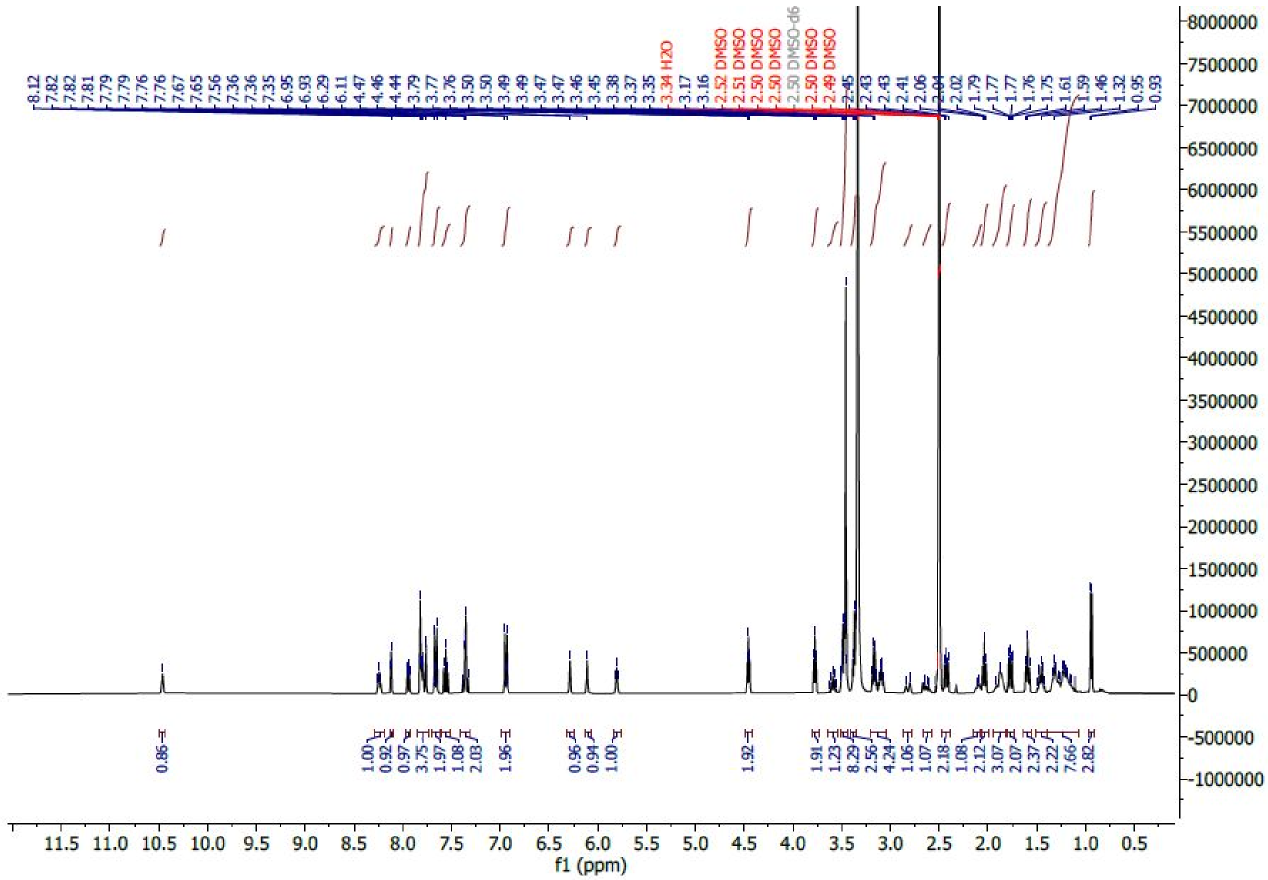

^{19}F NMR (376 MHz, DMSO-d_6)

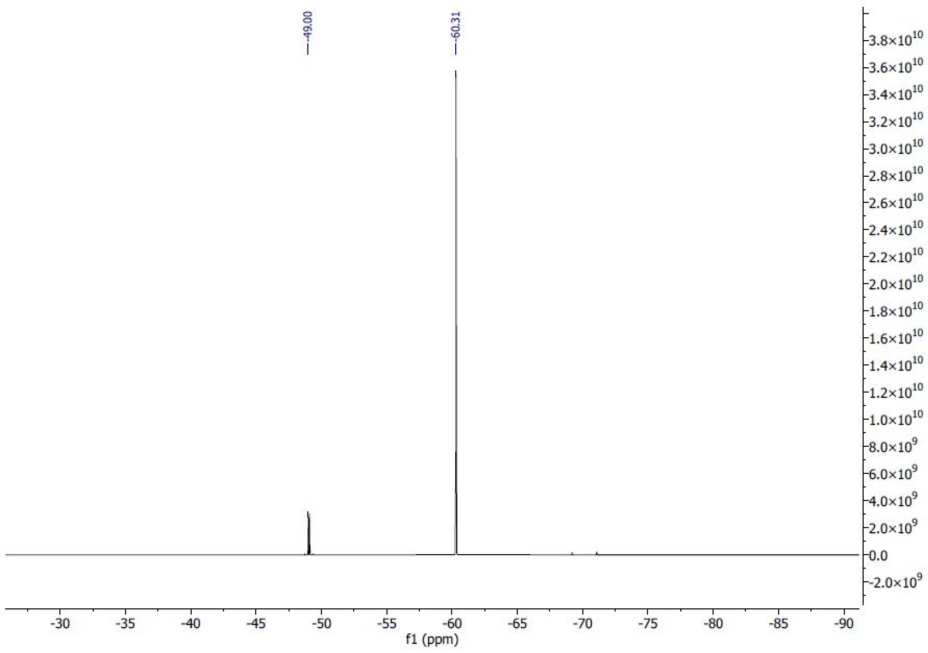

HSQC

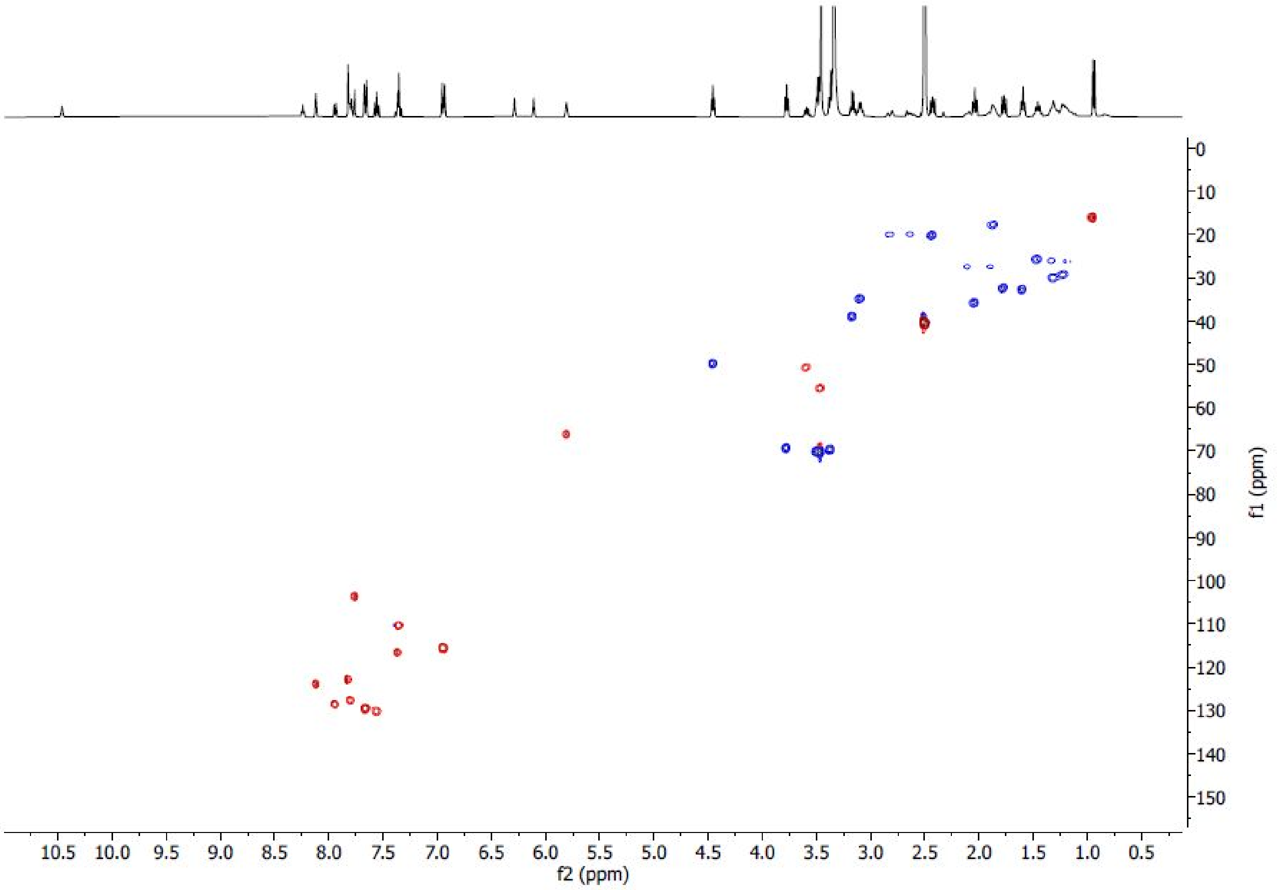

*N-Methyl-3-((S)-7-(4-(1-(18-((4S,5R)-5-methyl-2-oxoimidazolidin-4-yl)-13-oxo-3,6,9-trioxa-12-azaoctadecyl)-1H-1,2,3-triazol-4-yl)phenoxy)-3-(trifluoromethyl)-4,5,6,7-tetrahydro-1H-indazol-1-yl)-N-(4-(3-(trifluoromethyl)-3H-diazirin-3-yl)phenyl)benzamide (**PAP_2 DS-biot**)*

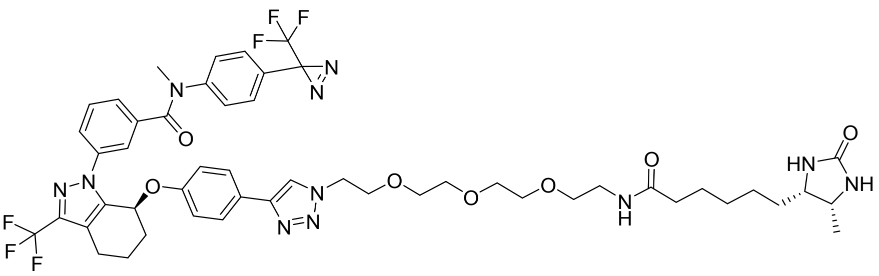

^{1}H NMR (600 MHz, DMSO-d_6)

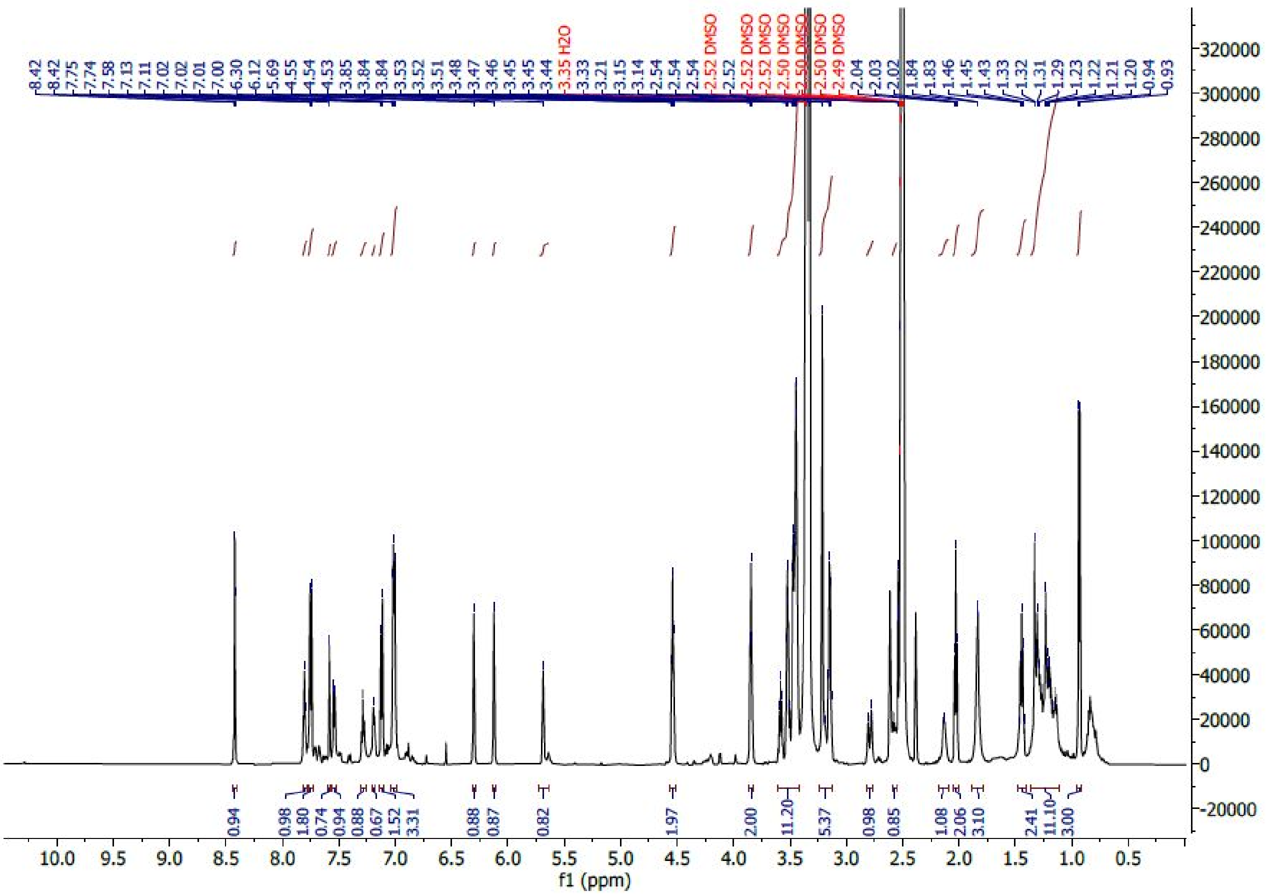

^{19}F NMR (376 MHz, DMSO-d_6)

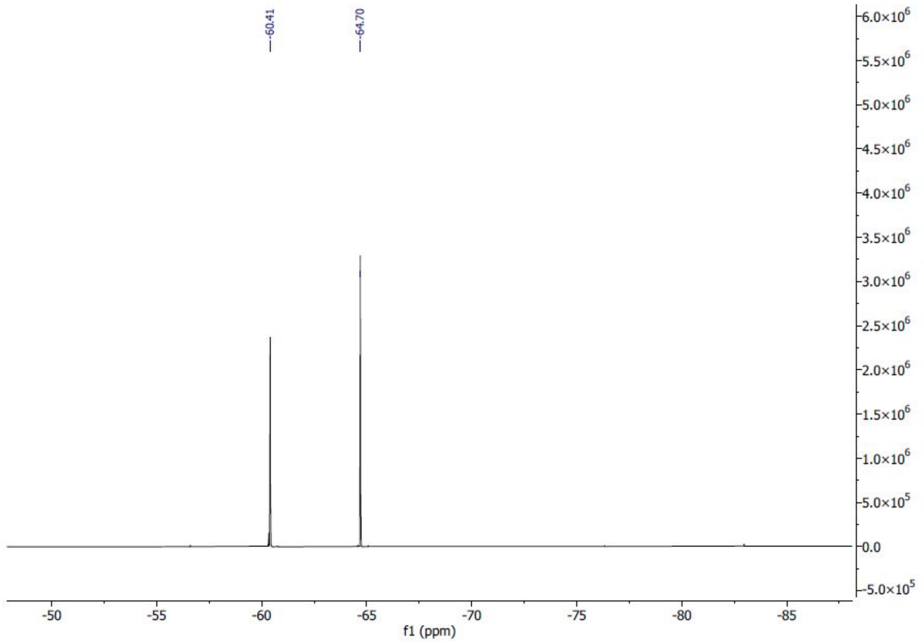

HSQC

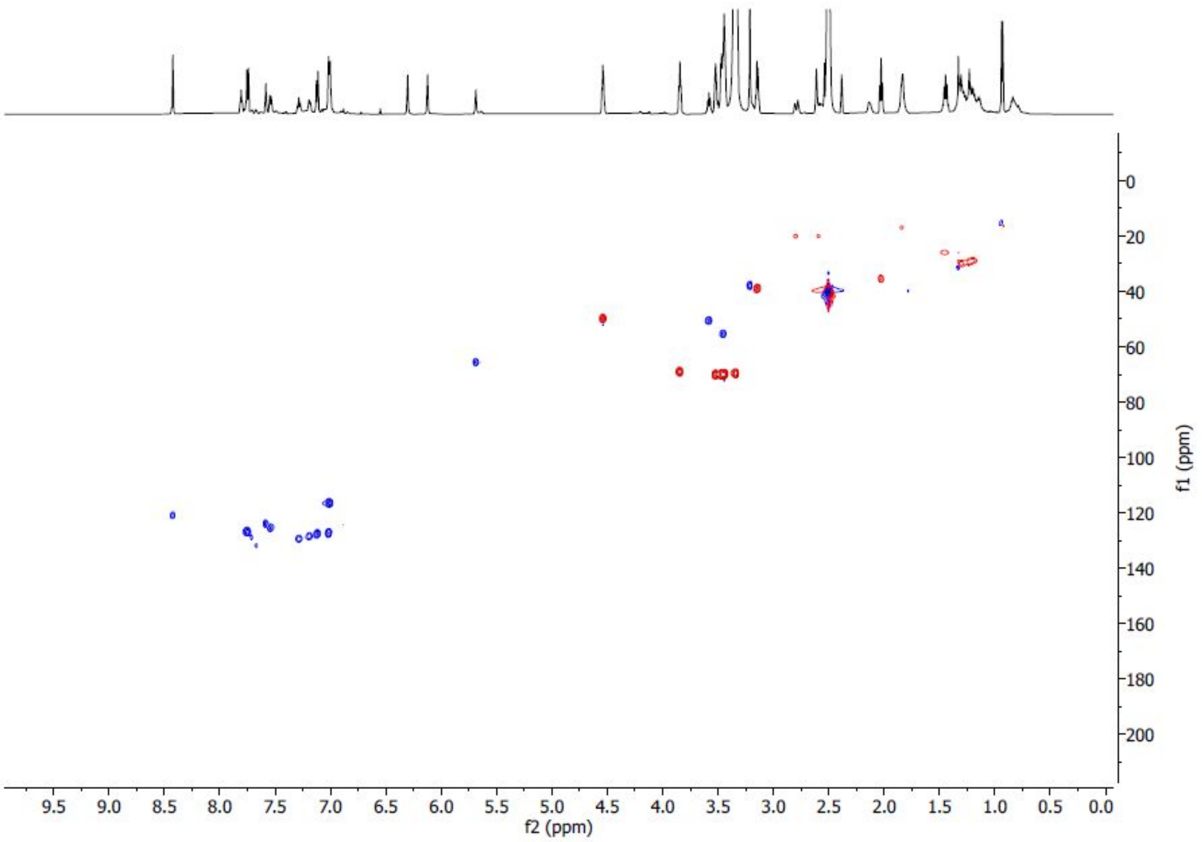

3-((S)-7-(4-(1-(18-((4S,5R)-5-Methyl-2-oxoimidazolidin-4-yl)-13-oxo-3,6,9-trioxa-12-azaoctadecyl)-1H-1,2,3-triazol-4-yl)phenoxy)-3-(trifluoromethyl)-4,5,6,7-tetrahydro-1H-indazol-1-yl)-N-(4-(3-(trifluoromethyl)-3H-diazirin-3-yl)phenyl)benzamide ***(PAP_N2 DS-biot)***

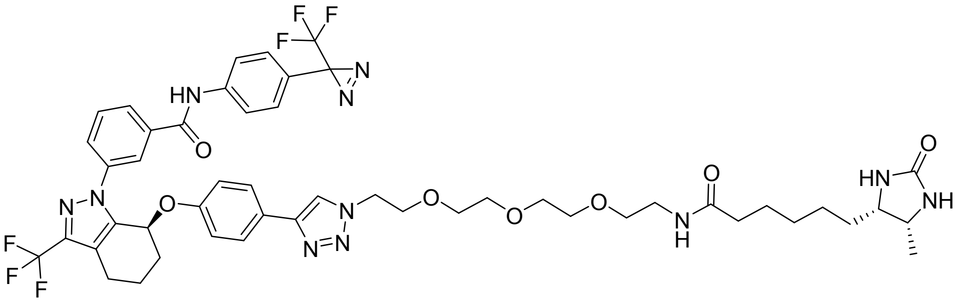

^{1}H NMR (400 MHz, DMSO-d_6)

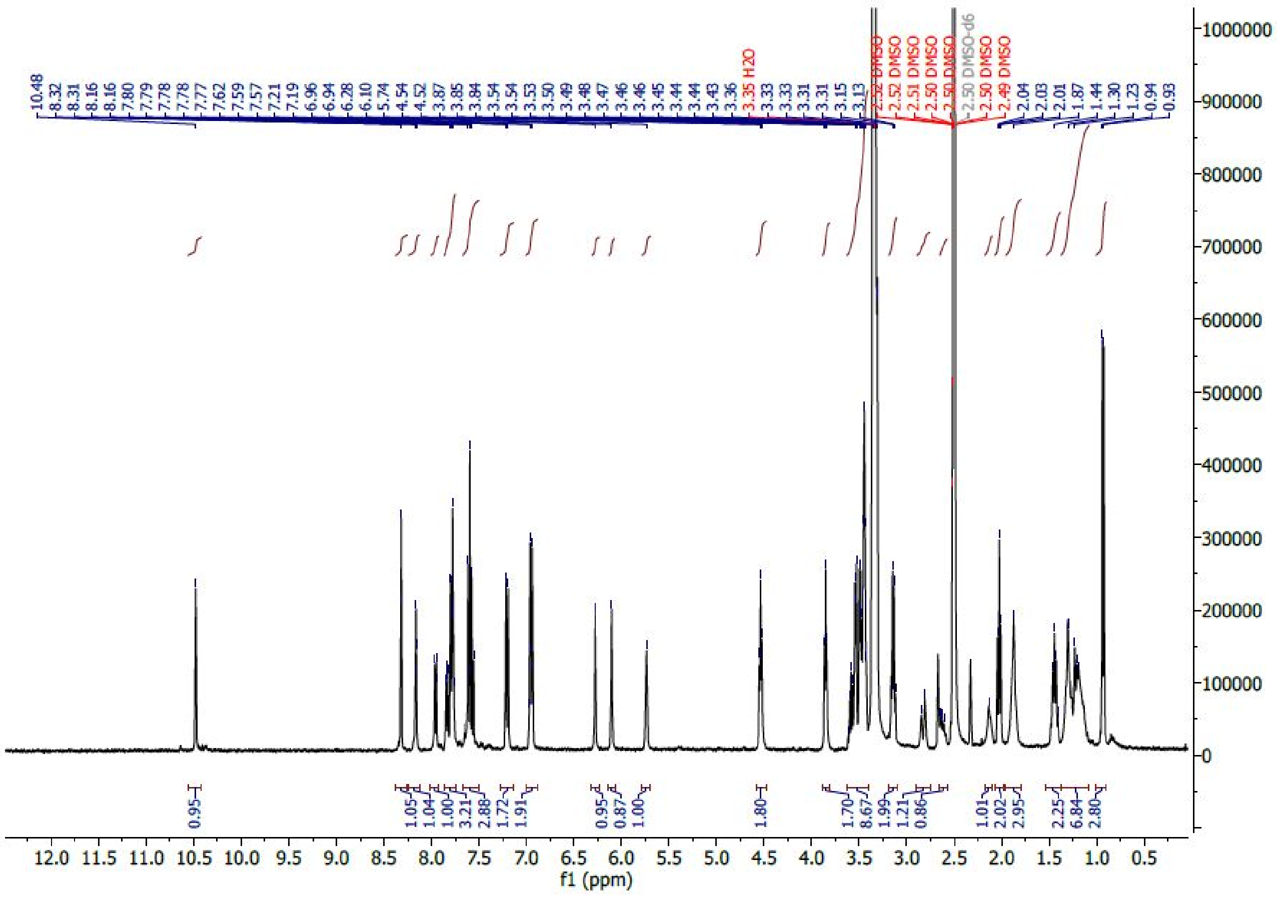

^{19}F NMR (376 MHz, DMSO-d_6)

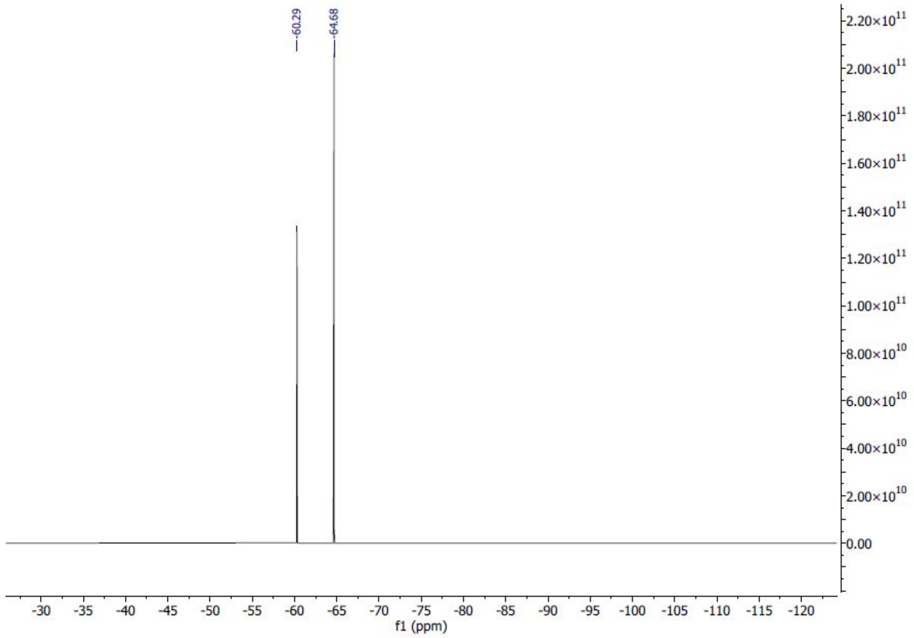

HSQC

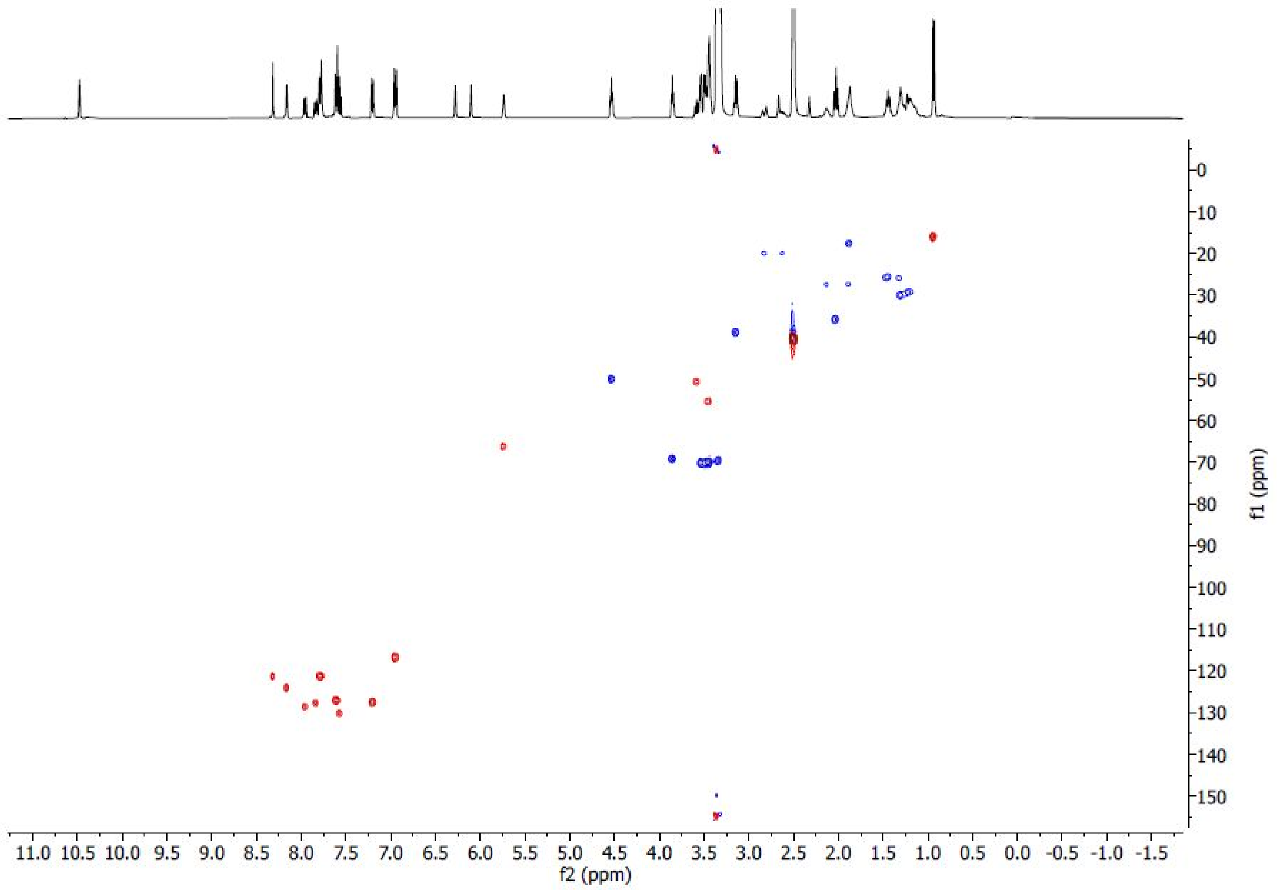

5. *LC-MS ANALYSES OF FINAL COMPOUNDS*

10 mM Stock solution of test compound was prepared in DMSO-d_6 and further diluted 20-fold with CH_3CN-H_2O (1:1) for analysis. The QC analyses were performed on a Waters ACQUITY UPLC/MS system consisting of single quadrupole detector (SQD) mass spectrometer equipped with an electrospray ionization (ESI) interface and a photodiode array detector (PDA) (from Waters Inc., Milford, MA, USA). Electrospray ionization in positive and negative mode was applied in the mass scan range 100-1200 Da. The PDA range was 210-400 nm. The analyses were run on an ACQUITY UPLC BEH C_{18} column (100 x 2.1mmID, particle size 1.7μm) with a VanGuard BEH C_{18} pre-column (5 x 2.1mmID, particle size 1.7μm). The mobile phase was 10 mM NH_4OAc in H_2O at pH 5 adjusted with AcOH (A) and 10 mM NH_4OAc in CH_3CN-H_2O (95:5) at pH 5 (B) with 0.5 mL/min as flow rate. A linear gradient was applied: 0-0.2 min: 10% B, 0.2-6.2 min: 10-90% B, 6.2-6.3 min: 90-100%, 6.3-7.0 min: 100% B.

All final compounds (**1**, **2**, **PAP_1**, **PAP_2**, **PAP_1 DS-biot**, **PAP_2 DS-biot**, **PAP_N1 DS-biot**, **PAP_N2 DS-biot**) displayed ≥95% purity as determined by UPLC-MS analysis, except for **PAP_2** (90% purity).

*(S)-N-(2,2-Difluorobenzo[d][1,3]dioxol-5-yl)-3-(7-(4-ethynylphenoxy)-3-(trifluoromethyl)-4,5,6,7-tetrahydro-1H-indazol-1-yl)-N-methylbenzamide (**1**)*

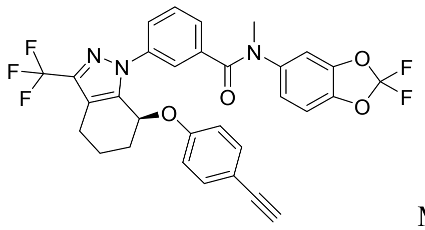

MW: 595.51

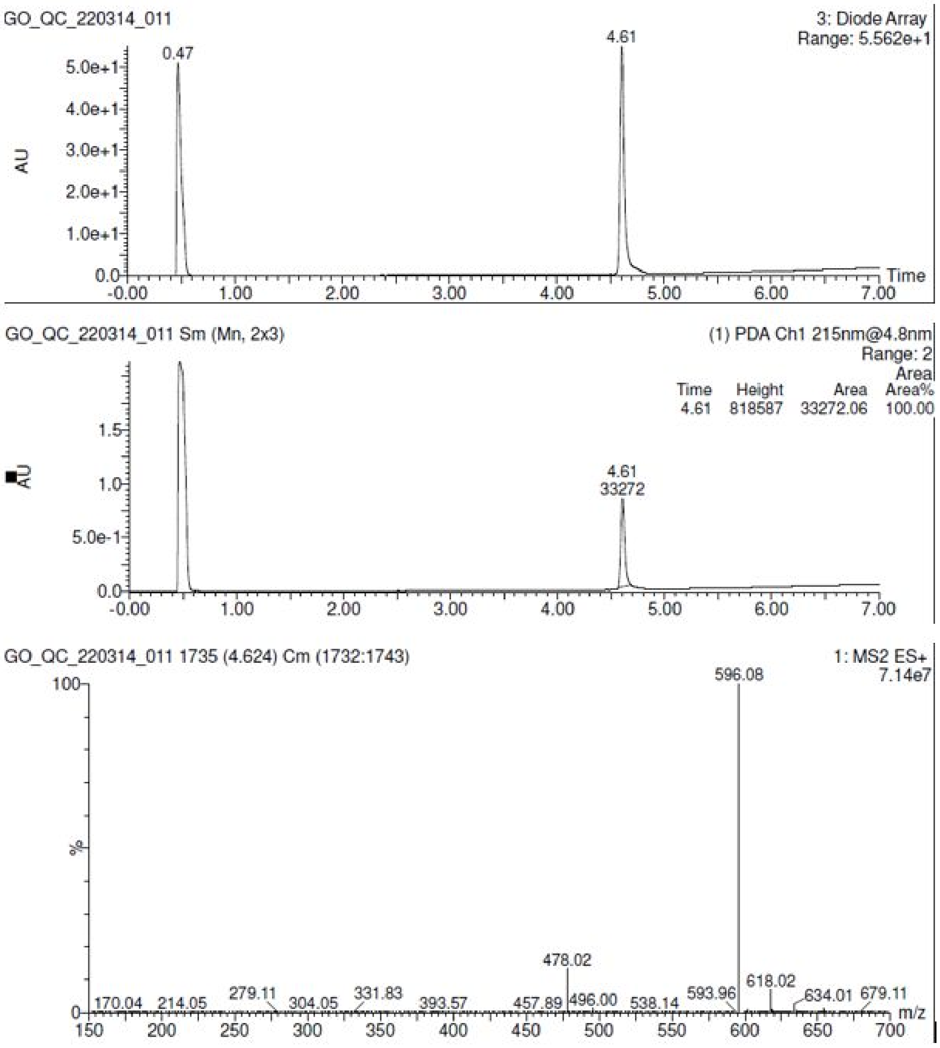

*(S)-4-((1-(3-(Methyl(4-(3-(trifluoromethyl)-3H-diazirin-3-yl)phenyl)carbamoyl)phenyl)-3-(trifluoromethyl)-4,5,6,7-tetrahydro-1H-indazol-7-yl)oxy)benzoic acid (**2**)*

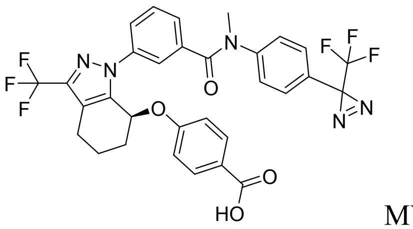

MW: 643.53

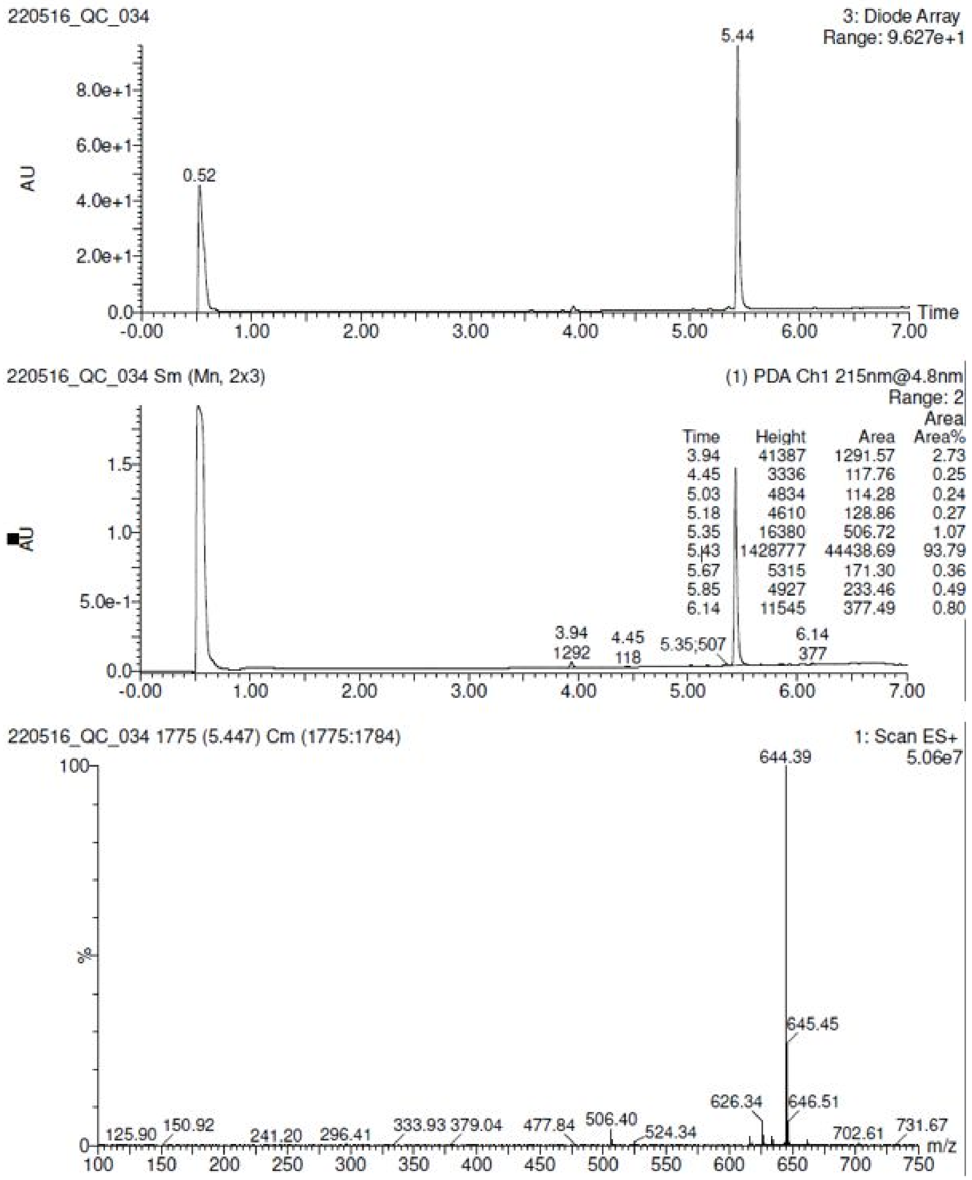

*(S)-3-(7-(4-((2-(3-(But-3-yn-1-yl)-3H-diazirin-3-yl)ethyl)carbamoyl)phenoxy)-3-(trifluoromethyl)-4,5,6,7-tetrahydro-1H-indazol-1-yl)-N-(2,2-difluorobenzo[d][1,3]dioxol-5-yl)-N-methylbenzamide (**PAP_1**)*

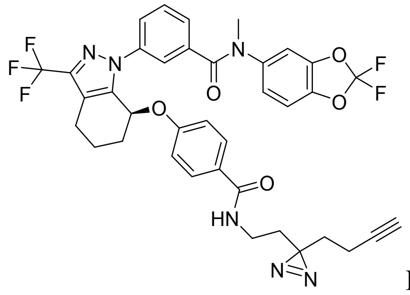

MW: 734.67

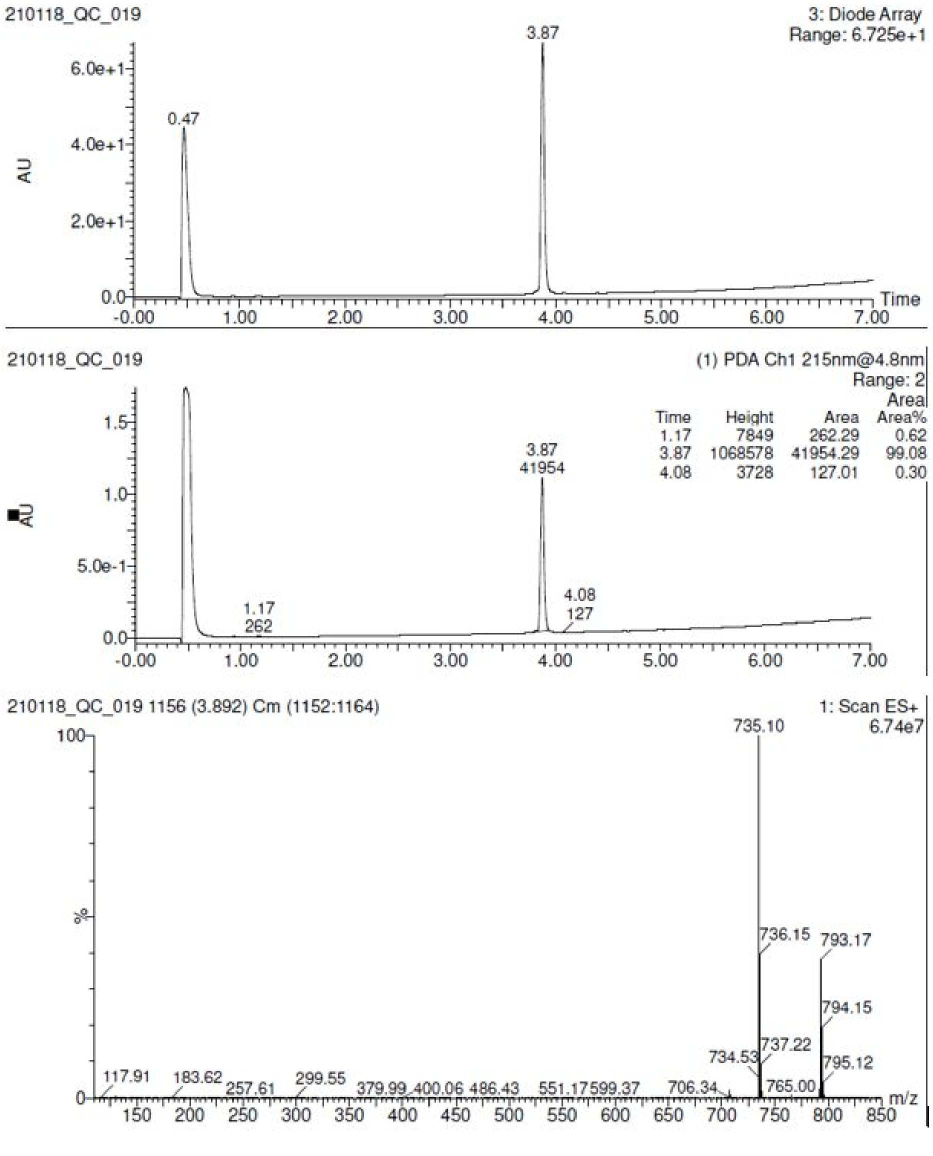

*(S)-3-(7-(4-Ethynylphenoxy)-3-(trifluoromethyl)-4,5,6,7-tetrahydro-1H-indazol-1-yl)-N-methyl-N-(4-(3-(trifluoromethyl)-3H-diazirin-3-yl)phenyl)benzamide (**PAP_2**)*

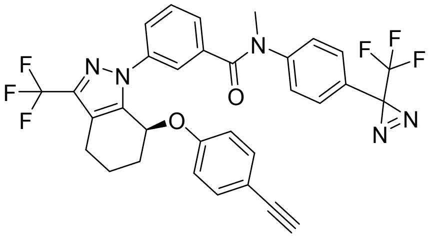

MW: 623.54

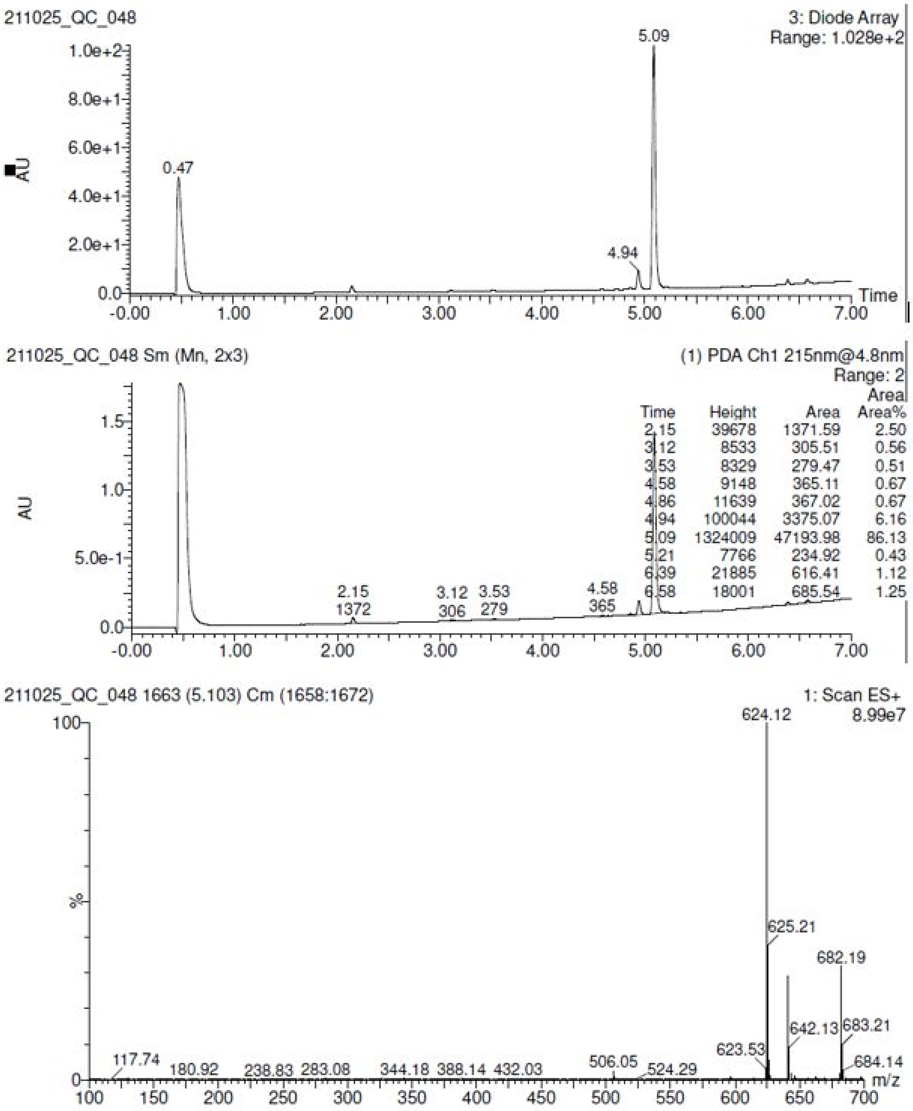

N-(2,2-Difluorobenzo[d][1,3]dioxol-5-yl)-N-methyl-3-((S)-7-(4-((2-(3-(2-(1-(18-((4S,5R)-5-methyl-2-oxoimidazolidin-4-yl)-13-oxo-3,6,9-trioxa-12-azaoctadecyl)-1H-1,2,3-triazol-4-yl)ethyl)-3H-diazirin-3-yl)ethyl)carbamoyl)phenoxy)-3-(trifluoromethyl)-4,5,6,7-tetrahydro-1H-indazol-1-yl)benzamide ***(PAP_1 DS-biot)***

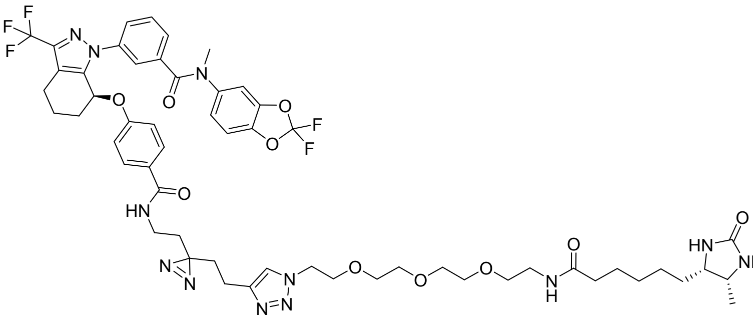

MW: 1149.17

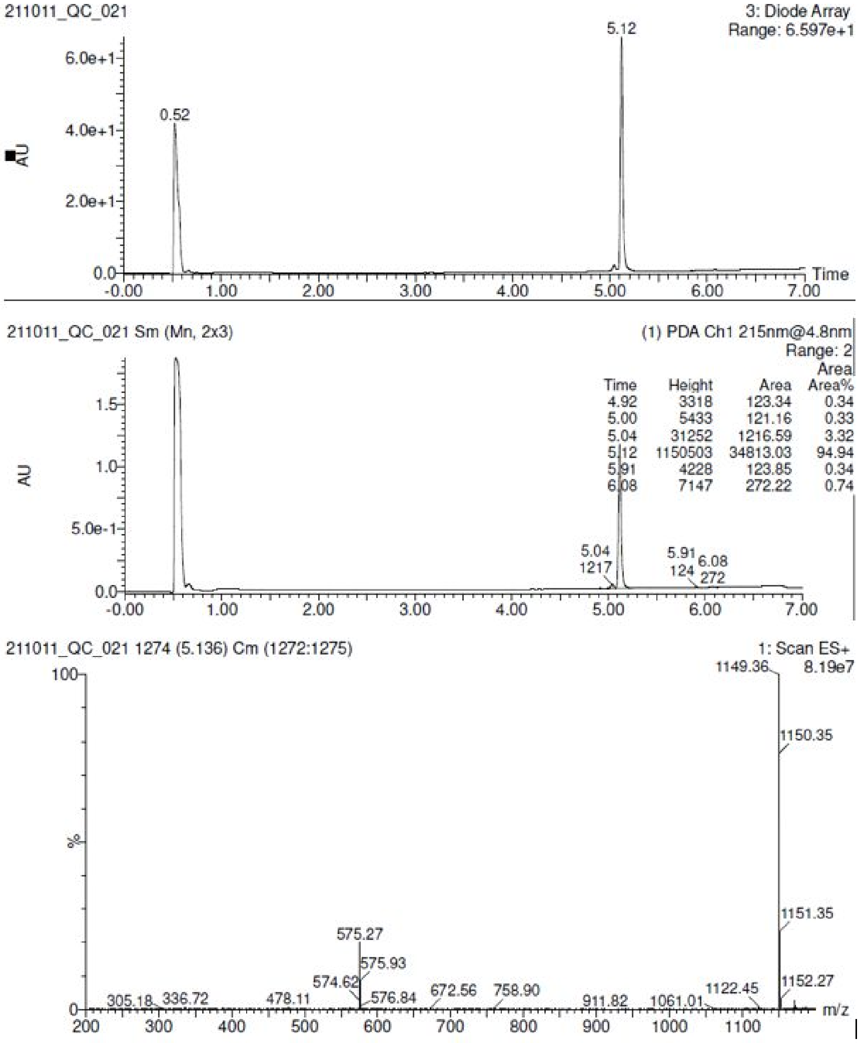

N-(2,2-Difluorobenzo[d][1,3]dioxol-5-yl)-3-((S)-7-(4-((2-(3-(2-(1-(18-((4S,5R)-5-methyl-2-oxoimidazolidin-4-yl)-13-oxo-3,6,9-trioxa-12-azaoctadecyl)-1H-1,2,3-triazol-4-yl)ethyl)-3H-diazirin-3-yl)ethyl)carbamoyl)phenoxy)-3-(trifluoromethyl)-4,5,6,7-tetrahydro-1H-indazol-1-yl)benzamide ***(PAP_N1 DS-biot)***

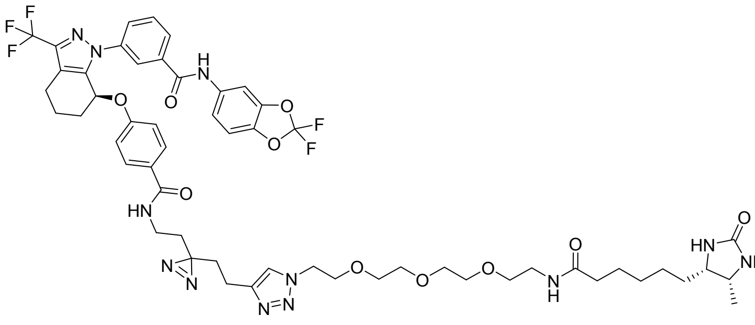

MW: 1135.14

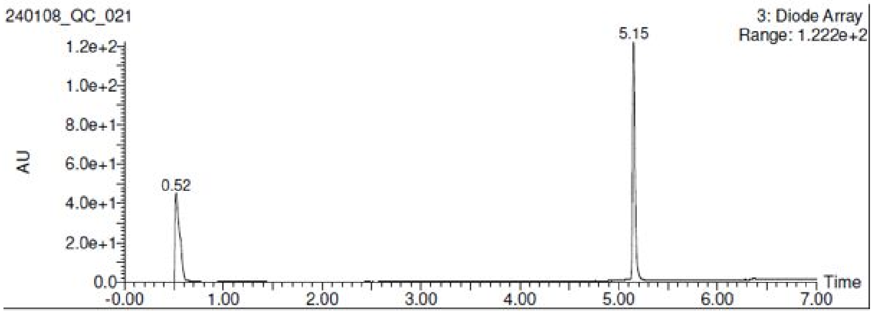

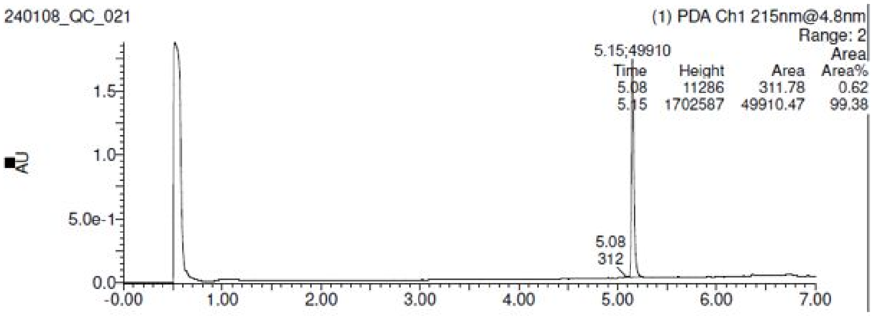

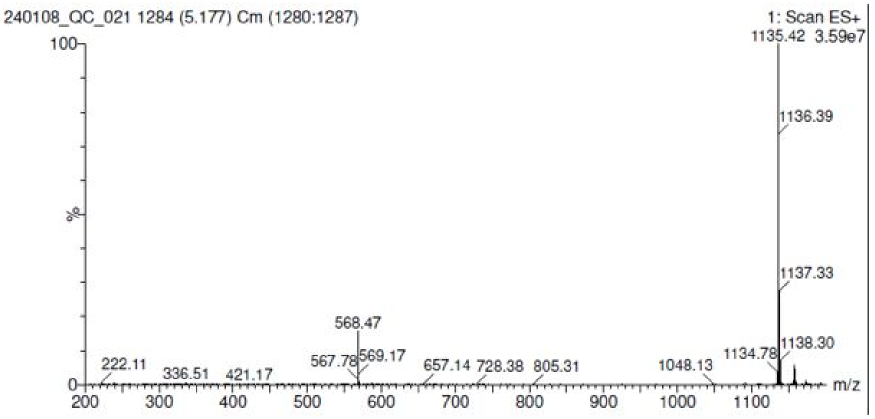

*N-Methyl-3-((S)-7-(4-(1-(18-((4S,5R)-5-methyl-2-oxoimidazolidin-4-yl)-13-oxo-3,6,9-trioxa-12-azaoctadecyl)-1H-1,2,3-triazol-4-yl)phenoxy)-3-(trifluoromethyl)-4,5,6,7-tetrahydro-1H-indazol-1-yl)-N-(4-(3-(trifluoromethyl)-3H-diazirin-3-yl)phenyl)benzamide (**PAP_2 DS-biot**)*

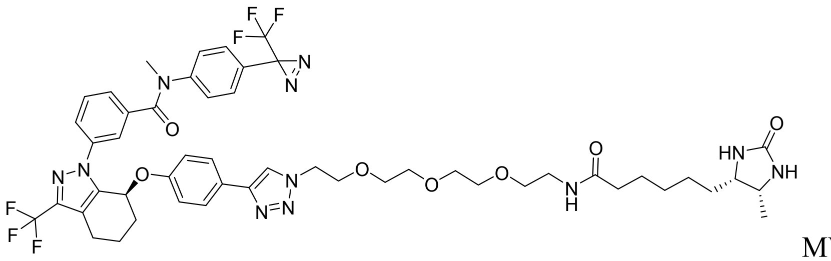

MW: 1038.04

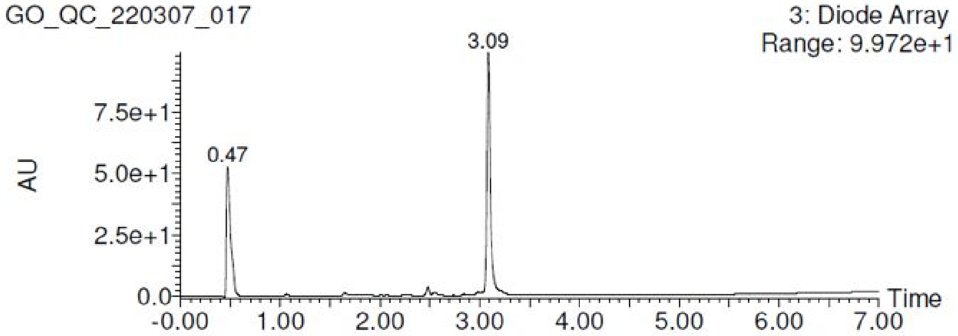

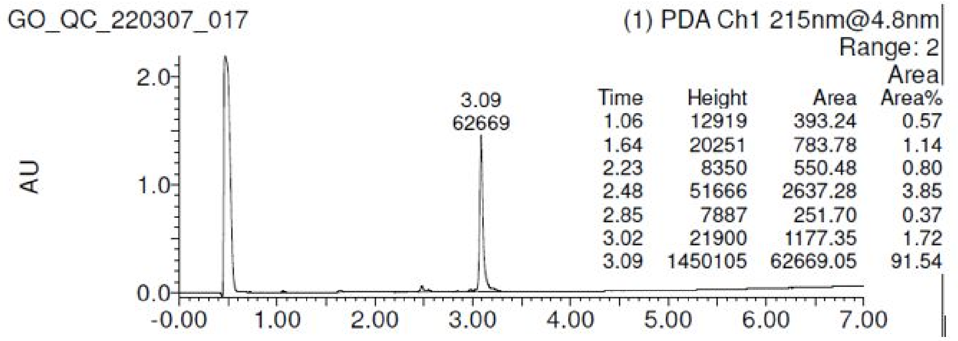

Time	Height	Area	Area%
1.06	12919	393.24	0.57
1.64	20251	783.78	1.14
2.23	8350	550.48	0.80
2.48	51666	2637.28	3.85
2.85	7887	251.70	0.37
3.02	21900	1177.35	1.72
3.09	1450105	62669.05	91.54

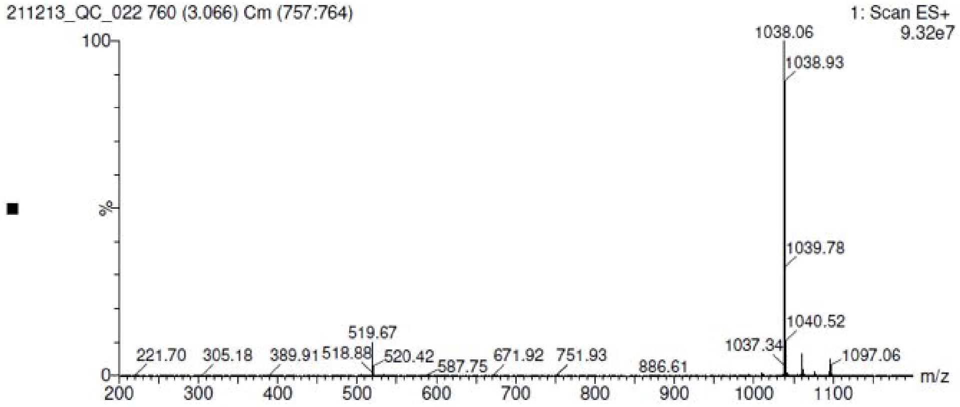

*3-((S)-7-(4-(1-(18-((4S,5R)-5-Methyl-2-oxoimidazolidin-4-yl)-13-oxo-3,6,9-trioxa-12-azaoctadecyl)-1H-1,2,3-triazol-4-yl)phenoxy)-3-(trifluoromethyl)-4,5,6,7-tetrahydro-1H-indazol-1-yl)-N-(4-(3-(trifluoromethyl)-3H-diazirin-3-yl)phenyl)benzamide (**PAP_N2 DS-biot**)*

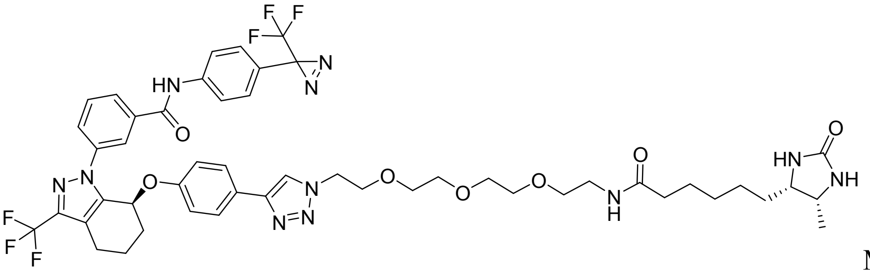

MW: 1024.02

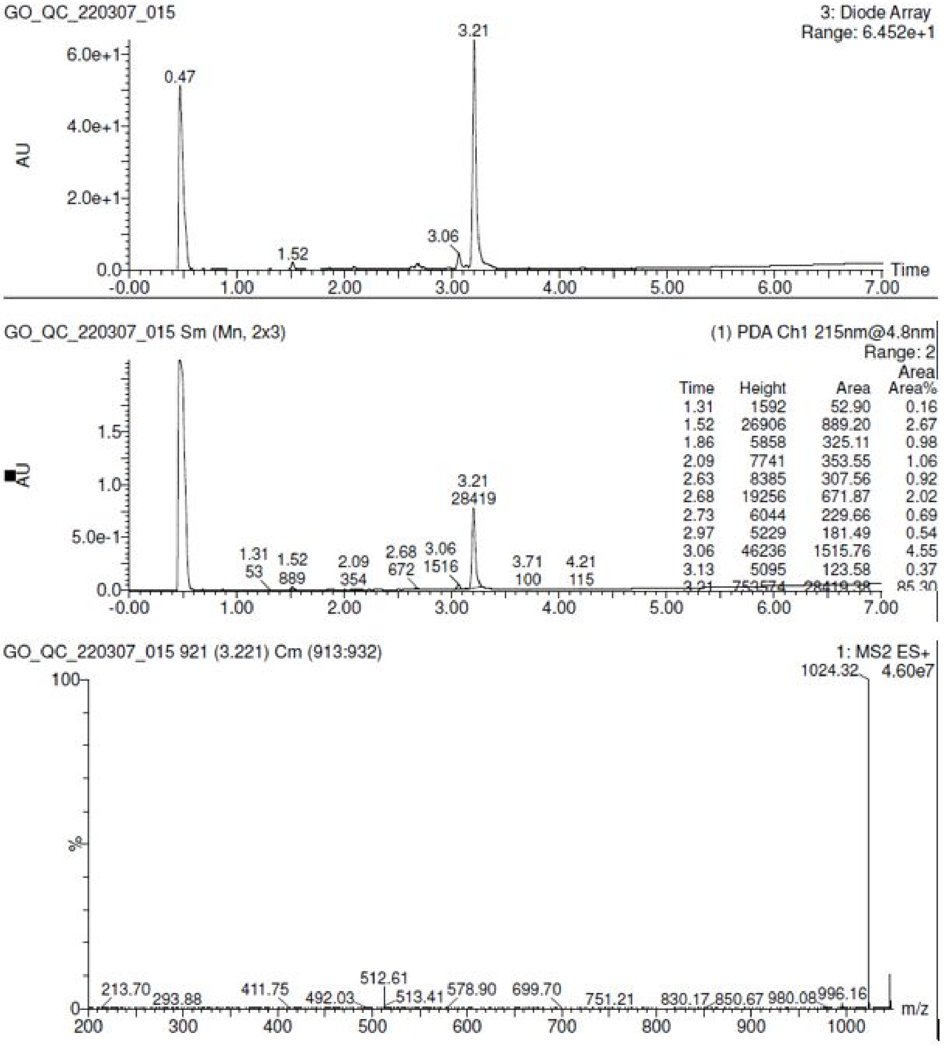

6. REFERENCES

1. Yang, H.; Shelat, A. A.; Guy, R. K.; Gopinath, V. S.; Ma, T.; Du, K.; Lukacs, G. L.; Taddei, A.; Folli, C.; Pedemonte, N.; Galietta, L .J.; Verkman, A. S. Nanomolar Affinity Small Molecule Correctors of Defective Delta F508-CFTR Chloride Channel Gating. *J. Biol. Chem.* **2003**, *278*, 35079-35085.
2. Fiedorczuk, K.; Chen, J. Mechanism of CFTR Correction by Type I Folding Correctors. *Cell* **2022**, *185*, 158-168.e11; Fiedorczuk, K.; Chen, J. Molecular Structures Reveal Synergistic Rescue of Δ508-CFTR by Trikafta Modulators. *Science* **2022**, *378*, 284-290.
3. Laselva, O.; Stone, T.A.; Bear, C. E.; Deber, C. M. Anti-Infectives Restore ORKAMBI® Rescue of F508del-CFTR Function in Human Bronchial Epithelial Cells Infected with Clinical Strains of P. Aeruginosa. *Biomolecules* **2020**, *10*, 334.
4. Laselva, O.; Molinski, S.; Casavola, V.; Bear, C. E. Correctors of the Major Cystic Fibrosis Mutant Interact through Membrane-Spanning Domains. *Mol. Pharmacol.* **2018**, *93*, 612-618.
5. Wu, P. S.; Otting, G. Rapid Pulse Length Determination in High-Resolution NMR. *J. Magn. Reson.* **2005,** *176*, 115-119.
6. Wider, G.; Dreier, L. Measuring Protein Concentrations by NMR Spectroscopy. *J. Am. Chem. Soc.* **2006,** *128*, 2571-2576.
